# Global Skin Disease Morbidity and Mortality: An Update From the Global Burden of Disease Study 2013

**DOI:** 10.1001/jamadermatol.2016.5538

**Published:** 2017-03-01

**Authors:** Chante Karimkhani, Robert P. Dellavalle, Luc E. Coffeng, Carsten Flohr, Roderick J. Hay, Sinéad M. Langan, Elaine O. Nsoesie, Alize J. Ferrari, Holly E. Erskine, Jonathan I. Silverberg, Theo Vos, Mohsen Naghavi

**Affiliations:** 1 University Hospitals Case Western Medical Center, Cleveland, Ohio; 2 now with Department of Dermatology, University of Colorado, Denver; 3 Dermatology Service, US Department of Veterans Affairs, Eastern Colorado Health Care System, Denver; 4 University of Colorado School of Medicine, Aurora; 5 Colorado School of Public Health, Department of Epidemiology, University of Colorado Anschutz Medical Campus, Aurora; 6 Institute for Health Metrics and Evaluation, University of Washington, Seattle; 7 Department of Public Health, Erasmus MC, University Medical Center Rotterdam, Rotterdam, the Netherlands; 8 Unit for Population-Based Dermatology Research, St John’s Institute of Dermatology, Guy’s & St Thomas’ NHS Foundation Trust and King’s College London, London, United Kingdom; 9 Department of Dermatology, Kings College NHS Trust, London, United Kingdom; 10 Faculty of Epidemiology and Population Health, London School of Hygiene and Tropical Medicine, London, United Kingdom; 11 School of Public Health, University of Queensland, Herston, Queensland, Australia; 12 Queensland Centre for Mental Health Research, Wacol, Queensland, Australia; 13 Department of Dermatology, Northwestern University Feinberg School of Medicine, Chicago, Illinois; 14 Department of Preventive Medicine, Northwestern University Feinberg School of Medicine, Chicago, Illinois; 15 Department of Medical Social Sciences, Northwestern University Feinberg School of Medicine, Chicago, Illinois

## Abstract

**Importance:**

Disability secondary to skin conditions is substantial worldwide. The Global Burden of Disease Study 2013 includes estimates of global morbidity and mortality due to skin diseases.

**Objective:**

To measure the burden of skin diseases worldwide.

**Data Sources:**

For nonfatal estimates, data were found by literature search using PubMed and Google Scholar in English and Spanish for years 1980 through 2013 and by accessing administrative data on hospital inpatient and outpatient episodes. Data for fatal estimates were based on vital registration and verbal autopsy data.

**Study Selection:**

Skin disease data were extracted from more than 4000 sources including systematic reviews, surveys, population-based disease registries, hospital inpatient data, outpatient data, cohort studies, and autopsy data. Data metrics included incidence, prevalence, remission, duration, severity, deaths, and mortality risk.

**Data Extraction and Synthesis:**

Data were extracted by age, time period, case definitions, and other study characteristics. Data points were modeled with Bayesian meta-regression to generate estimates of morbidity and mortality metrics for skin diseases. All estimates were made with 95% uncertainty intervals.

**Main Outcomes and Measures:**

Disability-adjusted life years (DALYs), years lived with disability, and years of life lost from 15 skin conditions in 188 countries.

**Results:**

Skin conditions contributed 1.79% to the global burden of disease measured in DALYs from 306 diseases and injuries in 2013. Individual skin diseases varied in size from 0.38% of total burden for dermatitis (atopic, contact, and seborrheic dermatitis), 0.29% for acne vulgaris, 0.19% for psoriasis, 0.19% for urticaria, 0.16% for viral skin diseases, 0.15% for fungal skin diseases, 0.07% for scabies, 0.06% for malignant skin melanoma, 0.05% for pyoderma, 0.04% for cellulitis, 0.03% for keratinocyte carcinoma, 0.03% for decubitus ulcer, and 0.01% for alopecia areata. All other skin and subcutaneous diseases composed 0.12% of total DALYs.

**Conclusions and Relevance:**

Skin and subcutaneous diseases were the 18th leading cause of global DALYs in Global Burden of Disease 2013. Excluding mortality, skin diseases were the fourth leading cause of disability worldwide.

## Key Points

**Question:**

What is the burden of skin disease worldwide?

**Findings:**

In this observational study, skin diseases contributed 1.79% to the global burden of disease measured in disability-adjusted life years (DALYs). Skin diseases arranged in order of decreasing global DALYs are as follows: dermatitis (atopic, contact, seborrheic), acne vulgaris, urticaria, psoriasis, viral skin diseases, fungal skin diseases, scabies, melanoma, pyoderma, cellulitis, keratinocyte carcinoma, decubitus ulcer, and alopecia areata.

**Meaning:**

Skin diseases remain a major cause of disability worldwide. An objective measure of burden, such as the DALY, allows for comparison of diverse diseases across geography and time.

## Teaser

This study measures the burden of skin diseases worldwide.

## Introduction

Global disability and mortality due to skin disease has been investigated by the Global Burden of Disease (GBD) 2013 Study. The GBD is a collaboration of more than 1000 experts worldwide, aiming to create a systematic, quantified, and internally consistent source of health information.^1^ Based at the Institute for Health Metrics and Evaluation and funded by the Bill and Melinda Gates Foundation, GBD 2013 provides disability and mortality metrics for diseases, injuries, and risk factors stratified by age, sex, location, and time.^2^ Disease burden was estimated using the metric of disability-adjusted life year (DALY), which is the sum of years of life lost to a disease (YLLs) plus years lived with disability (YLDs). One DALY is equivalent to 1 year of healthy life lost.^4^^(pp6-7)^ This measurement unit allows for cross-comparison of diverse disease states. The goal of GBD is to produce the highest-quality epidemiologic data by ensuring transparent analytic strategies that include uncertainty distributions, promote internal consistency, and allow iterative revisions over time. Each GBD iteration incorporates new studies and improved methodology, creating a “living” database to inform clinical, research-oriented, and policy-making decisions. This rolling design of the data set allows for the addition of new data sources and improvements to the methods of estimation. Global Burden of Disease 2013 made estimates for 306 diseases and injuries in 188 countries. As GBD researchers and collaborators, we present results for 15 dermatologic conditions.

## Methods

The analyses were carried out by the team members of the Institute of Health Metrics and international skin experts with an interest in dermatoepidemiology. The following skin conditions were selected based on available data, standardized disease definitions, and prevalence: dermatitis (including the common varieties of eczema: atopic, seborrheic, and contact dermatitis), psoriasis, cellulitis, pyoderma, scabies, fungal skin diseases, viral skin diseases, acne vulgaris, alopecia areata, pruritus, urticaria, decubitus ulcer, malignant skin melanoma, and keratinocyte carcinoma (including basal and squamous cell carcinomas). An additional category, “other skin and subcutaneous diseases,” encompasses the remainder of miscellaneous skin conditions.^5^ *International Classification of Diseases, Ninth Revision (ICD-9)* and *ICD-10* codes were used to define each of the 15 skin disease categories (eTable 1 in the Supplement).

The initial step in the GBD estimation strategy was a thorough investigation of the world literature using PubMed and Google Scholar for data on the incidence, prevalence, remission, duration, severity, and mortality risk (only applicable for selected skin diseases) of the 15 skin conditions. Data were extracted by age, time period, and with information defining uncertainty (standard error, confidence interval, or numerator/denominator). Searches were performed in English and Spanish languages for the years 1980 through 2013. Data were extracted from more than 4000 sources including systematic reviews, surveys, population-based disease registries, hospital inpatient data, outpatient data, and cohort studies. Most incidence data for certain skin diseases were obtained from 3 medical record sources: (1) inpatient data from Europe, Latin America, and the United States, (2) outpatient data predominantly from the United States, and (3) in the case of basal cell and squamous cell carcinoma of the skin, registry data where it included keratinocyte carcinoma, for example, Northern Ireland Cancer Registry, 2010 (see eTable 2 in the Supplement for all GBD 2013 skin disease data sources).

Data points from the aforementioned literature search were analyzed in a Bayesian meta-regression modeling tool, DisMod-MR 2.0, to yield prevalence estimates that are consistent with the other available epidemiological parameters for each of the skin conditions. All estimates were generated with 1000 draws from the posterior distribution of the quantity of interest, which allows for generation of 95% uncertainty intervals. Compared with the previous GBD 2010 study, DisMod-MR was recoded and optimized to run up to 50 times faster and shifted from a negative binomial to an offset log-normal model.^6^

The literature search for GBD 2013 compared with GBD 2010 doubled the data set for psoriasis with an additional 30 prevalence and 5 incidence studies. Psoriasis was also modeled with a smaller remission assumption (0.05-0.15 remitted cases per case per year), better reflecting a chronic disease pattern. Of note, the GBD definition of remission is the rate at which cases stop fulfilling the case definition, that is, cure. For cellulitis, 13 191 incidence data points from both inpatient and outpatient samples were added. No additional data sources were added for pyoderma in GBD 2013. However, duration of disease was decreased from approximately 1 year in GBD 2010 to between 2 weeks for treated disease to 4 weeks for untreated disease in GBD 2013. For fungal skin diseases, prevalence was estimated separately for tinea capitis and “other fungal skin diseases.” Similarly, prevalences of viral warts and molluscum contagiosum were modeled separately and then summarized as “viral skin diseases.” In GBD 2010, studies for “itch” were used as prevalence of pruritus, which inaccurately included cases of pruritic skin and nonskin conditions. Comparatively, GBD 2013 included only outpatient data from Norway and the United States and several data points from the literature of patients with a diagnosis of pruritus (excluding known causes of itch). While no additional data sources were added, acne vulgaris modeling in GBD 2013 no longer assumes that reported data include asymptomatic cases because most data points were based on examination and therefore would reflect symptomatic cases. For alopecia areata, GBD 2013 applied lower estimates from the largest data source (outpatient data from the United States). No new data sources for urticaria were added; however, similarly to the case for acne vulgaris, GBD 2013 urticaria data points reflect solely symptomatic cases. For decubitus ulcer, input data were incidence from hospital admission and outpatient data. In GBD 2010, implausible duration estimates led to an overestimate of prevalence, which has been corrected in GBD 2013. Global Burden of Disease 2013 set bounds on the remission rate from 0.5 to 6 corresponding to duration range of 2 months to 2 years.

Years lived with disability due to skin cancers were estimated for 4 different sequelae: (1) diagnosis and treatment, (2) remission, (3) metastatic disease, and (4) terminal disease. The survival timeframe was extended from 5 to 10 years. The duration of diagnosis and treatment for keratinocyte carcinoma was taken as the same as for melanoma from Neal et al^7^ (duration until diagnosis plus 2 months for treatment). As defined by Neal et al,^7^ duration until diagnosis included days from first presentation in general practice (GP) to referral or GP biopsy, days from referral to first seen in clinic, days from first seen in clinic to biopsy/wide local excision for GP excisions, and days from presentation to biopsy/wide local excision for GP excisions. Duration of the remission sequela is based on time until death or 5 years for survivors minus duration of the other sequelae. Duration of disseminated disease was based on Nolan et al^8^ for lethal keratinocyte skin cancers and on Surveillance, Epidemiology, and End Results^9^ analysis of median survival for patients with stage IV melanoma.

Prevalence estimates from the estimation strategy were combined with disability weights to yield skin disease morbidity, expressed in YLDs, for each age-sex-country-year group. Disability weights, which range from 0 to 1 for each condition, were derived from 4 population-based European surveys (30 660 respondents) and the GBD 2010 disability weight surveys (30 230 respondents) eliciting response to the question “who is the healthier?” for randomly chosen pairs of health states. Health states were presented with a short lay description. For parsimony, a smaller number (235) of health states were designed to cover the spectrum of disability across all 2337 disease sequelae. The most commonly used health states for skin disease were 3 levels of severity of disfigurement with or without itch and/or pain.^10^ The lay descriptions for disfigurement assessed in the disability weight surveys include psychological morbidity attributable to each skin disease. The mild infectious disease health state was also used for bacterial, fungal, and viral skin diseases. See Table 1 for descriptions of health states used to generate disability weights for skin diseases.

**Table 1.** Skin Disease Disfigurement Health States and Disability Weights

Disfigurement Health State	Description	Disability Weight Value (95% CI)
Disfigurement, level 1	This person has a slight, visible physical deformity that others notice, which causes some worry and discomfort.	0.011 (0.005-0.021)
Disfigurement, level 2	This person has a visible physical deformity that causes others to stare and comment. As a result, the person is worried and has trouble sleeping and concentrating.	0.067 (0.044-0.096)
Disfigurement, level 3	This person has an obvious physical deformity that makes others uncomfortable, which causes the person to avoid social contact, feel worried, sleep poorly, and think about suicide.	0.405 (0.275-0.546)
Disfigurement, level 1 with itch/pain	This person has a slight, visible physical deformity that is sometimes sore or itchy. Others notice the deformity, which causes some worry and discomfort.	0.027 (0.015-0.042)
Disfigurement, level 2, with itch/pain	This person has a visible physical deformity that is sore and itchy. Other people stare and comment, which causes the person to worry. The person has trouble sleeping and concentrating.	0.188 (0.125-0.267)
Disfigurement, level 3, with itch/pain	This person has an obvious physical deformity that is very painful and itchy. The physical deformity makes others uncomfortable, which causes the person to avoid social contact, feel worried, sleep poorly, and think about suicide.	0.576 (0.401-0.731)
Infectious disease, acute episode, mild	This person has a low fever and mild discomfort, but no difficulty with daily activities.	0.006 (0.002-0.017)

Deaths and YLLs were estimated for the following 6 skin categories: malignant skin melanoma, keratinocyte carcinoma, cellulitis, pyoderma, decubitus ulcer, and other skin and subcutaneous diseases. The Cause of Death Ensemble Model (CODEm) tool was used to produce mortality estimates based on data on causes of death from the extensive GBD collection of vital registration and verbal autopsy data.^11^ The CODEm strategy uses a range of plausible models and predictive covariates for each cause and chooses the best-performing models to yield predictions for cause-specific death estimates with uncertainty intervals. The YLLs for each age-sex-country group are the multiplication of death counts at the age at death by remaining life expectancy from the GBD standard life table that is applied equally to all countries and periods.^2^ Mortality from the remaining skin conditions was assumed to be 0.

Morbidity (YLD) and mortality (YLL) estimates were added for each age-country-sex group to yield DALYs, which are reported as numbers, percent of total DALYs from all conditions studied by GBD, and the DALY rate per 100 000 persons. Estimates were made for both sexes and 21 age groups ranging from the first week of life to older than 80 years, as well as age-standardized estimates. Age standardization was applied based on the standard population structure of population in 2010 through 2035 as estimated for GBD 2013 based on the most recent World Population Prospects by the United Nations Population Division.^11^ Finally, the GBD analysis was computed for 188 countries grouped into 21 GBD world regions.^13^ The Global Burden of Disease Study has institutional review board approval through March 25, 2018, from the University of Washington.

## Results

Skin and subcutaneous diseases were responsible for 41.6 million DALYs and 39.0 million YLDs in 2013. The age-standardized percent change in DALY rate from 2005 to 2013 was 0.1%. Table 2 lists age-standardized DALY rates per 100 000 persons, percent change in DALY rates from 2005 to 2013, and all-age DALYs for the 14 skin conditions and the other skin and subcutaneous diseases category. Results are presented in order of decreasing DALY rate. Dermatitis, which includes estimates for atopic, seborrheic, and contact dermatitis, is responsible for the largest global burden of DALYs and YLDs. While cellulitis causes an intermediate global burden compared with the other skin conditions, it experienced the greatest decline from 2005 to 2013 and was the only skin condition with a significant change. This change was due to a decrease in death estimates.

**Table 2.** Age-Standardized Disability-Adjusted Life Year (DALY) Rates per 100 000 Persons, Percent Change in DALY Rate From 2005-2013, and DALYs for the 15 Skin Conditions

Skin Condition	Age-Standardized DALY Rate per 100 000 Persons (95% UI)	Change in DALY Rate 2005-2013, % (95% UI)	DALYs, Millions (95% UI)	YLDs (95% UI)
Dermatitis	128.7 (83.6 to 184.9)	0.1 (−0.4 to 0.6)	9.3 (6.0 to 13.3)	9.3 (6.0 to 13.3)
Acne vulgaris	96.7 (46.4 to 177.8)	−0.5 (−6.5 to 7.7)	7.2 (3.4 to 13.2)	7.2 (3.4 to 13.2)
Urticaria	67.0 (43.2 to 95.5)	4.3 (−9.4 to 16.1)	4.7 (3.0 to 6.7)	4.7 (3.0 to 6.7)
Psoriasis	66.8 (46.0 to 93.6)	−0.5 (−1.6 to 0.6)	4.7 (3.2 to 6.6)	4.7 (3.2 to 6.6)
Viral skin diseases	54.7 (33.3 to 85.0)	−0.6 (−1.5 to 0.3)	4.0 (2.4 to 6.2)	4.0 (2.4 to 6.2)
Fungal skin diseases	54.0 (22.1 to 114.2)	1.0 (0.5 to 1.4)	3.8 (1.6 to 8.1)	3.8 (1.6 to 8.1)
Scabies	23.5 (13.3 to 37.3)	−2.8 (−10.2 to 6.7)	1.7 (0.97 to 1.7)	1.7 (0.97 to 1.7)
Melanoma	23.2 (18.1 to 31.1)	−6.1 (−13.0 to −0.8)	1.6 (1.2 to 2.1)	0.14 (0.092 to 0.22)
Pyoderma	16.6 (13.0 to 19.3)	6.4 (−7.2 to 21.3)	1.1 (0.89 to 1.3)	0.033 (0.012 to 0.072)
Cellulitis	15.5 (11.8 to 20.2)	−13.2 (−21.1 to −1.7)	1.1 (0.81 to 1.4)	0.12 (0.079 to 0.17)
Keratinocyte carcinoma	12.9 (10.8 to 16.3)	−6.2 (−10.7 to 0.0)	0.82 (0.68 to 1.0)	0.13 (0.082 to 0.19)
Decubitus ulcer	10.8 (9.1 to 12.7)	−0.8 (−5.8 to 4.6)	0.66 (0.55 to 0.78)	0.28 (0.20 to 0.37)
Alopecia areata	4.2 (2.7 to 6.3)	−0.1 (−2.9 to 2.7)	0.29 (0.19 to 0.43)	0.29 (0.19 to 0.43)
Pruritus	0.2 (0.1 to 0.3)	0.9 (−6.2 to 8.6)	0.011 (0.0051 to 0.020)	0.0011 (0.0051 to 0.0020)
Other skin and subcutaneous diseases	44.2 (19.8 to 93.3)	0.4 (−0.7 to 1.6)	3.0 (1.4 to 6.2)	2.9 (1.3 to 6.1)

Abbreviations: UI, uncertainty interval; YLDs, years lived with disability.

**Figure 1.**
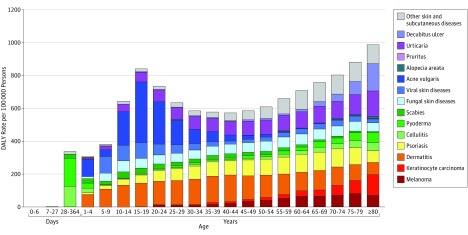
Age Distribution of Skin and Subcutaneous Disease Burden This figure shows disability-adjusted life year (DALY) rate per 100 000 persons from 15 skin disease categories throughout the human life span.

The age-stratified breakdown for the skin conditions is shown in Figure 1. Due to age restrictions, estimates for patients younger than 1 year were only available for the following conditions: cellulitis, pyoderma, scabies, and fungal and viral skin diseases. In addition, the age categories of 1 to 4, 5 to 9, and 10 to 14 years had no DALY estimates for melanoma or keratinocyte carcinoma. Dermatitis burden remains relatively consistent throughout all age categories, in comparison with acne vulgaris, which causes the greatest burden between the first and third decades of life. Skin conditions with the greatest burden in younger ages also include infectious causes, such as viral skin diseases (mostly viral warts), bacterial skin diseases (pyoderma and cellulitis), and scabies. Burden from psoriasis, alopecia areata, urticaria, fungal skin diseases, and decubitus ulcer is greater in older age categories. Keratinocyte carcinoma and melanoma burden increase over the human age-span, with the greatest DALY rates in those older than 75 years.

**Figure 2.**
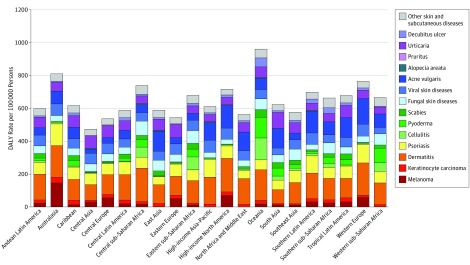
Regional Distribution of Skin and Subcutaneous Disease Burden This figure shows disability-adjusted life year (DALY) rate per 100 000 persons from 15 skin disease categories throughout 21 world regions.

Melanoma causes the greatest burden in Australia, followed by high-income North America, Western Europe, and Central Europe (Figure 2). Similarly, keratinocyte carcinoma causes the greatest burden in Australasia, the Caribbean, Central Latin America, and tropical Latin America. Dermatitis burden follows a similar geographic predominance, with a high DALY rate in central sub-Saharan Africa. Psoriasis causes the greatest burden in Australasia, Western Europe, high-income Asia-Pacific, and southern Latin America. Burden from acne vulgaris is greatest in Western Europe, high-income North America, and southern Latin America. Central, western, and eastern sub-Saharan Africa, along with Oceania, have the highest DALY rates from cellulitis. Burden from urticaria is evenly distributed among world regions. Decubitus ulcer has the greatest DALY rate in Oceania.

## Discussion

The importance of the skin disease global burden can be appreciated by comparing the skin results presented here as DALYs or YLDs with the 158 disease and injury categories at level 3 of the GBD 2013 hierarchy.^14^ Comparing absolute DALYs and/or YLDs, skin and subcutaneous diseases were the 18th leading cause of global DALYs and the fourth leading cause of nonfatal burden in GBD 2013. Burden from skin diseases (41.0 million DALYs) ranked directly behind iron deficiency anemia (43.7 million DALYs), tuberculosis (49.8 million DALYs), and sense organ diseases (54.4 million DALYs). As a reference point, the leading cause of global DALYs over the past decade has been ischemic heart disease, responsible for 150.2 million all-age DALYs in 2013. Excluding mortality, YLDs from skin diseases (36.4 million) are larger than those caused by diabetes mellitus (29.5 million) and migraines (28.9 million).

A commitment to accurate, transparent, and frequently updated metrics of disease burden has the potential to affect diverse levels of health care and further the role of dermatology in global health. The objectives of GBD are aligned with efforts of international organizations such as the World Health Organization and the United Nations.^16^ An example of dermatology research and leadership as a global collaboration is the International Federation of Dermatology Clinical Trials Network.^17^ This organization promotes the development of standardized and transparent clinical trials in dermatology, independent of industry. Dermatologists have an important role in this global health landscape. Not only do skin diseases cause substantial pain, disfigurement, and psychological and financial morbidity, but dermatologic findings are often the initial manifestation of systemic disease.^18^ Global Burden of Disease metrics and patterns have the potential to affect clinical trials on a global scale.

Dermatological research, education, clinical application, and local and national health support are critical tenets of dermatology’s role on the global health platform.^19^ As an example, the International Foundation for Dermatology is an international organization founded in 1987 that aims to improve dermatologic care in underserved areas of low- and middle-income countries.^21^ A particular focus has been keratinocyte carcinoma in tropical regions. Global Burden of Disease 2013 demonstrates high incidence and burden of keratinocyte carcinoma in southern sub-Saharan Africa (including Botswana, Lesotho, Namibia, South Africa, Swaziland, and Zimbabwe), which is hypothesized to be particularly due to mortality from keratinocyte carcinoma reflecting poor access to health care and high prevalence of human immunodeficiency virus infection and albinism.^22^ The International Foundation for Dermatology has identified a particular vulnerable group in the tropics, persons with albinism (PWA), who are predisposed to premature skin cancer morbidity and mortality.^24^ Achievements from the PWA Working Group include skin cancer prevention, education, and the development of novel sunscreen products.

A final example of the impact of disease burden in dermatology is the Cochrane Collaboration. Composed of 53 review groups and members across the world, the organization conducts systematic reviews of randomized clinical trials for a diverse array of health care topics and interventions.^25^ The Cochrane Skin Group focuses on various aspects of skin disease diagnosis, management, and prevention and specifically includes burden of disease in priority setting for new review topics.^26^ For example, as demonstrated by GBD 2013, the greatest global burden from skin disease is attributed to dermatitis. Of the skin diseases studied by GBD 2013, dermatitis had the greatest representation in Cochrane systematic reviews and protocols.^27^ Other factors for review prioritization include, but are not limited to, knowledge gap, capacity building, opportunity for scientific innovation, equity, existence of other systematic reviews, availability of public funding, health importance, and cost-effectiveness.

### Limitations

Global Burden of Disease has applied global efforts and sophisticated statistical approaches to make best estimates from raw data. As such, the study’s limitations include sparse data for some global areas due in part to the use of coding systems other than* ICD *in primary care that limited skin disease data availability. Global Burden of Disease methodology depends on statistical methods to generate predictions for areas where direct prevalence estimates for a particular disease are not available. For countries, years, ages, and sexes without data, predictions are generated by deriving information from data in nearby countries and regions and, where available, predictive covariates. This analytical strategy has the limitation that it is based on assumptions informed by either current knowledge of disease risk factors, country-level covariates, or, more commonly, geographical region averages. For most skin diseases, no country-level covariates were used, as there is limited knowledge about risk factors. Thus, predictions rely solely on data for countries in the same region or superregion. In addition, GBD estimates are inherently dependent on accurate interpretation of *ICD* codes and case definitions used by studies contributing data. With each iteration of GBD, disease classification methods become more standardized and can become a guide to epidemiologists collecting empirical data on preferred case definitions and study methods.

## Conclusions

The GBD collaboration has recently emerged as the first nongovernmental organization to affect the global landscape of health metrics.^28^ Patterns of disease burden can be used to correlate with disease pathogenesis. With the worldwide collaboration of experts; attention to diverse causes, risk factors, ages, and world regions; and sophisticated analytical modeling tools, GBD 2013 has established a standardized method for global health metrics. This advancement has important implications for the field of public health and clinical medicine, including dermatology, as it will aid research priority-setting decisions and public policy efforts at local and national levels.

## Notes

Supplement. **eTable 1.** ICD-9 and -10 code definitions of 15 skin disease categories **eTable 2.** GBD 2013 skin disease data sources
