# Supplementary material for: Global Skin Disease Morbidity and Mortality: An Update From the Global Burden of Disease Study 2013
Source: JAMA Dermatol. 2017 Mar 1;153(5):406–12. doi: 10.1001/jamadermatol.2016.5538 (PMC5817488; doi:10.1001/jamadermatol.2016.5538)
Supplement: Supplement. — eTable 1. ICD-9 and -10 code definitions of 15 skin disease categories eTable 2. GBD 2013 skin disease data sources [file jamadermatol-153-406-s001.pdf]

Supplementary Online Content

Karimkhani C, Dellavalle RP, Coffeng LE, et al. Global skin disease morbidity and mortality: an update from the Global Burden of Disease Study 2013. *JAMA Dermatol*. Published online March 1, 2017. doi:10.1001/jamadermatol.2016.5538

eTable 1. ICD-9 and -10 code definitions of 15 skin disease categories
eTable 2. GBD 2013 skin disease data sources

This supplementary material has been provided by the authors to give readers additional information about their work.

© 2017 Karimkhani C et al. *JAMA Dermatology.*

eTable 1. ICD-9 and -10 code definitions of 15 skin disease categories

**Skin disease**	**ICD-10 codes**	**ICD-9 codes**
Dermatitis	L20-L23.2, L23.4-L24.3, L24.5-L25.0, L25.2-L27, L27.2-L27.9	690-692
Psoriasis	L40-L41.9	696
Cellulitis	L03-L04	681, 682
Pyoderma	A31.1, A31.2, A46, L00-L02.93, L04.0-L05.92, L08-L08.9, L88, L97-L98.499	680, 683, 684, 686, 707.1, 707.8, 707.9
Scabies	B86	133.0
Fungal skin diseases	B35-B36.9	110, 111
Viral skin diseases	B07-B09	78
Acne vulgaris	L70-L70.3, L70.8, L70.9, L73.0	706.0, 706.1
Alopecia areata	L63-L63.3, L63.8, L63.9	704.0
Pruritus	L29-L29.9	698
Urticaria	L50-L50.9	708
Decubitus ulcer	L89-L89.95	707
Keratinocyte carcinoma	C44-C44.9	173, 232, 238.2
Melanoma	C43-C43.9, C4A, Z85.82-Z85.828	172
Other skin and subcutaneous diseases	B85-B85.4, B87-B88.9, L10-L14, L28-L28.2, L30-L30.9, L42-L45, L49-L49.9 L51-L55.9, L56.1-L57.9, L59-L60.9, L62-L62.8, L64-L68.9, L70.4, L70.5, L71-L73, L73.1-L76.82, L80-L87.9, L90-L92.9, L94-L95.9, L98.5, L98.6	685, 693, 694, 695, 697, 700, 701, 702-706, 709

© 2017 Karimkhani C et al. *JAMA Dermatology.*

eTable 2. GBD 2013 skin disease data sources

Skin condition	Citation	Location	Years studied
Acne vulgaris	Nijsten T, Rombouts S, Lambert J. Acne is prevalent but use of its treatments is infrequent among adolescents from the general population. J Eur Acad Dermatol Venereol 2007;21(2):163-8.	Belgium	2004-2005
	Bechelli LM, Haddad N, Pimenta WP, Pagnano PM, Melchior E Jr, Fregnan RC, Zanin LC, Arenas A. Epidemiological survey of skin diseases in schoolchildren living in the Purus Valley (Acre State, Amazonia, Brazil). Dermatologica 1981;163(1):78-93.	Brazil	1974-1975
	Laczynski CMM, Cestari S da CP. Prevalence of dermatosis in scholars in the region of ABC paulista. An Bras Dermatol 2011;86(3):469-76.	Brazil	2006
	Bissek A-CZ-K, Tabah EN, Kouotou E, Sini V, Yepnjio FN, Nditanchou R, Nchufor RN, Defo D, Dema F, Fonsah JY, Njamnshi AK, Muna WFT. The spectrum of skin diseases in a rural setting in Cameroon (sub-Saharan Africa). BMC Dermatol 2012;7.	Cameroon	2010
	Shen Y, Wang T, Zhou C, Wang X, Ding X, Tian S, Liu Y, Peng G, Xue S, Zhou J, Wang R, Meng X, Pei G, Bai Y, Liu Q, Li H, Zhang J. Prevalence of acne vulgaris in Chinese adolescents and adults: a community-based study of 17,345 subjects in six cities. Acta Derm Venereol 2012;92(1):40-4.	China	2009-2011
	Abdel-Hafez K, Abdel-Aty MA, Hofny ERM. Prevalence of skin diseases in rural areas of Assiut Governorate, Upper Egypt. Int J Dermatol 2003;42(11):887-92.	Egypt	1994-1996
	Yamamah GA, Emam HM, Abdelhamid MF, Elsaie ML, Shehata H, Farid T, Kamel MI, Taalat AA. Epidemiologic study of dermatologic disorders among children in South Sinai, Egypt. Int J Dermatol 2012;51(10):1180-5.	Egypt	2008-2009
	Accorsi S, Barnabas GA, Farese P, Padovese V, Terranova M, Racalbuto V, Morrone A. Skin disorders and disease profile of poverty: analysis of medical records in Tigray, northern Ethiopia, 2005-2007. Trans R Soc Trop Med Hyg 2009;103(5):469-75.	Ethiopia	2005-2007
	Figueroa JI, Fuller LC, Abraha A, Hay RJ.	Ethiopia	1994

© 2017 Karimkhani C et al. *JAMA Dermatology.*

	Dermatology in southwestern Ethiopia: rationale for a community approach. Int J Dermatol 1998;37(10):752-8.		
	Leekassa R, Bizuneh E, Alem A, Fekadu A, Shibre T. Community diagnosis of common skin diseases in the Zay community of the Zeway Islands, Ethiopia. Ethiop Med J 2005;43(3):189-95.	Ethiopia	1998
	Poli F, Dreno B, Verschoore M. An epidemiological study of acne in female adults: results of a survey conducted in France. J Eur Acad Dermatol Venereol 2001;15(6):541-5.	France	1998
	Augustin M, Herberger K, Hintzen S, Heigel H, Franzke N, Schäfer I. Prevalence of skin lesions and need for treatment in a cohort of 90,880 workers. Br J Dermatol 2011;165(4):865-73.	Germany	2002-2009
	Schaefer I, Rustenbach SJ, Zimmer L, Augustin M. Prevalence of skin diseases in a cohort of 48,665 employees in Germany. Dermatology (Basel) 2008;217(2):169-72.	Germany	2001-2005
	Schäfer T, Nienhaus A, Vieluf D, Berger J, Ring J. Epidemiology of acne in the general population: the risk of smoking. Br J Dermatol 2001;145(1):100-4.	Germany	1998-2000
	Hogewoning AA, Koelemij I, Amoah AS, Bouwes Bavinck JN, Aryeetey Y, Hartgers F, Yazdanbakhsh M, Willemze R, Boakye DA, Lavrijsen APM. Prevalence and risk factors of inflammatory acne vulgaris in rural and urban Ghanaian schoolchildren. Br J Dermatol 2009;161(2):475-7.	Ghana	2007
	Tasoula E, Gregoriou S, Chalikias J, Lazarou D, Danopoulou I, Katsambas A, Rigopoulos D. The impact of acne vulgaris on quality of life and psychic health in young adolescents in Greece. Results of a population survey. An Bras Dermatol 2012;87(6):862-9.	Greece	2007-2009
	Fung WK, Lo KK. Prevalence of skin disease among school children and adolescents in a Student Health Service Center in Hong Kong. Pediatr Dermatol 2000;17(6):440-6.	Hong Kong Special Administrative Region of China	1996-1997
	Grover S, Ranyal RK, Bedi MK. A cross section of skin diseases in rural Allahabad. Indian J Dermatol 2008;53(4):179-81.	India	2005
	Al-Rubiay KK, Al-Rubaiy LK. Dermatoepidemiology: A Household Survey Among Two Urban Areas In Basrah City, Iraq. Internet J Dermatol 2006;4(2):10.	Iraq	2005

© 2017 Karimkhani C et al. *JAMA Dermatology.*

	Hanisah A, Omar K, Shah SA. Prevalence of acne and its impact on the quality of life in school-aged adolescents in Malaysia. J Prim Health Care 2009;1(1):20-5.	Malaysia	2006-2008
	Hay RJ, Castanon RE, Hernandez HA, Lopez GC, Fuentes LF, Solis SP, Andersson N. Wastage of family income on skin disease in Mexico. BMJ 1994;309(6958):848.	Mexico	1993
	Walker SL, Shah M, Hubbard VG, Pradhan HM, Ghimire M. Skin disease is common in rural Nepal: results of a point prevalence study. Br J Dermatol 2008;158(2):334-8.	Nepal	2004-2006
	Lello J, Pearl A, Arroll B, Yallop J, Birchall NM. Prevalence of acne vulgaris in Auckland senior high school students. N Z Med J 1995;108(1004):287-9.	New Zealand	1993
	Purvis D, Robinson E, Watson P. Acne prevalence in secondary school students and their perceived difficulty in accessing acne treatment. N Z Med J 2004;117(1200):1018.	New Zealand	2001
	Ogunbiyi AO, Omigbodun Y, Owoaje E. Prevalence of skin disorders in school children in southwest Nigeria. Int J Adolesc Med Health 2009;21(2):235-41.	Nigeria	2006-2008
	Yahya H. Change in pattern of skin disease in Kaduna, north-central Nigeria. Int J Dermatol 2007;46(9):936-43.	Nigeria	2007
	Dalgard F, Gieler U, Holm JØ, Bjertness E, Hauser S. Self-esteem and body satisfaction among late adolescents with acne: results from a population survey. J Am Acad Dermatol 2008;59(5):746-51.	Norway	2004
	Halvorsen JA, Braae Olesen A, Thoresen M, Holm JØ, Bjertness E, Dalgard F. Comparison of self-reported skin complaints with objective skin signs among adolescents. Acta Derm Venereol 2008;88(6):573-7.	Norway	2006
	Bechelli LM, Haddad N, Pimenta WP, Pagnano PM, Melchior E Jr, Fregnan RC, Zanin LC, Arenas A. Epidemiological survey of skin diseases in schoolchildren living in the Purus Valley (Acre State, Amazonia, Brazil). Dermatologica 1981;163(1):78-93.	Peru	1995
	Freyre EA, Rebaza RM, Sami DA, Lozada CP. The prevalence of facial acne in Peruvian adolescents and its relation to their ethnicity. J Adolesc Health 1998;22(6):480-4.	Peru	1995

© 2017 Karimkhani C et al. *JAMA Dermatology.*

	Gutierrez EL, Galarza C, Ramos W, Tello M, Jiménez G, Ronceros G, Chía H, Hurtado J, Ortega-Loayza AG. Skin diseases in the Peruvian Amazonia. Int J Dermatol 2010;49(7):794-800.	Peru	2006-2008
	Gutierrez EL, Galarza C, Ramos W. Prevalencia de Enfermedades Dermatológicas en una comunidad rural de Ucayali, Perú. Dermatol Peru 2009;19(2):104-13.	Peru	2005
	Amado JM, Matos ME, Abreu AM, Loureiro L, Oliveira J, Verde A, Massa A. The prevalence of acne in the north of Portugal. J Eur Acad Dermatol Venereol 2006;20(10):1287-95.	Portugal	2005-2006
	Massa A, Alves R, Amado J, Matos E, Sanches M, Selores M, Santos C, Costa V, Velho G, Oliveira M, Ferreira E, Taveira M, Silva NS, Granado E, Lemos A, Calheiros JM. Prevalence of cutaneous lesions in Freixo de Espada à Cinta. Acta Med Port 2000;13(5-6):247-54.	Portugal	1998
	Popescu R, Popescu CM, Williams HC, Forsea D. The prevalence of skin conditions in Romanian school children. Br J Dermatol 1999;140(5):891-6.	Romania	1995
	Al-Saeed WY, Al-Dawood KM, Bukhari IA, Bahnassy AA. Prevalence and pattern of skin disorders among female schoolchildren in Eastern Saudi Arabia. Saudi Med J 2006;27(2):227-34.	Saudi Arabia	2003
	Amin TT, Ali A, Kaliyadan F. Skin disorders among male primary school children in Al Hassa, Saudi Arabia: prevalence and socio-demographic correlates--a comparison of urban and rural populations. Rural Remote Health 2011;11(1):1517.	Saudi Arabia	2009
	Bahamdan K, Mahfouz AA, Tallab T, Badawi IA, al-Amari OM. Skin diseases among adolescent boys in Abha, Saudi Arabia. Int J Dermatol 1996;35(6):405-7.	Saudi Arabia	1995-1996
	Tan H-H, Tan AWH, Barkham T, Yan X-Y, Zhu M. Community-based study of acne vulgaris in adolescents in Singapore. Br J Dermatol 2007;157(3):547-51.	Singapore	2006-2007
	Perera A, Atukorale DN, Sivayogan S, Ariyaratne VS, Karunaratne LDA. Prevalence of skin diseases in suburban Sri Lanka. Ceylon Med J 2000;45(3):123-8.	Sri Lanka	1997
	Chen G-Y, Cheng Y-W, Wang C-Y, Hsu T-J, Hsu MM-L, Yang P-T, Chen W-C. Prevalence of skin diseases among schoolchildren in Magong, Penghu, Taiwan: a community-based clinical survey. J	Taiwan	2005

© 2017 Karimkhani C et al. *JAMA Dermatology.*

	Formos Med Assoc 2008;107(1):21-9.		
	Yang Y-C, Cheng Y-W, Lai C-S, Chen W. Prevalence of childhood acne, ephelides, warts, atopic dermatitis, psoriasis, alopecia areata and keloid in Kaohsiung County, Taiwan: a community-based clinical survey. J Eur Acad Dermatol Venereol 2007;21(5):643-9.	Taiwan	2005
	Yang Y-C, Cheng Y-W, Lai C-S, Chen W. Prevalence of childhood acne, ephelides, warts, atopic dermatitis, psoriasis, alopecia areata and keloid in Kaohsiung County, Taiwan: a community-based clinical survey. J Eur Acad Dermatol Venereol 2007;21(5):643-9.	Taiwan	2004
	Gibbs, S. Skin disease and socioeconomic conditions in rural Africa: Tanzania. Int J Dermatol 1996;35(9):633-9.	Tanzania	1995
	Henderson CA. Skin disease in rural Tanzania. Int J Dermatol 1996;35(9):640-2.	Tanzania	1991
	Komba EV, Mgonda YM. The spectrum of dermatological disorders among primary school children in Dar es Salaam. BMC Public Health 2010;10(1):765.	Tanzania	2008-2010
	Satimia FT, McBride SR, Leppard B. Prevalence of skin disease in rural Tanzania and factors influencing the choice of health care, modern or traditional. Arch Dermatol 1998;134(11):1363-6.	Tanzania	1996
	Aksu AEK, Metintas S, Saracoglu ZN, Gurel G, Sabuncu I, Arikan I, Kalyoncu C. Acne: prevalence and relationship with dietary habits in Eskisehir, Turkey. J Eur Acad Dermatol Venereol 2012;26(12):1503-9.	Turkey	2010-2011
	Aktan S, Ozmen E, Sanli B. Anxiety, depression, and nature of acne vulgaris in adolescents. Int J Dermatol 2000;39(5):354-7.	Turkey	1998-2000
	Tuncel AA, Erbagci Z. Prevalence of skin diseases among male adolescent and post-adolescent boarding school students in Turkey. J Dermatol 2005;32(7):557-64.	Turkey	2004
	Unsal A, Ayranci U. Prevalence of students with symptoms of depression among high school students in a district of western Turkey: an epidemiological study. J Sch Health 2008;78(5):287-93.	Turkey	2006
	Uslu G, Sendur N, Uslu M, Savk E, Karaman G, Eskin M. Acne: prevalence, perceptions and effects on psychological health among adolescents in	Turkey	2004-2006

	Aydin, Turkey. J Eur Acad Dermatol Venereol 2008;22(4):462-9		
	Stathakis V, Kilkenny M, Marks R. Descriptive epidemiology of acne vulgaris in the community. Australas J Dermatol 1997;38(3):115-23.	United Kingdom	1987-1988
	Paek SY, Koriakos A, Saxton-Daniels S, Pandya AG. Skin diseases in rural Yucatan, Mexico. Int J Dermatol 2012;51(7):823-8.	Yucatan	2009-2010
Alopecia areata	Bechelli LM, Haddad N, Pimenta WP, Pagnano PM, Melchior E Jr, Fregnan RC, Zanin LC, Arenas A. Epidemiological survey of skin diseases in schoolchildren living in the Purus Valley (Acre State, Amazonia, Brazil). Dermatologica 1981;163(1):78-93.	Brazil	1974-1975
	Canadian Institute for Health Information (CIHI). Canada National Ambulatory Care Reporting System 2002-2003. Ottawa, Canada: Canadian Institute for Health Information (CIHI).	Canada	2002
	Canadian Institute for Health Information (CIHI). Canada National Ambulatory Care Reporting System 2006-2007. Ottawa, Canada: Canadian Institute for Health Information (CIHI).	Canada	2006
	Canadian Institute for Health Information (CIHI). Canada National Ambulatory Care Reporting System 2008-2009. Ottawa, Canada: Canadian Institute for Health Information (CIHI).	Canada	2008
	Al-Rubiay KK, Al-Rubaiy LK. Dermatoepidemiology: A Household Survey Among Two Urban Areas In Basrah City, Iraq. Internet J Dermatol 2006;4(2):10.	Iraq	2005
	Walker SL, Shah M, Hubbard VG, Pradhan HM, Ghimire M. Skin disease is common in rural Nepal: results of a point prevalence study. Br J Dermatol 2008;158(2):334-8.	Nepal	2006
	Al-Saeed WY, Al-Dawood KM, Bukhari IA, Bahnassy AA. Prevalence and pattern of skin disorders among female schoolchildren in Eastern Saudi Arabia. Saudi Med J 2006;27(2):227-34.	Saudi Arabia	2003
	Amin TT, Ali A, Kaliyadan F. Skin disorders among male primary school children in Al Hassa, Saudi Arabia: prevalence and socio-demographic correlates--a comparison of urban and rural populations. Rural Remote Health 2011;11(1):1517.	Saudi Arabia	2009
	Perera A, Atukorale DN, Sivayogan S, Ariyaratne VS, Karunaratne LDA. Prevalence of skin diseases in suburban Sri Lanka. Ceylon Med J	Sri Lanka	1997

© 2017 Karimkhani C et al. *JAMA Dermatology.*

	2000;45(3):123-8.		
	Chen G-Y, Cheng Y-W, Wang C-Y, Hsu T-J, Hsu MM-L, Yang P-T, Chen W-C. Prevalence of skin diseases among schoolchildren in Magong, Penghu, Taiwan: a community-based clinical survey. J Formos Med Assoc 2008;107(1):21-9.	Taiwan	2005
	Yang Y-C, Cheng Y-W, Lai C-S, Chen W. Prevalence of childhood acne, ephelides, warts, atopic dermatitis, psoriasis, alopecia areata and keloid in Kaohsiung County, Taiwan: a community-based clinical survey. J Eur Acad Dermatol Venereol 2007;21(5):643-9.	Taiwan	2004-2005
	Gibbs, S. Skin disease and socioeconomic conditions in rural Africa: Tanzania. Int J Dermatol 1996;35(9):633-9.	Tanzania	1995
	Komba EV, Mgonda YM. The spectrum of dermatological disorders among primary school children in Dar es Salaam. BMC Public Health 2010;10(1):765.	Tanzania	2007-2010
	Analytical Sciences, Inc., National Center for Health Statistics (NCHS), Centers for Disease Control and Prevention (CDC), US Census Bureau. United States National Ambulatory Medical Care Survey 1995. Hyattsville, United States: National Center for Health Statistics (NCHS), Centers for Disease Control and Prevention (CDC).	United States	1995-2001
	Analytical Sciences, Inc., National Center for Health Statistics (NCHS), Centers for Disease Control and Prevention (CDC), US Census Bureau. United States National Hospital Ambulatory Medical Care Survey 1995. Hyattsville, United States: National Center for Health Statistics (NCHS), Centers for Disease Control and Prevention (CDC).	United States	1995-2001
	Constella Group, National Center for Health Statistics (NCHS), Centers for Disease Control and Prevention (CDC), US Census Bureau. United States National Ambulatory Medical Care Survey 2002. Hyattsville, United States: National Center for Health Statistics (NCHS), Centers for Disease Control and Prevention (CDC).	United States	2002-2006
	Constella Group, National Center for Health Statistics (NCHS), Centers for Disease Control and Prevention (CDC), US Census Bureau. United States National Hospital Ambulatory Medical Care Survey 2002. Hyattsville, United States: National	United States	2002-2006

© 2017 Karimkhani C et al. *JAMA Dermatology.*

	Center for Health Statistics (NCHS), Centers for Disease Control and Prevention (CDC).		
	National Center for Health Statistics (NCHS), Centers for Disease Control and Prevention (CDC), SRA International, Inc., US Census Bureau. United States National Ambulatory Medical Care Survey 2007. Hyattsville, United States: National Center for Health Statistics (NCHS), Centers for Disease Control and Prevention (CDC).	United States	2007-2010
	National Center for Health Statistics (NCHS), Centers for Disease Control and Prevention (CDC), SRA International, Inc., US Census Bureau. United States National Hospital Ambulatory Medical Care Survey 2007. Hyattsville, United States: National Center for Health Statistics (NCHS), Centers for Disease Control and Prevention (CDC).	United States	2007-2010
	National Center for Health Statistics (NCHS), Centers for Disease Control and Prevention (CDC), SRA International, Inc., US Census Bureau. United States National Hospital Ambulatory Medical Care Survey 2010 and United States National Ambulatory Medical Care Survey 2010.	United States	2010
	National Center for Health Statistics (NCHS), Centers for Disease Control and Prevention (CDC), US Census Bureau. United States National Ambulatory Medical Care Survey 1995 and United States National Hospital Ambulatory Medical Care Survey 1995.	United States	1995-2009
	Safavi K. Prevalence of alopecia areata in the First National Health and Nutrition Examination Survey. Arch Dermatol 1992;128(5):702.	United States	1971-1974
Bacterial skin diseases	Ministry of Health (Mexico). Mexico Ministry of Health Hospital Discharges. Mexico City, Mexico: Ministry of Health (Mexico).	Aguascalientes	2000-2011
	Federal Ministry of Health (Austria), Statistics Austria. Austria Hospital Inpatient Discharges 1989. Vienna, Austria: Statistics Austria.	Austria	1989-2010
	Ministry of Health (Mexico). Mexico Ministry of Health Hospital Discharges. Mexico City, Mexico: Ministry of Health (Mexico).	Baja California	2000-2011
	Ministry of Health (Mexico). Mexico Ministry of Health Hospital Discharges. Mexico City, Mexico: Ministry of Health (Mexico).	Baja California Sur	2000-2011
	WHO Regional Office for Europe (EURO-WHO). European Hospital Morbidity Database. Copenhagen, Denmark: WHO Regional Office for	Belgium	2003-2009

© 2017 Karimkhani C et al. *JAMA Dermatology.*

	Europe (EURO-WHO).		
	Ministry of Health (Brazil). Brazil Hospital Information System 2006. Rio de Janeiro, Brazil: Ministry of Health (Brazil).	Brazil	2006-2009
	Ministry of Health (Mexico). Mexico Ministry of Health Hospital Discharges. Mexico City, Mexico: Ministry of Health (Mexico).	Campeche	2000-2011
	Canadian Institute for Health Information (CIHI). Canada Discharge Abstract Database. Ottawa, Canada: Canadian Institute for Health Information (CIHI).	Canada	1994-2009
	Ministry of Health (Mexico). Mexico Ministry of Health Hospital Discharges. Mexico City, Mexico: Ministry of Health (Mexico).	Chiapas	2000-2011
	Ministry of Health (Mexico). Mexico Ministry of Health Hospital Discharges. Mexico City, Mexico: Ministry of Health (Mexico).	Chihuahua	2000-2011
	Ministry of Health (Mexico). Mexico Ministry of Health Hospital Discharges. Mexico City, Mexico: Ministry of Health (Mexico).	Coahuila	2000-2011
	Ministry of Health (Mexico). Mexico Ministry of Health Hospital Discharges. Mexico City, Mexico: Ministry of Health (Mexico).	Colima	2000-2011
	WHO Regional Office for Europe (EURO-WHO). European Hospital Morbidity Database. Copenhagen, Denmark: WHO Regional Office for Europe (EURO-WHO).	Croatia	2002-2011
	WHO Regional Office for Europe (EURO-WHO). European Hospital Morbidity Database. Copenhagen, Denmark: WHO Regional Office for Europe (EURO-WHO).	Cyprus	2002-2010
	WHO Regional Office for Europe (EURO-WHO). European Hospital Morbidity Database. Copenhagen, Denmark: WHO Regional Office for Europe (EURO-WHO).	Czech Republic	2000-2010
	WHO Regional Office for Europe (EURO-WHO). European Hospital Morbidity Database. Copenhagen, Denmark: WHO Regional Office for Europe (EURO-WHO).	Denmark	2003-2006
	Ministry of Health (Mexico). Mexico Ministry of Health Hospital Discharges 2000. Mexico City, Mexico: Ministry of Health (Mexico).	Distrito Federal	2000-2011
	Ministry of Health (Mexico). Mexico Ministry of Health Hospital Discharges 2000. Mexico City, Mexico: Ministry of Health (Mexico).	Durango	2000-2011
	NHS England. United Kingdom - England Hospital	East	2002-

© 2017 Karimkhani C et al. *JAMA Dermatology.*

	Episode Statistics.	Midlands	2012
	NHS England. United Kingdom - England Hospital Episode Statistics.	East of England	2002-2012
	National Institute of Statistics and Censuses (Ecuador). Ecuador Hospital Inpatient Discharges 1997. Quito, Ecuador: National Institute of Statistics and Censuses (Ecuador).	Ecuador	1997-2011
	Abdel-Hafez K, Abdel-Aty MA, Hofny ERM. Prevalence of skin diseases in rural areas of Assiut Governorate, Upper Egypt. Int J Dermatol 2003;42(11):887-92.	Egypt	1994-1996
	Dagnew MB, Günther E. Epidemiology of communicable skin diseases in school children of a rural area in North Ethiopia. Dermatol Monatsschr 1990;176(176):219-23.	Ethiopia	1989
	Leekassa R, Bizuneh E, Alem A, Fekadu A, Shibre T. Community diagnosis of common skin diseases in the Zay community of the Zeway Islands, Ethiopia. Ethiop Med J 2005;43(3):189-95.	Ethiopia	1998
	Woldeamanuel Y, Leekassa R, Chryssanthou E, Menghistu Y, Petrini B. Prevalence of tinea capitis in Ethiopian schoolchildren. Mycoses 2005;48(2):137-41.	Ethiopia	2004
	Woldeamanuel Y, Leekassa R, Chryssanthou E, Menghistu Y, Petrini B. Prevalence of tinea capitis in Ethiopian schoolchildren. Mycoses 2005;48(2):137-41.	Ethiopia	2003-2005
	Steer AC, Jenney AWJ, Kado J, Batzloff MR, La Vincente S, Waqatakirewa L, Mulholland EK, Carapetis JR. High burden of impetigo and scabies in a tropical country. PLoS Negl Trop Dis 2009;3(6):e467.	Fiji	2006-2007
	Thomas M, Woodfield G, Moses C, Amos G. Soil-transmitted helminth infection, skin infection, anaemia, and growth retardation in schoolchildren of Taveuni Island, Fiji. N Z Med J 2005;118(1216):1492.	Fiji	2004
	WHO Regional Office for Europe (EURO-WHO). European Hospital Morbidity Database. Copenhagen, Denmark: WHO Regional Office for Europe (EURO-WHO).	Finland	2002-2009
	Kramkimel N, Soussan V, Beauchet A, Duhamel A, Saiag P, Chevallier B, Mahé E. High frequency, diversity and severity of skin diseases in a paediatric emergency department. J Eur Acad Dermatol Venereol 2010;24(12):1468-75.	France	2006

	Hogewoning A, Amoah A, Bavinck JNB, Boakye D, Yazdanbakhsh M, Adegnika A, De Smedt S, Fonteyne Y, Willemze R, Lavrijsen A. Skin diseases among schoolchildren in Ghana, Gabon, and Rwanda. Int J Dermatol 2013;52(5):589-600.	Gabon	2004-2005, 2007
	NHS England. United Kingdom - England Hospital Episode Statistics 2002-2003.	Greater London	2002-2012
	Ministry of Health (Mexico). Mexico Ministry of Health Hospital Discharges 2000. Mexico City, Mexico: Ministry of Health (Mexico).	Guanajuato	2000-2011
	Ministry of Health (Mexico). Mexico Ministry of Health Hospital Discharges 2000. Mexico City, Mexico: Ministry of Health (Mexico).	Guerrero	2000-2011
	Ministry of Health (Mexico). Mexico Ministry of Health Hospital Discharges 2000. Mexico City, Mexico: Ministry of Health (Mexico).	Hidalgo	2000-2011
	WHO Regional Office for Europe (EURO-WHO). European Hospital Morbidity Database. Copenhagen, Denmark: WHO Regional Office for Europe (EURO-WHO).	Iceland	2000-2008
	Kumar R, Vohra H, Chakraborty A, Sharma YP, Bandhopadhya S, Dhanda V, Sagar V, Sharma M, Shah B, Ganguly NK. Epidemiology of group A streptococcal pharyngitis and impetigo: a cross-sectional and follow up study in a rural community of northern India. Indian J Med Res 2009;130(6):765-771.	India	2000-2002
	Patel JK, Vyas AP, Berman B, Vierra M. Incidence of childhood dermatosis in India. Skinmed 2010;8(3):136-42.	India	2000-2002
	Al-Rubiay KK, Al-Rubaiy LK. Dermatoepidemiology: A Household Survey Among Two Urban Areas In Basrah City, Iraq. Internet J Dermatol 2006;4(2):10	Iraq	2005
	WHO Regional Office for Europe (EURO-WHO). European Hospital Morbidity Database. Copenhagen, Denmark: WHO Regional Office for Europe (EURO-WHO).	Israel	1999-2009
	WHO Regional Office for Europe (EURO-WHO). European Hospital Morbidity Database. Copenhagen, Denmark: WHO Regional Office for Europe (EURO-WHO).	Italy	2002-2006, 2010
	Ministry of Health (Mexico). Mexico Ministry of Health Hospital Discharges 2000. Mexico City, Mexico: Ministry of Health (Mexico).	Jalisco	2000-2011
	Schmeller W, Dzikus A. Skin diseases in children	Kenya	1993,

© 2017 Karimkhani C et al. *JAMA Dermatology.*

	in rural Kenya: long term results of a dermatology project within the primary health care system. Br J Dermatol 2001;144(1):118-124.		1999
	WHO Regional Office for Europe (EURO-WHO). European Hospital Morbidity Database. Copenhagen, Denmark: WHO Regional Office for Europe (EURO-WHO).	Latvia	2004-2011
	WHO Regional Office for Europe (EURO-WHO). European Hospital Morbidity Database. Copenhagen, Denmark: WHO Regional Office for Europe (EURO-WHO).	Lithuania	2001-2010
	WHO Regional Office for Europe (EURO-WHO). European Hospital Morbidity Database. Copenhagen, Denmark: WHO Regional Office for Europe (EURO-WHO).	Luxembourg	2002-2010
	WHO Regional Office for Europe (EURO-WHO). European Hospital Morbidity Database. Copenhagen, Denmark: WHO Regional Office for Europe (EURO-WHO).	Malta	2009-2011
	Ministry of Health (Mexico). Mexico Ministry of Health Hospital Discharges. Mexico City, Mexico: Ministry of Health (Mexico).	Mexico	2000-2011
	Ministry of Health (Mexico). Mexico Ministry of Health Hospital Discharges. Mexico City, Mexico: Ministry of Health (Mexico).	Michoacan de Ocampo	2000-2011
	Ministry of Health (Mexico). Mexico Ministry of Health Hospital Discharges. Mexico City, Mexico: Ministry of Health (Mexico).	Morelos	2000-2011
	Ministry of Health (Mexico). Mexico Ministry of Health Hospital Discharges. Mexico City, Mexico: Ministry of Health (Mexico).	Nayarit	2000-2011
	Oyedeji O, Okeniyi J, Ogunlesi T, Onayemi O, Oyedeji G, Oyelami O. Parental factors influencing the prevalence of skin infections and infestations among Nigerian primary school pupils. Internet J Dermatol 2006;3(2):6.	Nigeria	2003
	NHS England. United Kingdom - England Hospital Episode Statistics.	North East England	2002-2012
	NHS England. United Kingdom - England Hospital Episode Statistics.	North West England	2002-2012
	RÀrtveit S, Skutlaberg DH, Langeland N, Rortveit G. Impetigo in a population over 8.5 years: incidence, fusidic acid resistance and molecular characteristics. J Antimicrob Chemother 2011;66(6):1360-4.	Norway	2001-2009
	WHO Regional Office for Europe (EURO-WHO).	Norway	2002-

	European Hospital Morbidity Database. Copenhagen, Denmark: WHO Regional Office for Europe (EURO-WHO).		2011
	Ministry of Health (Mexico). Mexico Ministry of Health Hospital Discharges. Mexico City, Mexico: Ministry of Health (Mexico).	Nuevo Leon	2000-2011
	Ministry of Health (Mexico). Mexico Ministry of Health Hospital Discharges. Mexico City, Mexico: Ministry of Health (Mexico).	Oaxaca	2000-2011
	Luby SP, Agboatwalla M, Feikin DR, Painter J, Billhimer W, Altaf A, Hoekstra, RM. Effect of handwashing on child health: a randomised controlled trial. Lancet 2005;366(9481):225-233.	Pakistan	2002-2003
	Gutierrez EL, Galarza C, Ramos W. Prevalencia de Enfermedades Dermatológicas en una comunidad rural de Ucayali, Perú. Dermatol Peru 2009;19(2):104-13.	Peru	2005
	WHO Regional Office for Europe (EURO-WHO). European Hospital Morbidity Database. Copenhagen, Denmark: WHO Regional Office for Europe (EURO-WHO).	Poland	2003-2010
	Massa A, Alves R, Amado J, Matos E, Sanches M, Selores M, Santos C, Costa V, Velho G, Oliveira M, Ferreira E, Taveira M, Silva NS, Granado E, Lemos A, Calheiros JM. Prevalence of cutaneous lesions in Freixo de Espada à Cinta. Acta Med Port 2000;13(5-6):247-54.	Portugal	1998
	WHO Regional Office for Europe (EURO-WHO). European Hospital Morbidity Database. Copenhagen, Denmark: WHO Regional Office for Europe (EURO-WHO).	Portugal	2004-2008
	Ministry of Health (Mexico). Mexico Ministry of Health Hospital Discharges. Mexico City, Mexico: Ministry of Health (Mexico).	Puebla	2000-2011
	Ministry of Health (Mexico). Mexico Ministry of Health Hospital Discharges. Mexico City, Mexico: Ministry of Health (Mexico).	Queretaro	2000-2011
	Ministry of Health (Mexico). Mexico Ministry of Health Hospital Discharges. Mexico City, Mexico: Ministry of Health (Mexico).	Quintana Roo	2000-2011
	Hogewoning A, Amoah A, Bavinck JNB, Boakye D, Yazdanbakhsh M, Adegnika A, De Smedt S, Fonteyne Y, Willemze R, Lavrijsen A. Skin diseases among schoolchildren in Ghana, Gabon, and Rwanda. Int J Dermatol 2013;52(5):589-600.	Rwanda	2007
	Ministry of Health (Mexico). Mexico Ministry of	San Luis	2000-

© 2017 Karimkhani C et al. *JAMA Dermatology.*

	Health Hospital Discharges. Mexico City, Mexico: Ministry of Health (Mexico).	Potosi	2011
	Amin TT, Ali A, Kaliyadan F. Skin disorders among male primary school children in Al Hassa, Saudi Arabia: prevalence and socio-demographic correlates--a comparison of urban and rural populations. Rural Remote Health 2011;11(1):1517.	Saudi Arabia	2009
	Ministry of Health (Mexico). Mexico Ministry of Health Hospital Discharges. Mexico City, Mexico: Ministry of Health (Mexico).	Sinaloa	2000-2011
	WHO Regional Office for Europe (EURO-WHO). European Hospital Morbidity Database. Copenhagen, Denmark: WHO Regional Office for Europe (EURO-WHO).	Slovakia	2002-2010
	WHO Regional Office for Europe (EURO-WHO). European Hospital Morbidity Database. Copenhagen, Denmark: WHO Regional Office for Europe (EURO-WHO).	Slovenia	2004-2009
	Ministry of Health (Mexico). Mexico Ministry of Health Hospital Discharges. Mexico City, Mexico: Ministry of Health (Mexico).	Sonora	2000-2011
	NHS England. United Kingdom - England Hospital Episode Statistics.	South East England	2002-2012
	NHS England. United Kingdom - England Hospital Episode Statistics.	South West England	2002-2012
	WHO Regional Office for Europe (EURO-WHO). European Hospital Morbidity Database. Copenhagen, Denmark: WHO Regional Office for Europe (EURO-WHO).	Spain	2000-2010
	WHO Regional Office for Europe (EURO-WHO). European Hospital Morbidity Database. Copenhagen, Denmark: WHO Regional Office for Europe (EURO-WHO).	Switzerland	2002-2010
	Ministry of Health (Mexico). Mexico Ministry of Health Hospital Discharges. Mexico City, Mexico: Ministry of Health (Mexico).	Tabasco	2000-2011
	Ministry of Health (Mexico). Mexico Ministry of Health Hospital Discharges. Mexico City, Mexico: Ministry of Health (Mexico).	Tamaulipas	2000-2011
	Ministry of Health (Mexico). Mexico Ministry of Health Hospital Discharges. Mexico City, Mexico: Ministry of Health (Mexico).	Tlaxcala	2000-2011
	WHO Regional Office for Europe (EURO-WHO). European Hospital Morbidity Database. Copenhagen, Denmark: WHO Regional Office for Europe (EURO-WHO).	United Kingdom	2000-2010

	National Center for Health Statistics (NCHS), Centers for Disease Control and Prevention (CDC), US Census Bureau. United States National Hospital Discharge Survey 1979. Hyattsville, United States: National Center for Health Statistics (NCHS), Centers for Disease Control and Prevention (CDC).	United States	1979-2010
	Ministry of Health (Mexico). Mexico Ministry of Health Hospital Discharges. Mexico City, Mexico: Ministry of Health (Mexico).	Veracruz de Ignacio de la Llava	2000-2011
	NHS England. United Kingdom - England Hospital Episode Statistics.	West Midlands	2002-2012
	NHS England. United Kingdom - England Hospital Episode Statistics.	Yorkshire and the Humber	2002-2012
	Ministry of Health (Mexico). Mexico Ministry of Health Hospital Discharges. Mexico City, Mexico: Ministry of Health (Mexico).	Yucatan	2000-2011
	Ministry of Health (Mexico). Mexico Ministry of Health Hospital Discharges. Mexico City, Mexico: Ministry of Health (Mexico).	Zacatecas	2000-2011
Cellulitis	Federal Ministry of Health (Austria), Statistics Austria. Austria Hospital Inpatient Discharges. Vienna, Austria: Statistics Austria.	Austria	1989-2010
	WHO Regional Office for Europe (EURO-WHO). European Hospital Morbidity Database. Copenhagen, Denmark: WHO Regional Office for Europe (EURO-WHO).	Belgium	2003-2009
	Ministry of Health (Brazil). Brazil Hospital Information System 2006. Rio de Janeiro, Brazil: Ministry of Health (Brazil).	Brazil	2006-2009
	Canadian Institute for Health Information (CIHI). Canada Discharge Abstract Database. Ottawa, Canada: Canadian Institute for Health Information (CIHI).	Canada	1994-2009
	Canadian Institute for Health Information (CIHI). Canada Discharge Abstract Database 2002 and Canada National Ambulatory Care Reporting System.	Canada	2002-2009
	WHO Regional Office for Europe (EURO-WHO). European Hospital Morbidity Database. Copenhagen, Denmark: WHO Regional Office for Europe (EURO-WHO).	Croatia	2002-2011
	WHO Regional Office for Europe (EURO-WHO). European Hospital Morbidity Database. Copenhagen, Denmark: WHO Regional Office for Europe (EURO-WHO).	Cyprus	2002-2010

© 2017 Karimkhani C et al. *JAMA Dermatology.*

	WHO Regional Office for Europe (EURO-WHO). European Hospital Morbidity Database. Copenhagen, Denmark: WHO Regional Office for Europe (EURO-WHO).	Czech Republic	2000-2010
	WHO Regional Office for Europe (EURO-WHO). European Hospital Morbidity Database. Copenhagen, Denmark: WHO Regional Office for Europe (EURO-WHO).	Denmark	2003-2006
	NHS England. United Kingdom - England Hospital Episode Statistics.	East Midlands	2002-2012
	NHS England. United Kingdom - England Hospital Episode Statistics.	East of England	2002-2012
	National Institute of Statistics and Censuses (Ecuador). Ecuador Hospital Inpatient Discharges. Quito, Ecuador: National Institute of Statistics and Censuses (Ecuador).	Ecuador	1997-2010
	WHO Regional Office for Europe (EURO-WHO). European Hospital Morbidity Database. Copenhagen, Denmark: WHO Regional Office for Europe (EURO-WHO).	Finland	2002-2010
	NHS England. United Kingdom - England Hospital Episode Statistics.	Greater London	2002-2012
	WHO Regional Office for Europe (EURO-WHO). European Hospital Morbidity Database. Copenhagen, Denmark: WHO Regional Office for Europe (EURO-WHO).	Iceland	2000-2009
	WHO Regional Office for Europe (EURO-WHO). European Hospital Morbidity Database. Copenhagen, Denmark: WHO Regional Office for Europe (EURO-WHO).	Israel	1999-2009
	WHO Regional Office for Europe (EURO-WHO). European Hospital Morbidity Database. Copenhagen, Denmark: WHO Regional Office for Europe (EURO-WHO).	Italy	2002-2006, 2010
	WHO Regional Office for Europe (EURO-WHO). European Hospital Morbidity Database. Copenhagen, Denmark: WHO Regional Office for Europe (EURO-WHO).	Latvia	2004-2011
	WHO Regional Office for Europe (EURO-WHO). European Hospital Morbidity Database. Copenhagen, Denmark: WHO Regional Office for Europe (EURO-WHO).	Lithuania	2001-2010
	WHO Regional Office for Europe (EURO-WHO). European Hospital Morbidity Database. Copenhagen, Denmark: WHO Regional Office for Europe (EURO-WHO).	Luxemboug	2002-2010

© 2017 Karimkhani C et al. *JAMA Dermatology.*

	WHO Regional Office for Europe (EURO-WHO). European Hospital Morbidity Database. Copenhagen, Denmark: WHO Regional Office for Europe (EURO-WHO).	Malta	2009-2011
	Ministry of Health (Mexico). Mexico Ministry of Health Hospital Discharges. Mexico City, Mexico: Ministry of Health (Mexico)	Mexico	2000-2011
	NHS England. United Kingdom - England Hospital Episode Statistics.	North East England	2002-2012
	NHS England. United Kingdom - England Hospital Episode Statistics.	North West England	2002-2012
	WHO Regional Office for Europe (EURO-WHO). European Hospital Morbidity Database. Copenhagen, Denmark: WHO Regional Office for Europe (EURO-WHO).	Norway	2002-2011
	WHO Regional Office for Europe (EURO-WHO). European Hospital Morbidity Database. Copenhagen, Denmark: WHO Regional Office for Europe (EURO-WHO).	Poland	2003-2010
	WHO Regional Office for Europe (EURO-WHO). European Hospital Morbidity Database. Copenhagen, Denmark: WHO Regional Office for Europe (EURO-WHO).	Portugal	2004-2008
	WHO Regional Office for Europe (EURO-WHO). European Hospital Morbidity Database. Copenhagen, Denmark: WHO Regional Office for Europe (EURO-WHO).	Slovakia	2002-2010
	WHO Regional Office for Europe (EURO-WHO). European Hospital Morbidity Database. Copenhagen, Denmark: WHO Regional Office for Europe (EURO-WHO).	Slovenia	2004-2009
	NHS England. United Kingdom - England Hospital Episode Statistics.	South East England	2002-2012
	NHS England. United Kingdom - England Hospital Episode Statistics.	South West England	2002-2012
	WHO Regional Office for Europe (EURO-WHO). European Hospital Morbidity Database. Copenhagen, Denmark: WHO Regional Office for Europe (EURO-WHO).	Spain	2000-2005
	WHO Regional Office for Europe (EURO-WHO). European Hospital Morbidity Database. Copenhagen, Denmark: WHO Regional Office for Europe (EURO-WHO).	Sweden	2010
	WHO Regional Office for Europe (EURO-WHO). European Hospital Morbidity Database. Copenhagen, Denmark: WHO Regional Office for	Switzerland	2002-2010

© 2017 Karimkhani C et al. *JAMA Dermatology.*

	Europe (EURO-WHO).		
	WHO Regional Office for Europe (EURO-WHO). European Hospital Morbidity Database. Copenhagen, Denmark: WHO Regional Office for Europe (EURO-WHO).	United Kingdom	2000-2010
	Analytical Sciences, Inc., National Center for Health Statistics (NCHS), Centers for Disease Control and Prevention (CDC), US Census Bureau. United States National Ambulatory Medical Care Survey 1996. Hyattsville, United States: National Center for Health Statistics (NCHS), Centers for Disease Control and Prevention (CDC). National Center for Health Statistics (NCHS), Centers for Disease Control and Prevention (CDC), US Census Bureau. United States National Hospital Discharge Survey 1996. Hyattsville, United States: National Center for Health Statistics (NCHS), Centers for Disease Control and Prevention (CDC).	United States	1996
	Constella Group, National Center for Health Statistics (NCHS), Centers for Disease Control and Prevention (CDC), US Census Bureau. United States National Hospital Ambulatory Medical Care Survey 2002. National Center for Health Statistics (NCHS), Centers for Disease Control and Prevention (CDC), US Census Bureau. United States National Hospital Discharge Survey 2002. Hyattsville, United States: National Center for Health Statistics (NCHS), Centers for Disease Control and Prevention (CDC).	United States	2002
	National Center for Health Statistics (NCHS), Centers for Disease Control and Prevention (CDC), US Census Bureau. United States National Hospital Ambulatory Medical Care Survey 2003. National Center for Health Statistics (NCHS), Centers for Disease Control and Prevention (CDC), US Census Bureau. United States National Hospital Discharge Survey 2001. Hyattsville, United States: National Center for Health Statistics (NCHS), Centers for Disease Control and Prevention (CDC).	United States	2001
	Agency for Healthcare Research and Quality. United States Medical Expenditure Panel Survey 1996-2011. Agency for Healthcare Research and Quality.	United States	1996-2011
	Analytical Sciences, Inc., National Center for Health Statistics (NCHS), Centers for Disease Control and Prevention (CDC), US Census Bureau.	United States	1995, 1997-1998,

© 2017 Karimkhani C et al. *JAMA Dermatology.*

	United States National Ambulatory Medical Care Survey. Hyattsville, United States: National Center for Health Statistics (NCHS), Centers for Disease Control and Prevention (CDC). National Center for Health Statistics (NCHS), Centers for Disease Control and Prevention (CDC), US Census Bureau. United States National Hospital Discharge Survey. Hyattsville, United States: National Center for Health Statistics (NCHS), Centers for Disease Control and Prevention (CDC).		2000-2001
	Analytical Sciences, Inc., National Center for Health Statistics (NCHS), Centers for Disease Control and Prevention (CDC), US Census Bureau. United States National Hospital Ambulatory Medical Care Survey 1995. Analytical Sciences, Inc., National Center for Health Statistics (NCHS), Centers for Disease Control and Prevention (CDC), US Census Bureau. United States National Ambulatory Medical Care Survey 1995. Hyattsville, United States: National Center for Health Statistics (NCHS), Centers for Disease Control and Prevention (CDC). National Center for Health Statistics (NCHS), Centers for Disease Control and Prevention (CDC), US Census Bureau. United States National Hospital Discharge Survey 1995. Hyattsville, United States: National Center for Health Statistics (NCHS), Centers for Disease Control and Prevention (CDC).	United States	1995-2001
	Analytical Sciences, Inc., National Center for Health Statistics (NCHS), Centers for Disease Control and Prevention (CDC), US Census Bureau. United States National Hospital Ambulatory Medical Care Survey 1995. National Center for Health Statistics (NCHS), Centers for Disease Control and Prevention (CDC), US Census Bureau. United States National Hospital Discharge Survey 1995. Hyattsville, United States: National Center for Health Statistics (NCHS), Centers for Disease Control and Prevention (CDC).	United States	1995-2000
	Constella Group, National Center for Health Statistics (NCHS), Centers for Disease Control and Prevention (CDC), US Census Bureau. United States National Ambulatory Medical Care Survey 2002. Hyattsville, United States: National Center for Health Statistics (NCHS), Centers for Disease Control and Prevention (CDC). Constella Group,	United States	2002, 2004-2006

© 2017 Karimkhani C et al. *JAMA Dermatology.*

	National Center for Health Statistics (NCHS), Centers for Disease Control and Prevention (CDC), US Census Bureau. United States National Hospital Ambulatory Medical Care Survey 2002. National Center for Health Statistics (NCHS), Centers for Disease Control and Prevention (CDC), US Census Bureau. United States National Hospital Discharge Survey 2002. Hyattsville, United States: National Center for Health Statistics (NCHS), Centers for Disease Control and Prevention (CDC).		
	Constella Group, National Center for Health Statistics (NCHS), Centers for Disease Control and Prevention (CDC), US Census Bureau. United States National Ambulatory Medical Care Survey 2003. Hyattsville, United States: National Center for Health Statistics (NCHS), Centers for Disease Control and Prevention (CDC). National Center for Health Statistics (NCHS), Centers for Disease Control and Prevention (CDC), US Census Bureau. United States National Hospital Discharge Survey 2003. Hyattsville, United States: National Center for Health Statistics (NCHS), Centers for Disease Control and Prevention (CDC).	United States	2004-2006
	Ellis Simonsen SM, van Orman ER, Hatch BE, Jones SS, Gren LH, Hegmann KT, Lyon JL. Cellulitis incidence in a defined population. Epidemiol Infect. 2006; 134(2): 293-299.	United States	1997-2002
	National Center for Health Statistics (NCHS), Centers for Disease Control and Prevention (CDC), SRA International, Inc., US Census Bureau. United States National Ambulatory Medical Care Survey. Hyattsville, United States: National Center for Health Statistics (NCHS), Centers for Disease Control and Prevention (CDC). National Center for Health Statistics (NCHS), Centers for Disease Control and Prevention (CDC), US Census Bureau. United States National Hospital Discharge Survey.	United States	2008-2009
	National Center for Health Statistics (NCHS), Centers for Disease Control and Prevention (CDC), SRA International, Inc., US Census Bureau. United States National Hospital Ambulatory Medical Care Survey 2007. National Center for Health Statistics (NCHS), Centers for Disease Control and Prevention (CDC), SRA International, Inc., US Census Bureau. United States National Ambulatory Medical Care Survey 2007. Hyattsville, United	United States	2007-2009

© 2017 Karimkhani C et al. *JAMA Dermatology.*

	States: National Center for Health Statistics (NCHS), Centers for Disease Control and Prevention (CDC). National Center for Health Statistics (NCHS), Centers for Disease Control and Prevention (CDC), US Census Bureau. United States National Hospital Discharge Survey 2007.		
	National Center for Health Statistics (NCHS), Centers for Disease Control and Prevention (CDC), SRA International, Inc., US Census Bureau. United States National Hospital Ambulatory Medical Care Survey. National Center for Health Statistics (NCHS), Centers for Disease Control and Prevention (CDC), US Census Bureau. United States National Hospital Discharge Survey.	United States	2007-2009
	National Center for Health Statistics (NCHS), Centers for Disease Control and Prevention (CDC), US Census Bureau. United States National Hospital Ambulatory Medical Care Survey 2003. Constella Group, National Center for Health Statistics (NCHS), Centers for Disease Control and Prevention (CDC), US Census Bureau. United States National Ambulatory Medical Care Survey 2003. Hyattsville, United States: National Center for Health Statistics (NCHS), Centers for Disease Control and Prevention (CDC). National Center for Health Statistics (NCHS), Centers for Disease Control and Prevention (CDC), US Census Bureau. United States National Hospital Discharge Survey 2003. Hyattsville, United States: National Center for Health Statistics (NCHS), Centers for Disease Control and Prevention (CDC).	United States	2003
	National Center for Health Statistics (NCHS), Centers for Disease Control and Prevention (CDC), US Census Bureau. United States National Hospital Ambulatory Medical Care Survey 2003. National Center for Health Statistics (NCHS), Centers for Disease Control and Prevention (CDC), US Census Bureau. United States National Hospital Discharge Survey 2003. Hyattsville, United States: National Center for Health Statistics (NCHS), Centers for Disease Control and Prevention (CDC).	United States	2003
	National Center for Health Statistics (NCHS), Centers for Disease Control and Prevention (CDC), US Census Bureau. United States National Hospital Discharge Survey 1979. Hyattsville, United States: National Center for Health Statistics (NCHS),	United States	1979-2004, 2007-2009

© 2017 Karimkhani C et al. *JAMA Dermatology.*

	Centers for Disease Control and Prevention (CDC).		
	National Center for Health Statistics (NCHS), Centers for Disease Control and Prevention (CDC), US Census Bureau. United States National Hospital Discharge Survey 2010. National Center for Health Statistics (NCHS), Centers for Disease Control and Prevention (CDC), SRA International, Inc., US Census Bureau. United States National Hospital Ambulatory Medical Care Survey 2010. Hyattsville, United States: National Center for Health Statistics (NCHS), Centers for Disease Control and Prevention (CDC). National Center for Health Statistics (NCHS), Centers for Disease Control and Prevention (CDC), SRA International, Inc., US Census Bureau. United States National Ambulatory Medical Care Survey 2010. Hyattsville, United States: National Center for Health Statistics (NCHS), Centers for Disease Control and Prevention (CDC).	United States	2010
	National Center for Health Statistics (NCHS), Centers for Disease Control and Prevention (CDC), US Census Bureau. United States National Hospital Discharge Survey 2010. National Center for Health Statistics (NCHS), Centers for Disease Control and Prevention (CDC), SRA International, Inc., US Census Bureau. United States National Ambulatory Medical Care Survey 2010. Hyattsville, United States: National Center for Health Statistics (NCHS), Centers for Disease Control and Prevention (CDC).	United States	2010
	National Center for Health Statistics (NCHS), Centers for Disease Control and Prevention (CDC). United States National Ambulatory Medical Care Survey 2002 and United States National Hospital Discharge Survey 2002.	United States	2002
	NHS England. United Kingdom - England Hospital Episode Statistics.	West Midlands	2002-2012
	NHS England. United Kingdom - England Hospital Episode Statistics.	Yorkshire and the Humber	2002-2012
Decubitus ulcer	Ministry of Health (Mexico). Mexico Ministry of Health Hospital Discharges. Mexico City, Mexico: Ministry of Health (Mexico).	Aguascalientes	2000-2011
	Federal Ministry of Health (Austria), Statistics Austria. Austria Hospital Inpatient Discharges. Vienna, Austria: Statistics Austria.	Austria	1989-2010

	Ministry of Health (Mexico). Mexico Ministry of Health Hospital Discharges. Mexico City, Mexico: Ministry of Health (Mexico).	Baja California	2000-2011
	Ministry of Health (Mexico). Mexico Ministry of Health Hospital Discharges. Mexico City, Mexico: Ministry of Health (Mexico).	Baja California Sur	2000-2011
	WHO Regional Office for Europe (EURO-WHO). European Hospital Morbidity Database. Copenhagen, Denmark: WHO Regional Office for Europe (EURO-WHO).	Belgium	2003-2009
	Ministry of Health (Brazil). Brazil Hospital Information System. Rio de Janeiro, Brazil: Ministry of Health (Brazil).	Brazil	2006-2009
	Ministry of Health (Mexico). Mexico Ministry of Health Hospital Discharges 2000. Mexico City, Mexico: Ministry of Health (Mexico).	Campeche	2000-2011
	Canadian Institute for Health Information (CIHI). Canada Discharge Abstract Database. Ottawa, Canada: Canadian Institute for Health Information (CIHI).	Canada	2002-2009
	Ministry of Health (Mexico). Mexico Ministry of Health Hospital Discharges. Mexico City, Mexico: Ministry of Health (Mexico).	Chiapas	2000-2011
	Ministry of Health (Mexico). Mexico Ministry of Health Hospital Discharges Mexico City, Mexico: Ministry of Health (Mexico).	Chihuahua	2000-2011
	Ministry of Health (Mexico). Mexico Ministry of Health Hospital Discharges Mexico City, Mexico: Ministry of Health (Mexico).	Coahuila	2000-2011
	Ministry of Health (Mexico). Mexico Ministry of Health Hospital Discharges. Mexico City, Mexico: Ministry of Health (Mexico).	Colima	2000-2011
	WHO Regional Office for Europe (EURO-WHO). European Hospital Morbidity Database. Copenhagen, Denmark: WHO Regional Office for Europe (EURO-WHO).	Croatia	2002-2011
	WHO Regional Office for Europe (EURO-WHO). European Hospital Morbidity Database. Copenhagen, Denmark: WHO Regional Office for Europe (EURO-WHO).	Cyprus	2002-2010
	WHO Regional Office for Europe (EURO-WHO). European Hospital Morbidity Database. Copenhagen, Denmark: WHO Regional Office for Europe (EURO-WHO).	Czech Republic	2000-2010
	WHO Regional Office for Europe (EURO-WHO). European Hospital Morbidity Database.	Denmark	2003-2006

© 2017 Karimkhani C et al. *JAMA Dermatology.*

	Copenhagen, Denmark: WHO Regional Office for Europe (EURO-WHO).		
	Ministry of Health (Mexico). Mexico Ministry of Health Hospital Discharges Mexico City, Mexico: Ministry of Health (Mexico).	Distrito Federal	2000-2011
	Ministry of Health (Mexico). Mexico Ministry of Health Hospital Discharges. Mexico City, Mexico: Ministry of Health (Mexico).	Durango	2000-2011
	NHS England. United Kingdom - England Hospital Episode Statistics.	East Midlands	2002-2012
	NHS England. United Kingdom - England Hospital Episode Statistics.	East of England	2002-2012
	National Institute of Statistics and Censuses (Ecuador). Ecuador Hospital Inpatient Discharges. Quito, Ecuador: National Institute of Statistics and Censuses (Ecuador).	Ecuador	1997-2011
	WHO Regional Office for Europe (EURO-WHO). European Hospital Morbidity Database. Copenhagen, Denmark: WHO Regional Office for Europe (EURO-WHO)	Finland	2002-2010
	NHS England. United Kingdom - England Hospital Episode Statistics.	Greater London	2002-2012
	Ministry of Health (Mexico). Mexico Ministry of Health Hospital Discharges Mexico City, Mexico: Ministry of Health (Mexico).	Guanajuato	2000-2011
	Ministry of Health (Mexico). Mexico Ministry of Health Hospital Discharges. Mexico City, Mexico: Ministry of Health (Mexico).	Guerrero	2000-2011
	Ministry of Health (Mexico). Mexico Ministry of Health Hospital Discharges. Mexico City, Mexico: Ministry of Health (Mexico).	Hidalgo	2000-2011
	WHO Regional Office for Europe (EURO-WHO). European Hospital Morbidity Database. Copenhagen, Denmark: WHO Regional Office for Europe (EURO-WHO).	Iceland	2000-2009
	WHO Regional Office for Europe (EURO-WHO). European Hospital Morbidity Database. Copenhagen, Denmark: WHO Regional Office for Europe (EURO-WHO).	Israel	1999-2009
	WHO Regional Office for Europe (EURO-WHO). European Hospital Morbidity Database. Copenhagen, Denmark: WHO Regional Office for Europe (EURO-WHO).	Italy	2002-2006, 2010
	Ministry of Health (Mexico). Mexico Ministry of Health Hospital Discharges. Mexico City, Mexico: Ministry of Health (Mexico).	Jalisco	2000-2011

	WHO Regional Office for Europe (EURO-WHO). European Hospital Morbidity Database. Copenhagen, Denmark: WHO Regional Office for Europe (EURO-WHO).	Latvia	2004-2011
	WHO Regional Office for Europe (EURO-WHO). European Hospital Morbidity Database. Copenhagen, Denmark: WHO Regional Office for Europe (EURO-WHO).	Lithuania	2001-2010
	WHO Regional Office for Europe (EURO-WHO). European Hospital Morbidity Database. Copenhagen, Denmark: WHO Regional Office for Europe (EURO-WHO).	Luxembourg	2002-2010
	WHO Regional Office for Europe (EURO-WHO). European Hospital Morbidity Database. Copenhagen, Denmark: WHO Regional Office for Europe (EURO-WHO).	Malta	2009-2011
	Ministry of Health (Mexico). Mexico Ministry of Health Hospital Discharges. Mexico City, Mexico: Ministry of Health (Mexico).	Mexico	2000-2011
	Ministry of Health (Mexico). Mexico Ministry of Health Hospital Discharges. Mexico City, Mexico: Ministry of Health (Mexico).	Michoacan de Ocampo	2000-2011
	Ministry of Health (Mexico). Mexico Ministry of Health Hospital Discharges. Mexico City, Mexico: Ministry of Health (Mexico).	Morelos	2000-2011
	Ministry of Health (Mexico). Mexico Ministry of Health Hospital Discharges. Mexico City, Mexico: Ministry of Health (Mexico).	Nayarit	2000-2011
	NHS England. United Kingdom - England Hospital Episode Statistics.	North East England	2002-2012
	NHS England. United Kingdom - England Hospital Episode Statistics.	North West England	2002-2012
	Norwegian Directorate of Health. Norway Patient Register.	Norway	2008-2012
	WHO Regional Office for Europe (EURO-WHO). European Hospital Morbidity Database. Copenhagen, Denmark: WHO Regional Office for Europe (EURO-WHO).	Norway	2002-2007
	Ministry of Health (Mexico). Mexico Ministry of Health Hospital Discharges. Mexico City, Mexico: Ministry of Health (Mexico).	Nuevo Leon	2000-2011
	Ministry of Health (Mexico). Mexico Ministry of Health Hospital Discharges. Mexico City, Mexico: Ministry of Health (Mexico).	Oaxaca	2000-2011
	WHO Regional Office for Europe (EURO-WHO). European Hospital Morbidity Database.	Poland	2003-2010

© 2017 Karimkhani C et al. *JAMA Dermatology.*

	Copenhagen, Denmark: WHO Regional Office for Europe (EURO-WHO).		
	WHO Regional Office for Europe (EURO-WHO). European Hospital Morbidity Database. Copenhagen, Denmark: WHO Regional Office for Europe (EURO-WHO).	Portugal	2004-2008
	Ministry of Health (Mexico). Mexico Ministry of Health Hospital Discharges. Mexico City, Mexico: Ministry of Health (Mexico).	Puebla	2000-2011
	Ministry of Health (Mexico). Mexico Ministry of Health Hospital Discharges. Mexico City, Mexico: Ministry of Health (Mexico).	Queretaro	2000-2011
	Ministry of Health (Mexico). Mexico Ministry of Health Hospital Discharges. Mexico City, Mexico: Ministry of Health (Mexico).	Quintana Roo	2000-2011
	Ministry of Health (Mexico). Mexico Ministry of Health Hospital Discharges. Mexico City, Mexico: Ministry of Health (Mexico).	San Luis Potosi	2000-2011
	Ministry of Health (Mexico). Mexico Ministry of Health Hospital Discharges. Mexico City, Mexico: Ministry of Health (Mexico).	Sinaloa	2000-2011
	WHO Regional Office for Europe (EURO-WHO). European Hospital Morbidity Database. Copenhagen, Denmark: WHO Regional Office for Europe (EURO-WHO).	Slovakia	2002-2010
	WHO Regional Office for Europe (EURO-WHO). European Hospital Morbidity Database. Copenhagen, Denmark: WHO Regional Office for Europe (EURO-WHO).	Slovenia	2004-2009
	Ministry of Health (Mexico). Mexico Ministry of Health Hospital Discharges. Mexico City, Mexico: Ministry of Health (Mexico).	Sonora	2000-2011
	NHS England. United Kingdom - England Hospital Episode Statistics.	South East England	2002-2012
	NHS England. United Kingdom - England Hospital Episode Statistics.	South West England	2002-2012
	WHO Regional Office for Europe (EURO-WHO). European Hospital Morbidity Database. Copenhagen, Denmark: WHO Regional Office for Europe (EURO-WHO).	Spain	2000-2005
	WHO Regional Office for Europe (EURO-WHO). European Hospital Morbidity Database. Copenhagen, Denmark: WHO Regional Office for Europe (EURO-WHO).	Sweden	2010
	WHO Regional Office for Europe (EURO-WHO). European Hospital Morbidity Database.	Switzerland	2002-2010

© 2017 Karimkhani C et al. *JAMA Dermatology.*

	Copenhagen, Denmark: WHO Regional Office for Europe (EURO-WHO).		
	Ministry of Health (Mexico). Mexico Ministry of Health Hospital Discharges. Mexico City, Mexico: Ministry of Health (Mexico).	Tabasco	2000-2011
	Ministry of Health (Mexico). Mexico Ministry of Health Hospital Discharges. Mexico City, Mexico: Ministry of Health (Mexico).	Tamaulipas	2000-2011
	Ministry of Health (Mexico). Mexico Ministry of Health Hospital Discharges. Mexico City, Mexico: Ministry of Health (Mexico).	Tlaxcala	2000-2011
	Margolis DJ, Bilker W, Knauss J, Baumgarten M, Strom BL. The incidence and prevalence of pressure ulcers among elderly patients in general medical practice. Ann Epidemiol. 2002; 12(5): 321-5.	United Kingdom	1989-1996
	WHO Regional Office for Europe (EURO-WHO). European Hospital Morbidity Database. Copenhagen, Denmark: WHO Regional Office for Europe (EURO-WHO).	United Kingdom	2000-2010
	Agency for Healthcare Research and Quality. United States Medical Expenditure Panel Survey 1996-2011. Agency for Healthcare Research and Quality.	United States	1996-2011
	National Center for Health Statistics (NCHS), Centers for Disease Control and Prevention (CDC), US Census Bureau. United States National Hospital Discharge Survey. Hyattsville, United States: National Center for Health Statistics (NCHS), Centers for Disease Control and Prevention (CDC).	United States	1979-2010
	Ministry of Health (Mexico). Mexico Ministry of Health Hospital Discharges. Mexico City, Mexico: Ministry of Health (Mexico).	Veracruz de Ignacio de la Llave	2000-2011
	NHS England. United Kingdom - England Hospital Episode Statistics.	West Midlands	2002-2012
	NHS England. United Kingdom - England Hospital Episode Statistics.	Yorkshire and the Humber	2002-2012
	Ministry of Health (Mexico). Mexico Ministry of Health Hospital Discharges. Mexico City, Mexico: Ministry of Health (Mexico).	Yucatan	2000-2011
	Ministry of Health (Mexico). Mexico Ministry of Health Hospital Discharges. Mexico City, Mexico: Ministry of Health (Mexico).	Zacatecas	2000-2011
Dermatitis	Flohr C, Weinmayr G, Weiland SK, Addo-Yobo E, Annesi-Maesano I, Björkstén B, Bråbäck L,	Albania	2005-2007

	Büchele G, Chico M, Cooper P, Clausen M, El Sharif N, Martinez Gimeno A, Mathur RS, von Mutius E, Morales Suarez-Varela M, Pearce N, Svabe V, Wong GWK, Yu M, Zhong NS, Williams HC, ISAAC Phase Two Study Group. How well do questionnaires perform compared with physical examination in detecting flexural eczema? Findings from the International Study of Asthma and Allergies in Childhood (ISAAC) Phase Two. Br J Dermatol. 2009; 161(4): 846-53.		
	Odhiambo JA, Williams HC, Clayton TO, Robertson CF, Asher MI, ISAAC Phase Three Study Group. Global variations in prevalence of eczema symptoms in children from ISAAC Phase Three. J Allergy Clin Immunol. 2009; 124(6): 1251-1258.	Albania	2000-2002
	Williams H, Stewart A, Von Mutius E, Cookson W, Anderson HR. Is eczema really on the increase worldwide. J Allergy Clin Immunol. 2008; 121(4): 947-954.	Albania	1995-1997, 2000-2001
	Odhiambo JA, Williams HC, Clayton TO, Robertson CF, Asher MI, ISAAC Phase Three Study Group. Global variations in prevalence of eczema symptoms in children from ISAAC Phase Three. J Allergy Clin Immunol. 2009; 124(6): 1251-1258.	Algeria	2002
	Williams H, Stewart A, Von Mutius E, Cookson W, Anderson HR. Is eczema really on the increase worldwide. J Allergy Clin Immunol. 2008; 121(4): 947-954.	Algeria	1996, 2002
	Odhiambo JA, Williams HC, Clayton TO, Robertson CF, Asher MI, ISAAC Phase Three Study Group. Global variations in prevalence of eczema symptoms in children from ISAAC Phase Three. J Allergy Clin Immunol. 2009; 124(6): 1251-1258.	Argentina	2001-2002
	Solé D, Mallol J, Wandalsen GF, Aguirre V, Latin American ISAAC Phase 3 Study Group. Prevalence of symptoms of eczema in Latin America: results of the International Study of Asthma and Allergies in Childhood (ISAAC) Phase 3. J Investig Allergol Clin Immunol. 2010; 20(4): 311-23.	Argentina	2001
	Williams H, Stewart A, Von Mutius E, Cookson W, Anderson HR. Is eczema really on the increase worldwide. J Allergy Clin Immunol. 2008; 121(4): 947-954.	Argentina	1993, 1997, 2002

© 2017 Karimkhani C et al. *JAMA Dermatology.*

	Kljakovic M, Gatenby P, Hawkins C, Attewell RG, Ciszek K, Kratochvil G, Moreira A, Ponsonby A-L. The parent-reported prevalence and management of peanut and nut allergy in school children in the Australian Capital Territory. J Paediatr Child Health. 2009; 45(3): 98-103.	Australia	2005
	Martin PE, Koplin JJ, Eckert JK, Lowe AJ, Ponsonby A-L, Osborne NJ, Gurrin LC, Robinson MN, Hill DJ, Tang MLK, Dharmage SC, Allen KJ, HealthNuts Study Investigators. The prevalence and socio-demographic risk factors of clinical eczema in infancy: a population-based observational study. Clin Exp Allergy. 2013; 43(6): 642-51.	Australia	2008-2011
	Odhiambo JA, Williams HC, Clayton TO, Robertson CF, Asher MI, ISAAC Phase Three Study Group. Global variations in prevalence of eczema symptoms in children from ISAAC Phase Three. J Allergy Clin Immunol. 2009; 124(6): 1251-1258.	Australia	2002
	Ponsonby A-L, Glasgow N, Pezic A, Dwyer T, Ciszek K, Kljakovic M. A temporal decline in asthma but not eczema prevalence from 2000 to 2005 at school entry in the Australian Capital Territory with further consideration of country of birth. Int J Epidemiol. 2008; 37(3): 559-69.	Australia	2000-2005
	Tai A, Volkmer R, Burton A. Association between asthma symptoms and obesity in preschool (4-5 year old) children. J Asthma. 2009; 46(4): 362-5.	Australia	2006
	Tai A, Volkmer R, Burton A. Prevalence of asthma symptoms and atopic disorders in preschool children and the trend over a decade. J Asthma. 2009; 46(4): 343-6.	Australia	2006
	Williams H, Robertson C, Stewart A, Aït-Khaled N, Anabwani G, Anderson R, Asher I, Beasley R, Björkstén B, Burr M, Clayton T, Crane J, Ellwood P, Keil U, Lai C, Mallol J, Martinez F, Mitchell E, Montefort S, Pearce N, Shah J, Sibbald B, Strachan D, von Mutius E, Weiland SK. Worldwide variations in the prevalence of symptoms of atopic eczema in the International Study of Asthma and Allergies in Childhood. J Allergy Clin Immunol. 1999; 103(1 Pt 1): 125-38.	Australia	1997
	Odhiambo JA, Williams HC, Clayton TO, Robertson CF, Asher MI, ISAAC Phase Three Study Group. Global variations in prevalence of eczema symptoms in children from ISAAC Phase	Austria	2002-2003

© 2017 Karimkhani C et al. *JAMA Dermatology.*

	Three. J Allergy Clin Immunol. 2009; 124(6): 1251-1258.		
	Schernhammer ES, Vutuc C, Waldhör T, Haidinger G. Time trends of the prevalence of asthma and allergic disease in Austrian children. Pediatr Allergy Immunol. 2008; 19(2): 125-31.	Austria	1995-1997, 2001-2003
	Weber AS, Haidinger G. The prevalence of atopic dermatitis in children is influenced by their parents' education: results of two cross-sectional studies conducted in Upper Austria. Pediatr Allergy Immunol. 2010; 21(7): 1028-35.	Austria	1995-1997, 2001-2003
	Williams H, Stewart A, Von Mutius E, Cookson W, Anderson HR. Is eczema really on the increase worldwide. J Allergy Clin Immunol. 2008; 121(4): 947-954.	Austria	1995, 2002-2003
	Odhiambo JA, Williams HC, Clayton TO, Robertson CF, Asher MI, ISAAC Phase Three Study Group. Global variations in prevalence of eczema symptoms in children from ISAAC Phase Three. J Allergy Clin Immunol. 2009; 124(6): 1251-1258.	Barbados	2001
	Williams H, Stewart A, Von Mutius E, Cookson W, Anderson HR. Is eczema really on the increase worldwide. J Allergy Clin Immunol. 2008; 121(4): 947-954.	Barbados	1994-1996, 2001, 2003
	Flohr C, Weiland SK, Weinmayr G, Björkstén B, Bråbäck L, Brunekreef B, Büchele G, Clausen M, Cookson WOC, von Mutius E, Strachan DP, Williams HC, ISAAC Phase Two Study Group. The role of atopic sensitization in flexural eczema: findings from the International Study of Asthma and Allergies in Childhood Phase Two. J Allergy Clin Immunol. 2008; 121(1): 141-147.	Beijing	2005-2007
	Odhiambo JA, Williams HC, Clayton TO, Robertson CF, Asher MI, ISAAC Phase Three Study Group. Global variations in prevalence of eczema symptoms in children from ISAAC Phase Three. J Allergy Clin Immunol. 2009; 124(6): 1251-1258.	Beijing	2001
	Williams H, Stewart A, Von Mutius E, Cookson W, Anderson HR. Is eczema really on the increase worldwide. J Allergy Clin Immunol. 2008; 121(4): 947-954.	Beijing	1994, 2001
	Zhao J, Bai J, Shen K, Xiang L, Huang S, Chen A, Huang Y, Wang J, Ye R. Self-reported prevalence of childhood allergic diseases in three cities of	Beijing	2008-2009

© 2017 Karimkhani C et al. *JAMA Dermatology.*

	China: a multicenter study. BMC Public Health. 2010; 551.		
	Shpakou A, Bro?ek G, Stryzhak A, Neviartovich T, Zejda J. Allergic diseases and respiratory symptoms in urban and rural children in Grodno Region (Belarus). Pediatr Allergy Immunol. 2012; 23(4): 339-46.	Belarus	2010
	Chaumont A, Voisin C, Sardella A, Bernard A. Interactions between domestic water hardness, infant swimming and atopy in the development of childhood eczema. Environ Res. 2012; 52-7.	Belgium	2008
	Govaere E, Van Gysel D, Verhamme KMC, Doli E, De Baets F. The association of allergic symptoms with sensitization to inhalant allergens in childhood. Pediatr Allergy Immunol. 2009; 20(5): 448-57.	Belgium	2004-2005
	Govaere E, Van Gysel D, Verhamme KMC, Doli E, Oranje AP, De Baets F. The prevalence, characteristics of and risk factors for eczema in Belgian schoolchildren. Pediatr Dermatol. 2009; 26(2): 129-38.	Belgium	2004-2005
	Odhiambo JA, Williams HC, Clayton TO, Robertson CF, Asher MI, ISAAC Phase Three Study Group. Global variations in prevalence of eczema symptoms in children from ISAAC Phase Three. J Allergy Clin Immunol. 2009; 124(6): 1251-1258.	Belgium	2002
	Van Gysel D, Govaere E, Verhamme K, Doli E, De Baets F. Body mass index in Belgian schoolchildren and its relationship with sensitization and allergic symptoms. Pediatr Allergy Immunol. 2009; 20(3): 246-53.	Belgium	2004-2005
	Williams H, Stewart A, Von Mutius E, Cookson W, Anderson HR. Is eczema really on the increase worldwide. J Allergy Clin Immunol. 2008; 121(4): 947-954.	Belgium	1995, 2002
	Odhiambo JA, Williams HC, Clayton TO, Robertson CF, Asher MI, ISAAC Phase Three Study Group. Global variations in prevalence of eczema symptoms in children from ISAAC Phase Three. J Allergy Clin Immunol. 2009; 124(6): 1251-1258.	Bolivia	2002
	Solé D, Mallol J, Wandalsen GF, Aguirre V, Latin American ISAAC Phase 3 Study Group. Prevalence of symptoms of eczema in Latin America: results of the International Study of Asthma and Allergies in Childhood (ISAAC) Phase 3. J Investig Allergol	Bolivia	2001-2003

© 2017 Karimkhani C et al. *JAMA Dermatology.*

	Clin Immunol. 2010; 20(4): 311-23.		
	Castro LKK de, Cerci Neto A, Ferreira Filho OF. Prevalence of symptoms of asthma, rhinitis and atopic eczema among students between 6 and 7 years of age in the city of Londrina, Brazil. J Bras Pneumol. 2010; 36(3): 286-92.	Brazil	2008
	Freitas MS, Monteiro JCS, Camelo-Nunes IC, Solé D. Prevalence of asthma symptoms and associated factors in schoolchildren from Brazilian Amazon islands. J Asthma. 2012; 49(6): 600-5.	Brazil	2007-2009
	Odhiambo JA, Williams HC, Clayton TO, Robertson CF, Asher MI, ISAAC Phase Three Study Group. Global variations in prevalence of eczema symptoms in children from ISAAC Phase Three. J Allergy Clin Immunol. 2009; 124(6): 1251-1258.	Brazil	2000-2003
	Palvo F, Toledo EC, Menin AM, Jorge PP, Godoy MF, Sole? D. Risk factors of childhood asthma in Sao Jose do Rio Preto, Sao Paulo, Brazil. J Trop Pediatr. 2008; 54(4): 253-7.	Brazil	2003-2004
	Solé D, Mallol J, Wandalsen GF, Aguirre V, Latin American ISAAC Phase 3 Study Group. Prevalence of symptoms of eczema in Latin America: results of the International Study of Asthma and Allergies in Childhood (ISAAC) Phase 3. J Investig Allergol Clin Immunol. 2010; 20(4): 311-23.	Brazil	2001-2003
	Tejada C dos S, Mendoza-Sassi RA, Almeida HL de Jr, Figueiredo PN, Tejada VF dos S. Impact on the quality of life of dermatological patients in southern Brazil. An Bras Dermatol. 2011; 86(6): 1113-21.	Brazil	2008-2009
	Toledo MF, Rozov T, Leone C. Prevalence of asthma and allergies in 13- to 14-year-old adolescents and the frequency of risk factors in carriers of current asthma in Taubaté, São Paulo, Brazil. Allergol Immunopathol (Madr). 2011; 39(5): 284-90.	Brazil	2008-2010
	Williams H, Stewart A, Von Mutius E, Cookson W, Anderson HR. Is eczema really on the increase worldwide. J Allergy Clin Immunol. 2008; 121(4): 947-954,	Brazil	1994-1995, 2001-2003
	Odhiambo JA, Williams HC, Clayton TO, Robertson CF, Asher MI, ISAAC Phase Three Study Group. Global variations in prevalence of eczema symptoms in children from ISAAC Phase Three. J Allergy Clin Immunol. 2009; 124(6): 1251-	Bulgaria	2002

© 2017 Karimkhani C et al. *JAMA Dermatology.*

	1258.		
	Bissek A-CZ-K, Tabah EN, Kouotou E, Sini V, Yepnjio FN, Nditanchou R, Nchufor RN, Defo D, Dema F, Fonsah JY, Njamnshi AK, Muna WFT. The spectrum of skin diseases in a rural setting in Cameroon (sub-Saharan Africa). BMC Dermatol. 2012; 7.	Cameroon	2010
	Odhiambo JA, Williams HC, Clayton TO, Robertson CF, Asher MI, ISAAC Phase Three Study Group. Global variations in prevalence of eczema symptoms in children from ISAAC Phase Three. J Allergy Clin Immunol. 2009; 124(6): 1251-1258.	Cameroon	2003
	Dell SD, Foty RG, Gilbert NL, Jerret M, To T, Walter SD, Stieb DM. Asthma and allergic disease prevalence in a diverse sample of Toronto school children: results from the Toronto Child Health Evaluation Questionnaire (T-CHEQ) Study. Can Respir J. 2010; 17(1): e1-6.	Canada	2006
	Odhiambo JA, Williams HC, Clayton TO, Robertson CF, Asher MI, ISAAC Phase Three Study Group. Global variations in prevalence of eczema symptoms in children from ISAAC Phase Three. J Allergy Clin Immunol. 2009; 124(6): 1251-1258.	Canada	2003
	Wang H-Y, Pizzichini MMM, Becker AB, Duncan JM, Ferguson AC, Greene JM, Rennie DC, Senthilselvan A, Taylor BW, Sears MR. Disparate geographic prevalences of asthma, allergic rhinoconjunctivitis and atopic eczema among adolescents in five Canadian cities. Pediatr Allergy Immunol. 2010; 21(5): 867-77.	Canada	2003
	Odhiambo JA, Williams HC, Clayton TO, Robertson CF, Asher MI, ISAAC Phase Three Study Group. Global variations in prevalence of eczema symptoms in children from ISAAC Phase Three. J Allergy Clin Immunol. 2009; 124(6): 1251-1258.	Chihuahua	2002-2003
	Solé D, Mallol J, Wandalsen GF, Aguirre V, Latin American ISAAC Phase 3 Study Group. Prevalence of symptoms of eczema in Latin America: results of the International Study of Asthma and Allergies in Childhood (ISAAC) Phase 3. J Investig Allergol Clin Immunol. 2010; 20(4): 311-23.	Chihuahua	2001-2003
	Odhiambo JA, Williams HC, Clayton TO, Robertson CF, Asher MI, ISAAC Phase Three	Chile	2001-2002

© 2017 Karimkhani C et al. *JAMA Dermatology.*

	Study Group. Global variations in prevalence of eczema symptoms in children from ISAAC Phase Three. J Allergy Clin Immunol. 2009; 124(6): 1251-1258.		
	Solé D, Mallol J, Wandalsen GF, Aguirre V, Latin American ISAAC Phase 3 Study Group. Prevalence of symptoms of eczema in Latin America: results of the International Study of Asthma and Allergies in Childhood (ISAAC) Phase 3. J Investig Allergol Clin Immunol. 2010; 20(4): 311-23.	Chile	2001-2003
	Williams H, Stewart A, Von Mutius E, Cookson W, Anderson HR. Is eczema really on the increase worldwide. J Allergy Clin Immunol. 2008; 121(4): 947-954.	Chile	1994-1995, 2001
	Lee J, Koh D, Andijani M, Saw SM, Munoz C, Chia SE, Wong ML, Hong CY, Ong CN. Effluents from a pulp and paper mill: a skin and health survey of children living in upstream and downstream villages. Occup Environ Med. 2002; 59(6): 373-9.	China	2001, 2005
	Li F, Zhou Y, Li S, Jiang F, Jin X, Yan C, Tian Y, Zhang Y, Tong S, Shen X. Prevalence and risk factors of childhood allergic diseases in eight metropolitan cities in China: a multicenter study. BMC Public Health. 2011; 437.	China	2005
	Williams H, Stewart A, Von Mutius E, Cookson W, Anderson HR. Is eczema really on the increase worldwide. J Allergy Clin Immunol. 2008; 121(4): 947-954.	China	1994-1995, 2001
	Zhao J, Bai J, Shen K, Xiang L, Huang S, Chen A, Huang Y, Wang J, Ye R. Self-reported prevalence of childhood allergic diseases in three cities of China: a multicenter study. BMC Public Health. 2010; 551.	Chongqing	2008-2009
	Dennis RJ, Caraballo L, García E, Rojas MX, Rondon MA, Pérez A, Aristizabal G, Peñaranda A, Barragan AM, Ahumada V, Jimenez S. Prevalence of asthma and other allergic conditions in Colombia 2009-2010: a cross-sectional study. BMC Pulm Med. 2012; 17.	Colombia	2009-2010
	Odhiambo JA, Williams HC, Clayton TO, Robertson CF, Asher MI, ISAAC Phase Three Study Group. Global variations in prevalence of eczema symptoms in children from ISAAC Phase Three. J Allergy Clin Immunol. 2009; 124(6): 1251-1258.	Colombia	2002
	Solé D, Mallol J, Wandalsen GF, Aguirre V, Latin	Colombia	2001-

© 2017 Karimkhani C et al. *JAMA Dermatology.*

	American ISAAC Phase 3 Study Group. Prevalence of symptoms of eczema in Latin America: results of the International Study of Asthma and Allergies in Childhood (ISAAC) Phase 3. J Investig Allergol Clin Immunol. 2010; 20(4): 311-23.		2003
	Odhiambo JA, Williams HC, Clayton TO, Robertson CF, Asher MI, ISAAC Phase Three Study Group. Global variations in prevalence of eczema symptoms in children from ISAAC Phase Three. J Allergy Clin Immunol. 2009; 124(6): 1251-1258.	Congo	2002
	Odhiambo JA, Williams HC, Clayton TO, Robertson CF, Asher MI, ISAAC Phase Three Study Group. Global variations in prevalence of eczema symptoms in children from ISAAC Phase Three. J Allergy Clin Immunol. 2009; 124(6): 1251-1258.	Costa Rica	2002
	Solé D, Mallol J, Wandalsen GF, Aguirre V, Latin American ISAAC Phase 3 Study Group. Prevalence of symptoms of eczema in Latin America: results of the International Study of Asthma and Allergies in Childhood (ISAAC) Phase 3. J Investig Allergol Clin Immunol. 2010; 20(4): 311-23.	Costa Rica	2001-2003
	Williams H, Stewart A, Von Mutius E, Cookson W, Anderson HR. Is eczema really on the increase worldwide. J Allergy Clin Immunol. 2008; 121(4): 947-954.	Costa Rica	1994, 2002
	Odhiambo JA, Williams HC, Clayton TO, Robertson CF, Asher MI, ISAAC Phase Three Study Group. Global variations in prevalence of eczema symptoms in children from ISAAC Phase Three. J Allergy Clin Immunol. 2009; 124(6): 1251-1258.	Cote D'Ivoire	2001
	Odhiambo JA, Williams HC, Clayton TO, Robertson CF, Asher MI, ISAAC Phase Three Study Group. Global variations in prevalence of eczema symptoms in children from ISAAC Phase Three. J Allergy Clin Immunol. 2009; 124(6): 1251-1258.	Croatia	2002
	Odhiambo JA, Williams HC, Clayton TO, Robertson CF, Asher MI, ISAAC Phase Three Study Group. Global variations in prevalence of eczema symptoms in children from ISAAC Phase Three. J Allergy Clin Immunol. 2009; 124(6): 1251-1258.	Cuba	2002
	Solé D, Mallol J, Wandalsen GF, Aguirre V, Latin	Cuba	2001-

© 2017 Karimkhani C et al. *JAMA Dermatology.*

	American ISAAC Phase 3 Study Group. Prevalence of symptoms of eczema in Latin America: results of the International Study of Asthma and Allergies in Childhood (ISAAC) Phase 3. J Investig Allergol Clin Immunol. 2010; 20(4): 311-23.		2003
	Kolokotroni O, Middleton N, Nicolaou N, Pipis S, Priftis KN, Milton DK, Yiallouros PK. Temporal changes in the prevalence of childhood asthma and allergies in urban and rural areas of Cyprus: results from two cross sectional studies. BMC Public Health. 2011; 858.	Cyprus	1999-2000, 2007-2008
	Odhiambo JA, Williams HC, Clayton TO, Robertson CF, Asher MI, ISAAC Phase Three Study Group. Global variations in prevalence of eczema symptoms in children from ISAAC Phase Three. J Allergy Clin Immunol. 2009; 124(6): 1251-1258.	Democratic Republic of the Congo	2003
	Stensen L, Thomsen SF, Backer V. Change in prevalence of atopic dermatitis between 1986 and 2001 among children. Allergy Asthma Proc. 2008; 29(4): 392-6.	Denmark	1986, 2001
	Odhiambo JA, Williams HC, Clayton TO, Robertson CF, Asher MI, ISAAC Phase Three Study Group. Global variations in prevalence of eczema symptoms in children from ISAAC Phase Three. J Allergy Clin Immunol. 2009; 124(6): 1251-1258.	Distrito Federal	2002
	Solé D, Mallol J, Wandalsen GF, Aguirre V, Latin American ISAAC Phase 3 Study Group. Prevalence of symptoms of eczema in Latin America: results of the International Study of Asthma and Allergies in Childhood (ISAAC) Phase 3. J Investig Allergol Clin Immunol. 2010; 20(4): 311-23.	Distrito Federal	2001-2003
	Solé D, Mallol J, Wandalsen GF, Aguirre V, Latin American ISAAC Phase 3 Study Group. Prevalence of symptoms of eczema in Latin America: results of the International Study of Asthma and Allergies in Childhood (ISAAC) Phase 3. J Investig Allergol Clin Immunol. 2010; 20(4): 311-23.	Durango	2001-2003
	Flohr C, Weiland SK, Weinmayr G, Björkstén B, Bråbäck L, Brunekreef B, Büchele G, Clausen M, Cookson WOC, von Mutius E, Strachan DP, Williams HC, ISAAC Phase Two Study Group. The role of atopic sensitization in flexural eczema: findings from the International Study of Asthma and Allergies in Childhood Phase Two. J Allergy	Ecuador	2005-2007

	Clin Immunol. 2008; 121(1): 141-147.		
	Odhiambo JA, Williams HC, Clayton TO, Robertson CF, Asher MI, ISAAC Phase Three Study Group. Global variations in prevalence of eczema symptoms in children from ISAAC Phase Three. J Allergy Clin Immunol. 2009; 124(6): 1251-1258.	Ecuador	2002-2003
	Solé D, Mallol J, Wandalsen GF, Aguirre V, Latin American ISAAC Phase 3 Study Group. Prevalence of symptoms of eczema in Latin America: results of the International Study of Asthma and Allergies in Childhood (ISAAC) Phase 3. J Investig Allergol Clin Immunol. 2010; 20(4): 311-23.	Ecuador	2001-2003
	Odhiambo JA, Williams HC, Clayton TO, Robertson CF, Asher MI, ISAAC Phase Three Study Group. Global variations in prevalence of eczema symptoms in children from ISAAC Phase Three. J Allergy Clin Immunol. 2009; 124(6): 1251-1258.	Egypt	2002
	Yamamah GA, Emam HM, Abdelhamid MF, Elsaie ML, Shehata H, Farid T, Kamel MI, Taalat AA. Epidemiologic study of dermatologic disorders among children in South Sinai, Egypt. Int J Dermatol. 2012; 51(10): 1180-5.	Egypt	2008-2009
	Odhiambo JA, Williams HC, Clayton TO, Robertson CF, Asher MI, ISAAC Phase Three Study Group. Global variations in prevalence of eczema symptoms in children from ISAAC Phase Three. J Allergy Clin Immunol. 2009; 124(6): 1251-1258.	El Salvador	2003
	Solé D, Mallol J, Wandalsen GF, Aguirre V, Latin American ISAAC Phase 3 Study Group. Prevalence of symptoms of eczema in Latin America: results of the International Study of Asthma and Allergies in Childhood (ISAAC) Phase 3. J Investig Allergol Clin Immunol. 2010; 20(4): 311-23.	El Salvador	2001-2003
	Flohr C, Weiland SK, Weinmayr G, Björkstén B, Bråbäck L, Brunekreef B, Büchele G, Clausen M, Cookson WOC, von Mutius E, Strachan DP, Williams HC, ISAAC Phase Two Study Group. The role of atopic sensitization in flexural eczema: findings from the International Study of Asthma and Allergies in Childhood Phase Two. J Allergy Clin Immunol. 2008; 121(1): 141-147.	Estonia	2005-2007
	Odhiambo JA, Williams HC, Clayton TO, Robertson CF, Asher MI, ISAAC Phase Three	Estonia	2001

© 2017 Karimkhani C et al. *JAMA Dermatology.*

	Study Group. Global variations in prevalence of eczema symptoms in children from ISAAC Phase Three. J Allergy Clin Immunol. 2009; 124(6): 1251-1258.		
	Vasar M, Julge K, Kivivare M, Otter K. Regional differences in diagnosing asthma and other allergic diseases in Estonian schoolchildren. Medicina (Kaunas). 2011; 47(12): 661-6.	Estonia	2003
	Williams H, Stewart A, Von Mutius E, Cookson W, Anderson HR. Is eczema really on the increase worldwide. J Allergy Clin Immunol. 2008; 121(4): 947-954.	Estonia	1994, 2001
	Ali J, Yifru S, Woldeamanuel Y. Prevalence of tinea capitis and the causative agent among school children in Gondar, North West Ethiopia. Ethiop Med J. 2011; 47(4): 261-9.	Ethiopia	2007-2008
	Belyhun Y, Amberbir A, Medhin G, Erko B, Hanlon C, Venn A, Britton J, Davey G. Prevalence and risk factors of wheeze and eczema in 1-year-old children: the Butajira birth cohort, Ethiopia. Clin Exp Allergy. 2010; 40(4): 619-26.	Ethiopia	2006
	Murgia V, Bilcha KD, Shibeshi D. Community dermatology in Debre Markos: an attempt to define children's dermatological needs in a rural area of Ethiopia. Int J Dermatol. 2010; 49(6): 666-71.	Ethiopia	2009
	Odhiambo JA, Williams HC, Clayton TO, Robertson CF, Asher MI, ISAAC Phase Three Study Group. Global variations in prevalence of eczema symptoms in children from ISAAC Phase Three. J Allergy Clin Immunol. 2009; 124(6): 1251-1258.	Ethiopia	2003
	Williams H, Stewart A, Von Mutius E, Cookson W, Anderson HR. Is eczema really on the increase worldwide. J Allergy Clin Immunol. 2008; 121(4): 947-954.	Ethiopia	1995, 2003
	Woldeamanuel Y, Leekassa R, Chryssanthou E, Menghistu Y, Petrini B. Prevalence of tinea capitis in Ethiopian schoolchildren. Mycoses. 2005; 48(2): 137-41.	Ethiopia	2003-2005
	Foliaki S, Annesi-Measano I, Daniel R, Fakakovikaetau T, Magatongia M, Tuuau-Potoi N, Waqatakirewa L, Cheng S, Pearce N. Prevalence of symptoms of childhood asthma, allergic rhinoconjunctivitis and eczema in the Pacific: the International Study of Asthma and Allergies in Childhood (ISAAC). Allergy. 2007; 62(3): 259-64.	Fiji	1998-2003

© 2017 Karimkhani C et al. *JAMA Dermatology.*

	Odhiambo JA, Williams HC, Clayton TO, Robertson CF, Asher MI, ISAAC Phase Three Study Group. Global variations in prevalence of eczema symptoms in children from ISAAC Phase Three. J Allergy Clin Immunol. 2009; 124(6): 1251-1258.	Fiji	2002
	Hatakka K, Piirainen L, Pohjavuori S, Poussa T, Savilahti E, Korpela R. Allergy in day care children: prevalence and environmental risk factors. Acta Paediatr. 2009; 98(5): 817-22.	Finland	1998
	Hugg T, Ruotsalainen R, Jaakkola MS, Pushkarev V, Jaakkola JJK. Comparison of allergic diseases, symptoms and respiratory infections between Finnish and Russian school children. Eur J Epidemiol. 2008; 23(2): 123-33.	Finland	2003, 2005
	Odhiambo JA, Williams HC, Clayton TO, Robertson CF, Asher MI, ISAAC Phase Three Study Group. Global variations in prevalence of eczema symptoms in children from ISAAC Phase Three. J Allergy Clin Immunol. 2009; 124(6): 1251-1258.	Finland	2001
	Williams H, Stewart A, Von Mutius E, Cookson W, Anderson HR. Is eczema really on the increase worldwide. J Allergy Clin Immunol. 2008; 121(4): 947-954.	Finland	1994, 2001
	Annesi-Maesano I, Mourad C, Daures J-P, Kalaboka S, Godard P. Time trends in prevalence and severity of childhood asthma and allergies from 1995 to 2002 in France. Allergy. 2009; 64(5): 798-800.	France	1994, 2002
	Flohr C, Weinmayr G, Weiland SK, Addo-Yobo E, Annesi-Maesano I, Björkstén B, Bråbäck L, Büchele G, Chico M, Cooper P, Clausen M, El Sharif N, Martinez Gimeno A, Mathur RS, von Mutius E, Morales Suarez-Varela M, Pearce N, Svabe V, Wong GWK, Yu M, Zhong NS, Williams HC, ISAAC Phase Two Study Group. How well do questionnaires perform compared with physical examination in detecting flexural eczema? Findings from the International Study of Asthma and Allergies in Childhood (ISAAC) Phase Two. Br J Dermatol. 2009; 161(4): 846-53.	France	2005-2007
	Hogewoning A, Amoah A, Bavinck JNB, Boakye D, Yazdanbakhsh M, Adegnika A, De Smedt S, Fonteyne Y, Willemze R, Lavrijsen A. Skin diseases among schoolchildren in Ghana, Gabon,	Gabon	2005-2007

© 2017 Karimkhani C et al. *JAMA Dermatology.*

	and Rwanda. Int J Dermatol. 2013; 52(5): 589-600.		
	Hogewoning AA, Bouwes Bavinck JN, Amoah AS, Boakye DA, Yazdanbakhsh M, Kremsner PG, Adegnika AA, De Smedt SKAD, Willemze R, Lavrijsen APM. Point and period prevalences of eczema in rural and urban schoolchildren in Ghana, Gabon and Rwanda. J Eur Acad Dermatol Venereol. 2012; 26(4): 488-94.	Gabon	2005
	Odhiambo JA, Williams HC, Clayton TO, Robertson CF, Asher MI, ISAAC Phase Three Study Group. Global variations in prevalence of eczema symptoms in children from ISAAC Phase Three. J Allergy Clin Immunol. 2009; 124(6): 1251-1258.	Gabon	2003
	Flohr C, Weiland SK, Weinmayr G, Björkstén B, Bråbäck L, Brunekreef B, Büchele G, Clausen M, Cookson WOC, von Mutius E, Strachan DP, Williams HC, ISAAC Phase Two Study Group. The role of atopic sensitization in flexural eczema: findings from the International Study of Asthma and Allergies in Childhood Phase Two. J Allergy Clin Immunol. 2008; 121(1): 141-147.	Georgia	2005-2007
	Odhiambo JA, Williams HC, Clayton TO, Robertson CF, Asher MI, ISAAC Phase Three Study Group. Global variations in prevalence of eczema symptoms in children from ISAAC Phase Three. J Allergy Clin Immunol. 2009; 124(6): 1251-1258.	Georgia	2003
	Williams H, Stewart A, Von Mutius E, Cookson W, Anderson HR. Is eczema really on the increase worldwide. J Allergy Clin Immunol. 2008; 121(4): 947-954.	Georgia	1996, 2003
	Augustin M, Herberger K, Hintzen S, Heigel H, Franzke N, Schäfer I. Prevalence of skin lesions and need for treatment in a cohort of 90,880 workers. Br J Dermatol. 2011; 165(4): 865-73.	Germany	2002-2009
	Cramer C, Link E, Bauer C-P, Hoffmann U, von Berg A, Lehmann I, Herbarth O, Borte M, Schaaf B, Sausenthaler S, Wichmann H-E, Heinrich J, Krämer U, LISAplus study group. Association between attendance of day care centres and increased prevalence of eczema in the German birth cohort study LISAplus. Allergy. 2011; 66(1): 68-75.	Germany	1997-1999
	Flohr C, Weiland SK, Weinmayr G, Björkstén B, Bråbäck L, Brunekreef B, Büchele G, Clausen M, Cookson WOC, von Mutius E, Strachan DP,	Germany	2005-2007

	Williams HC, ISAAC Phase Two Study Group. The role of atopic sensitization in flexural eczema: findings from the International Study of Asthma and Allergies in Childhood Phase Two. J Allergy Clin Immunol. 2008; 121(1): 141-147.		
	Frese T, Herrmann K, Sandholzer H. Pruritus as Reason for Encounter in General Practice. J Clin Med Res. 2011; 3(5): 223-9.	Germany	1999-2000
	Langen U, Schmitz R, Steppuhn H. [Prevalence of allergic diseases in Germany: results of the German Health Interview and Examination Survey for Adults (DEGS1)]. Bundesgesundheitsblatt. 2013; 56(5-6): 698-706.	Germany	2008-2011
	Odhiambo JA, Williams HC, Clayton TO, Robertson CF, Asher MI, ISAAC Phase Three Study Group. Global variations in prevalence of eczema symptoms in children from ISAAC Phase Three. J Allergy Clin Immunol. 2009; 124(6): 1251-1258.	Germany	1999
	Romanos M, Gerlach M, Warnke A, Schmitt J. Association of attention-deficit/hyperactivity disorder and atopic eczema modified by sleep disturbance in a large population-based sample. J Epidemiol Community Health. 2010; 64(3): 269-73.	Germany	2003-2006
	Schaefer I, Rustenbach SJ, Zimmer L, Augustin M. Prevalence of skin diseases in a cohort of 48,665 employees in Germany. Dermatology (Basel). 2008; 217(2): 169-72.	Germany	2001-2005
	Schäfer T, Stieger B, Polzius R, Krauspe A. Associations between cat keeping, allergen exposure, allergic sensitization and atopic diseases: results from the Children of Lübeck Allergy and Environment Study (KLAUS). Pediatr Allergy Immunol. 2009; 20(4): 353-7.	Germany	2003
	Ständer S, Schäfer I, Phan NQ, Blome C, Herberger K, Heigel H, Augustin M. Prevalence of chronic pruritus in Germany: results of a cross-sectional study in a sample working population of 11,730. Dermatology (Basel). 2010; 221(3): 229-35.	Germany	2008
	Williams H, Stewart A, Von Mutius E, Cookson W, Anderson HR. Is eczema really on the increase worldwide. J Allergy Clin Immunol. 2008; 121(4): 947-954.	Germany	1994, 1999
	Flohr C, Weiland SK, Weinmayr G, Björkstén B, Bråbäck L, Brunekreef B, Büchele G, Clausen M,	Ghana	2005-2007

© 2017 Karimkhani C et al. *JAMA Dermatology.*

	Cookson WOC, von Mutius E, Strachan DP, Williams HC, ISAAC Phase Two Study Group. The role of atopic sensitization in flexural eczema: findings from the International Study of Asthma and Allergies in Childhood Phase Two. J Allergy Clin Immunol. 2008; 121(1): 141-147.		
	Flohr C, Weinmayr G, Weiland SK, Addo-Yobo E, Annesi-Maesano I, Björkstén B, Bråbäck L, Büchele G, Chico M, Cooper P, Clausen M, El Sharif N, Martinez Gimeno A, Mathur RS, von Mutius E, Morales Suarez-Varela M, Pearce N, Svabe V, Wong GWK, Yu M, Zhong NS, Williams HC, ISAAC Phase Two Study Group. How well do questionnaires perform compared with physical examination in detecting flexural eczema? Findings from the International Study of Asthma and Allergies in Childhood (ISAAC) Phase Two. Br J Dermatol. 2009; 161(4): 846-53.	Ghana	2005-2007
	Hogewoning A, Amoah A, Bavinck JNB, Boakye D, Yazdanbakhsh M, Adegnika A, De Smedt S, Fonteyne Y, Willemze R, Lavrijsen A. Skin diseases among schoolchildren in Ghana, Gabon, and Rwanda. Int J Dermatol. 2013; 52(5): 589-600.	Ghana	2004, 2007
	Hogewoning AA, Bouwes Bavinck JN, Amoah AS, Boakye DA, Yazdanbakhsh M, Kremsner PG, Adegnika AA, De Smedt SKAD, Willemze R, Lavrijsen APM. Point and period prevalences of eczema in rural and urban schoolchildren in Ghana, Gabon and Rwanda. J Eur Acad Dermatol Venereol. 2012; 26(4): 488-94.	Ghana	2004, 2007
	Odhiambo JA, Williams HC, Clayton TO, Robertson CF, Asher MI, ISAAC Phase Three Study Group. Global variations in prevalence of eczema symptoms in children from ISAAC Phase Three. J Allergy Clin Immunol. 2009; 124(6): 1251-1258.	Greater London	2002
	Williams H, Stewart A, Von Mutius E, Cookson W, Anderson HR. Is eczema really on the increase worldwide. J Allergy Clin Immunol. 2008; 121(4): 947-954.	Greater London	1995, 2002
	Anthracopoulos MB, Antonogeorgos G, Liolios E, Triga M, Panagiotopoulou E, Priftis KN. Increase in chronic or recurrent rhinitis, rhinoconjunctivitis and eczema among schoolchildren in Greece: three surveys during 1991-2003. Pediatr Allergy Immunol. 2009; 20(2): 180-6.	Greece	1991, 1998, 2003

© 2017 Karimkhani C et al. *JAMA Dermatology.*

	Flohr C, Weiland SK, Weinmayr G, Björkstén B, Bråbäck L, Brunekreef B, Büchele G, Clausen M, Cookson WOC, von Mutius E, Strachan DP, Williams HC, ISAAC Phase Two Study Group. The role of atopic sensitization in flexural eczema: findings from the International Study of Asthma and Allergies in Childhood Phase Two. J Allergy Clin Immunol. 2008; 121(1): 141-147.	Greece	2005, 2007
	Flohr C, Weinmayr G, Weiland SK, Addo-Yobo E, Annesi-Maesano I, Björkstén B, Bråbäck L, Büchele G, Chico M, Cooper P, Clausen M, El Sharif N, Martinez Gimeno A, Mathur RS, von Mutius E, Morales Suarez-Varela M, Pearce N, Svabe V, Wong GWK, Yu M, Zhong NS, Williams HC, ISAAC Phase Two Study Group. How well do questionnaires perform compared with physical examination in detecting flexural eczema? Findings from the International Study of Asthma and Allergies in Childhood (ISAAC) Phase Two. Br J Dermatol. 2009; 161(4): 846-53.	Greece	2005-2007
	Odhiambo JA, Williams HC, Clayton TO, Robertson CF, Asher MI, ISAAC Phase Three Study Group. Global variations in prevalence of eczema symptoms in children from ISAAC Phase Three. J Allergy Clin Immunol. 2009; 124(6): 1251-1258.	Greece	2000
	Flohr C, Weiland SK, Weinmayr G, Björkstén B, Bråbäck L, Brunekreef B, Büchele G, Clausen M, Cookson WOC, von Mutius E, Strachan DP, Williams HC, ISAAC Phase Two Study Group. The role of atopic sensitization in flexural eczema: findings from the International Study of Asthma and Allergies in Childhood Phase Two. J Allergy Clin Immunol. 2008; 121(1): 141-147.	Guangdong	2005-2007
	Flohr C, Weinmayr G, Weiland SK, Addo-Yobo E, Annesi-Maesano I, Björkstén B, Bråbäck L, Büchele G, Chico M, Cooper P, Clausen M, El Sharif N, Martinez Gimeno A, Mathur RS, von Mutius E, Morales Suarez-Varela M, Pearce N, Svabe V, Wong GWK, Yu M, Zhong NS, Williams HC, ISAAC Phase Two Study Group. How well do questionnaires perform compared with physical examination in detecting flexural eczema? Findings from the International Study of Asthma and Allergies in Childhood (ISAAC) Phase Two. Br J Dermatol. 2009; 161(4): 846-53.	Guangdong	2005-2007

© 2017 Karimkhani C et al. *JAMA Dermatology.*

	Li F, Zhou Y, Li S, Jiang F, Jin X, Yan C, Tian Y, Zhang Y, Tong S, Shen X. Prevalence and risk factors of childhood allergic diseases in eight metropolitan cities in China: a multicenter study. BMC Public Health. 2011; 437.	Guangdong	2005
	Odhiambo JA, Williams HC, Clayton TO, Robertson CF, Asher MI, ISAAC Phase Three Study Group. Global variations in prevalence of eczema symptoms in children from ISAAC Phase Three. J Allergy Clin Immunol. 2009; 124(6): 1251-1258.	Guangdong	2001
	Williams H, Stewart A, Von Mutius E, Cookson W, Anderson HR. Is eczema really on the increase worldwide. J Allergy Clin Immunol. 2008; 121(4): 947-954.	Guangdong	1994, 2001
	Zhao J, Bai J, Shen K, Xiang L, Huang S, Chen A, Huang Y, Wang J, Ye R. Self-reported prevalence of childhood allergic diseases in three cities of China: a multicenter study. BMC Public Health. 2010; 551.	Guangdong	2008-2009
	Hay RJ, Castanon RE, Hernandez HA, Lopez GC, Fuentes LF, Solis SP, Andersson N. Wastage of family income on skin disease in Mexico. BMJ. 1994; 309(6958): 848.	Guerrero	1993
	Odhiambo JA, Williams HC, Clayton TO, Robertson CF, Asher MI, ISAAC Phase Three Study Group. Global variations in prevalence of eczema symptoms in children from ISAAC Phase Three. J Allergy Clin Immunol. 2009; 124(6): 1251-1258.	Guinea	1997
	Li F, Zhou Y, Li S, Jiang F, Jin X, Yan C, Tian Y, Zhang Y, Tong S, Shen X. Prevalence and risk factors of childhood allergic diseases in eight metropolitan cities in China: a multicenter study. BMC Public Health. 2011; 437.	Heilongjiang	2005
	Odhiambo JA, Williams HC, Clayton TO, Robertson CF, Asher MI, ISAAC Phase Three Study Group. Global variations in prevalence of eczema symptoms in children from ISAAC Phase Three. J Allergy Clin Immunol. 2009; 124(6): 1251-1258.	Honduras	2002
	Solé D, Mallol J, Wandalsen GF, Aguirre V, Latin American ISAAC Phase 3 Study Group. Prevalence of symptoms of eczema in Latin America: results of the International Study of Asthma and Allergies in Childhood (ISAAC) Phase 3. J Investig Allergol	Honduras	2001-2003

© 2017 Karimkhani C et al. *JAMA Dermatology.*

	Clin Immunol. 2010; 20(4): 311-23.		
	Flohr C, Weiland SK, Weinmayr G, Björkstén B, Bråbäck L, Brunekreef B, Büchele G, Clausen M, Cookson WOC, von Mutius E, Strachan DP, Williams HC, ISAAC Phase Two Study Group. The role of atopic sensitization in flexural eczema: findings from the International Study of Asthma and Allergies in Childhood Phase Two. J Allergy Clin Immunol. 2008; 121(1): 141-147.	Hong Kong Special Administrative Region of China	2005-2007
	Flohr C, Weinmayr G, Weiland SK, Addo-Yobo E, Annesi-Maesano I, Björkstén B, Bråbäck L, Büchele G, Chico M, Cooper P, Clausen M, El Sharif N, Martinez Gimeno A, Mathur RS, von Mutius E, Morales Suarez-Varela M, Pearce N, Svabe V, Wong GWK, Yu M, Zhong NS, Williams HC, ISAAC Phase Two Study Group. How well do questionnaires perform compared with physical examination in detecting flexural eczema? Findings from the International Study of Asthma and Allergies in Childhood (ISAAC) Phase Two. Br J Dermatol. 2009; 161(4): 846-53.	Hong Kong Special Administrative Region of China	2005-2007
	Fung WK, Lo KK. Prevalence of skin disease among school children and adolescents in a Student Health Service Center in Hong Kong. Pediatr Dermatol. 2000; 17(6): 440-6.	Hong Kong Special Administrative Region of China	1996-1997
	Odhiambo JA, Williams HC, Clayton TO, Robertson CF, Asher MI, ISAAC Phase Three Study Group. Global variations in prevalence of eczema symptoms in children from ISAAC Phase Three. J Allergy Clin Immunol. 2009; 124(6): 1251-1258.	Hong Kong Special Administrative Region of China	2001, 2002
	Williams H, Stewart A, Von Mutius E, Cookson W, Anderson HR. Is eczema really on the increase worldwide. J Allergy Clin Immunol. 2008; 121(4): 947-954.	Hong Kong Special Administrative Region of China	1995, 2001-2002
	Li F, Zhou Y, Li S, Jiang F, Jin X, Yan C, Tian Y, Zhang Y, Tong S, Shen X. Prevalence and risk factors of childhood allergic diseases in eight metropolitan cities in China: a multicenter study. BMC Public Health. 2011; 437.	Hubei	2005
	Kuhnyar A, Egyud K, Szabo I, Hunyadi J, Kosa L. Prevalence of atopic dermatitis among children under 19 in an East-Hungarian agricultural county. Clin Dev Immunol. 2006; 13(4-Feb): 395-9.	Hungary	2004

© 2017 Karimkhani C et al. *JAMA Dermatology.*

	Odhiambo JA, Williams HC, Clayton TO, Robertson CF, Asher MI, ISAAC Phase Three Study Group. Global variations in prevalence of eczema symptoms in children from ISAAC Phase Three. J Allergy Clin Immunol. 2009; 124(6): 1251-1258.	Hungary	2003
	Flohr C, Weiland SK, Weinmayr G, Björkstén B, Bråbäck L, Brunekreef B, Büchele G, Clausen M, Cookson WOC, von Mutius E, Strachan DP, Williams HC, ISAAC Phase Two Study Group. The role of atopic sensitization in flexural eczema: findings from the International Study of Asthma and Allergies in Childhood Phase Two. J Allergy Clin Immunol. 2008; 121(1): 141-147.	Iceland	2005-2007
	Flohr C, Weinmayr G, Weiland SK, Addo-Yobo E, Annesi-Maesano I, Björkstén B, Bråbäck L, Büchele G, Chico M, Cooper P, Clausen M, El Sharif N, Martinez Gimeno A, Mathur RS, von Mutius E, Morales Suarez-Varela M, Pearce N, Svabe V, Wong GWK, Yu M, Zhong NS, Williams HC, ISAAC Phase Two Study Group. How well do questionnaires perform compared with physical examination in detecting flexural eczema? Findings from the International Study of Asthma and Allergies in Childhood (ISAAC) Phase Two. Br J Dermatol. 2009; 161(4): 846-53.	Iceland	2005-2007
	Dogra S, Kumar B. Epidemiology of skin diseases in school children: a study from northern India. Pediatr Dermatol. 2003; 20(6): 470-3.	India	2001
	Flohr C, Weiland SK, Weinmayr G, Björkstén B, Bråbäck L, Brunekreef B, Büchele G, Clausen M, Cookson WOC, von Mutius E, Strachan DP, Williams HC, ISAAC Phase Two Study Group. The role of atopic sensitization in flexural eczema: findings from the International Study of Asthma and Allergies in Childhood Phase Two. J Allergy Clin Immunol. 2008; 121(1): 141-147.	India	2005-2007
	Flohr C, Weinmayr G, Weiland SK, Addo-Yobo E, Annesi-Maesano I, Björkstén B, Bråbäck L, Büchele G, Chico M, Cooper P, Clausen M, El Sharif N, Martinez Gimeno A, Mathur RS, von Mutius E, Morales Suarez-Varela M, Pearce N, Svabe V, Wong GWK, Yu M, Zhong NS, Williams HC, ISAAC Phase Two Study Group. How well do questionnaires perform compared with physical examination in detecting flexural eczema? Findings	India	2005-2007

© 2017 Karimkhani C et al. *JAMA Dermatology.*

	from the International Study of Asthma and Allergies in Childhood (ISAAC) Phase Two. Br J Dermatol. 2009; 161(4): 846-53.		
	Grills N, Grills C, Spelman T, Stoove M, Hellard M, El-Hayek C, Singh R. Prevalence survey of dermatological conditions in mountainous north India. Int J Dermatol. 2012; 51(5): 579-87.	India	2010
	Grover S, Ranyal RK, Bedi MK. A cross section of skin diseases in rural Allahabad. Indian J Dermatol. 2008; 53(4): 179-81.	India	2005
	Kuruvila M, Dubey S, Gahalaut P. Pattern of skin diseases among migrant construction workers in Mangalore. Indian J Dermatol Venereol Leprol. 2006; 72(2): 129-32.	India	2005
	Odhiambo JA, Williams HC, Clayton TO, Robertson CF, Asher MI, ISAAC Phase Three Study Group. Global variations in prevalence of eczema symptoms in children from ISAAC Phase Three. J Allergy Clin Immunol. 2009; 124(6): 1251-1258.	India	2001-2003
	Patel JK, Vyas AP, Berman B, Vierra M. Incidence of childhood dermatosis in India. Skinmed. 2010; 8(3): 136-42.	India	2000-2002
	Williams H, Stewart A, Von Mutius E, Cookson W, Anderson HR. Is eczema really on the increase worldwide. J Allergy Clin Immunol. 2008; 121(4): 947-954.		1994-1995, 2001-2003
	Lee J, Koh D, Andijani M, Saw SM, Munoz C, Chia SE, Wong ML, Hong CY, Ong CN. Effluents from a pulp and paper mill: a skin and health survey of children living in upstream and downstream villages. Occup Environ Med. 2002; 59(6): 373-9.	Indonesia	1999
	Odhiambo JA, Williams HC, Clayton TO, Robertson CF, Asher MI, ISAAC Phase Three Study Group. Global variations in prevalence of eczema symptoms in children from ISAAC Phase Three. J Allergy Clin Immunol. 2009; 124(6): 1251-1258.	Indonesia	2001-2002
	Williams H, Stewart A, Von Mutius E, Cookson W, Anderson HR. Is eczema really on the increase worldwide. J Allergy Clin Immunol. 2008; 121(4): 947-954.	Indonesia	1996, 2002
	Li F, Zhou Y, Li S, Jiang F, Jin X, Yan C, Tian Y, Zhang Y, Tong S, Shen X. Prevalence and risk factors of childhood allergic diseases in eight metropolitan cities in China: a multicenter study.	Inner Mongolia	2005

© 2017 Karimkhani C et al. *JAMA Dermatology.*

	BMC Public Health. 2011; 437.		
	Karimi M, Mirzaei M, Baghiani Moghadam B, Fotouhi E, Zare Mehrjardi A. Pet exposure and the symptoms of asthma, allergic rhinitis and eczema in 6-7 years old children. Iran J Allergy Asthma Immunol. 2011; 10(2): 123-7.	Iran	2009
	Odhiambo JA, Williams HC, Clayton TO, Robertson CF, Asher MI, ISAAC Phase Three Study Group. Global variations in prevalence of eczema symptoms in children from ISAAC Phase Three. J Allergy Clin Immunol. 2009; 124(6): 1251-1258.	Iran	1996, 2001-2002
	Rad MHR, Hamzezadeh A. Allergic disease in 6-7-year-old schoolchildren in Urmia, Islamic Republic of Iran. East Mediterr Health J. 2008; 14(5): 1044-53.	Iran	2002-2003
	Williams H, Stewart A, Von Mutius E, Cookson W, Anderson HR. Is eczema really on the increase worldwide. J Allergy Clin Immunol. 2008; 121(4): 947-954.	Iran	1995, 2001-2002
	Al-Rubiay KK, Al-Rubaiy LK. Dermatoepidemiology: A Household Survey Among Two Urban Areas In Basrah City, Iraq. Internet J Dermatol. 2006; 4(2): 10.	Iraq	2005
	Duggan EM, Sturley J, Fitzgerald AP, Perry IJ, Hourihane JO. The 2002-2007 trends of prevalence of asthma, allergic rhinitis and eczema in Irish schoolchildren. Pediatr Allergy Immunol. 2012; 23(5): 464-71.	Ireland	2002, 2007
	Kabir Z, Manning PJ, Holohan J, Goodman PG, Clancy L. Prevalence of symptoms of severe asthma and allergies in Irish school children: an ISAAC protocol study, 1995-2007. Int J Environ Res Public Health. 2011; 8(8): 3192-201.	Ireland	2007
	Odhiambo JA, Williams HC, Clayton TO, Robertson CF, Asher MI, ISAAC Phase Three Study Group. Global variations in prevalence of eczema symptoms in children from ISAAC Phase Three. J Allergy Clin Immunol. 2009; 124(6): 1251-1258.	Ireland	2003
	Williams H, Stewart A, Von Mutius E, Cookson W, Anderson HR. Is eczema really on the increase worldwide. J Allergy Clin Immunol. 2008; 121(4): 947-954.	Ireland	1995, 2003
	Graif Y, Romano-Zelekha O, Livne I, Green MS, Shohat T. Increased rate and greater severity of	Israel	2003

© 2017 Karimkhani C et al. *JAMA Dermatology.*

	allergic reactions to insect sting among schoolchildren with atopic diseases. Pediatr Allergy Immunol. 2009; 20(8): 757-62.		
	Brescianini S, Brunetto B, Iacovacci P, D'Ippolito C, Alberti G, Schirru MA, Stazi MA, Pini C, Di Felice G, Barletta B. Prevalence of self-perceived allergic diseases and risk factors in Italian adolescents. Pediatr Allergy Immunol. 2009; 20(6): 578-84.	Italy	2003-2004
	Cibella F, Cuttitta G, La Grutta S, Melis MR, Lospalluti ML, Uasuf CG, Bucchieri S, Viegi G. Proportional Venn diagram and determinants of allergic respiratory diseases in Italian adolescents. Pediatr Allergy Immunol. 2011; 22(1 Pt 1): 60-8.	Italy	2005-2006
	Flohr C, Weiland SK, Weinmayr G, Björkstén B, Bråbäck L, Brunekreef B, Büchele G, Clausen M, Cookson WOC, von Mutius E, Strachan DP, Williams HC, ISAAC Phase Two Study Group. The role of atopic sensitization in flexural eczema: findings from the International Study of Asthma and Allergies in Childhood Phase Two. J Allergy Clin Immunol. 2008; 121(1): 141-147.	Italy	2005-2007
	Flohr C, Weinmayr G, Weiland SK, Addo-Yobo E, Annesi-Maesano I, Björkstén B, Bråbäck L, Büchele G, Chico M, Cooper P, Clausen M, El Sharif N, Martinez Gimeno A, Mathur RS, von Mutius E, Morales Suarez-Varela M, Pearce N, Svabe V, Wong GWK, Yu M, Zhong NS, Williams HC, ISAAC Phase Two Study Group. How well do questionnaires perform compared with physical examination in detecting flexural eczema? Findings from the International Study of Asthma and Allergies in Childhood (ISAAC) Phase Two. Br J Dermatol. 2009; 161(4): 846-53.	Italy	2005-2007
	Marcon A, Cazzoletti L, Rava M, Gisondi P, Pironi V, Ricci P, de Marco R. Incidence of respiratory and allergic symptoms in Italian and immigrant children. Respir Med. 2011; 105(2): 204-10.	Italy	2006
	Naldi L, Colombo P, Placchesi EB, Piccitto R, Chatenoud L, La Vecchia C. Study design and preliminary results from the pilot phase of the PraKtis study: self-reported diagnoses of selected skin diseases in a representative sample of the Italian population. Dermatology (Basel). 2004; 208(1): 38-42.	Italy	2003
	Odhiambo JA, Williams HC, Clayton TO,	Italy	2002

© 2017 Karimkhani C et al. *JAMA Dermatology.*

	Robertson CF, Asher MI, ISAAC Phase Three Study Group. Global variations in prevalence of eczema symptoms in children from ISAAC Phase Three. J Allergy Clin Immunol. 2009; 124(6): 1251-1258.		
	Peroni DG, Piacentini GL, Bodini A, Rigotti E, Pigozzi R, Boner AL. Prevalence and risk factors for atopic dermatitis in preschool children. Br J Dermatol. 2008; 158(3): 539-43.	Italy	2005-2007
	Williams H, Stewart A, Von Mutius E, Cookson W, Anderson HR. Is eczema really on the increase worldwide. J Allergy Clin Immunol. 2008; 121(4): 947-954.	Italy	1994-1995, 2002
	Kurosaka F, Terada T, Tanaka A, Nakatani Y, Yamada K, Nishikawa J, Oka K, Takahashi H, Mogami A, Yamada T, Nakano T, Shima M, Nishio H. Risk factors for wheezing, eczema and rhinoconjunctivitis in the previous 12 months among six-year-old children in Himeji City, Japan: food allergy, older siblings, day-care attendance and parental allergy history. Allergol Int. 2011; 60(3): 317-30.	Japan	2005-2006
	Ohfuji S, Miyake Y, Arakawa M, Tanaka K, Sasaki S. Sibship size and prevalence of allergic disorders in Japan: the Ryukyus Child Health Study. Pediatr Allergy Immunol. 2009; 20(4): 377-84.	Japan	2004-2005
	Saeki H, Oiso N, Honma M, Odajima H, Iizuka H, Kawada A, Tamaki K. Comparison of prevalence of atopic dermatitis in Japanese elementary schoolchildren between 2001/2002 and 2007/2008. J Dermatol. 2009; 36(9): 512-4.	Japan	2001-2002, 2007-2008
	Tanaka K, Miyake Y, Arakawa M, Sasaki S, Ohya Y. Dental caries and allergic disorders in Japanese children: the Ryukyus Child Health Study. J Asthma. 2008; 45(9): 795-9.	Japan	2004-2005
	Tanaka K, Miyake Y. Association between prenatal and postnatal tobacco smoke exposure and allergies in young children. J Asthma. 2011; 48(5): 458-63.	Japan	2006-2007
	Odhiambo JA, Williams HC, Clayton TO, Robertson CF, Asher MI, ISAAC Phase Three Study Group. Global variations in prevalence of eczema symptoms in children from ISAAC Phase Three. J Allergy Clin Immunol. 2009; 124(6): 1251-1258.	Jordan	2001
	Odhiambo JA, Williams HC, Clayton TO, Robertson CF, Asher MI, ISAAC Phase Three	Kazakhstan	2002-2003

© 2017 Karimkhani C et al. *JAMA Dermatology.*

	Study Group. Global variations in prevalence of eczema symptoms in children from ISAAC Phase Three. J Allergy Clin Immunol. 2009; 124(6): 1251-1258.		
	Odhiambo JA, Williams HC, Clayton TO, Robertson CF, Asher MI, ISAAC Phase Three Study Group. Global variations in prevalence of eczema symptoms in children from ISAAC Phase Three. J Allergy Clin Immunol. 2009; 124(6): 1251-1258.	Kenya	2001
	Schmeller MD W. Community health workers reduce skin diseases in East African children. Int J Dermatol. 1998; 37(5): 370-7.	Kenya	1993, 1995
	Williams H, Stewart A, Von Mutius E, Cookson W, Anderson HR. Is eczema really on the increase worldwide. J Allergy Clin Immunol. 2008; 121(4): 947-954.	Kenya	1995, 2001
	Odhiambo JA, Williams HC, Clayton TO, Robertson CF, Asher MI, ISAAC Phase Three Study Group. Global variations in prevalence of eczema symptoms in children from ISAAC Phase Three. J Allergy Clin Immunol. 2009; 124(6): 1251-1258.	Kuwait	2001
	Owayed A, Behbehani N, Al-Momen J. Changing prevalence of asthma and allergic diseases among Kuwaiti children. An ISAAC Study (Phase III). Med Princ Pract. 2008; 17(4): 284-9.	Kuwait	2001-2002
	Williams H, Stewart A, Von Mutius E, Cookson W, Anderson HR. Is eczema really on the increase worldwide. J Allergy Clin Immunol. 2008; 121(4): 947-954.	Kuwait	1995, 2001
	Flohr C, Weiland SK, Weinmayr G, Björkstén B, Bråbäck L, Brunekreef B, Büchele G, Clausen M, Cookson WOC, von Mutius E, Strachan DP, Williams HC, ISAAC Phase Two Study Group. The role of atopic sensitization in flexural eczema: findings from the International Study of Asthma and Allergies in Childhood Phase Two. J Allergy Clin Immunol. 2008; 121(1): 141-147.	Latvia	2005-2007
	Flohr C, Weinmayr G, Weiland SK, Addo-Yobo E, Annesi-Maesano I, Björkstén B, Bråbäck L, Büchele G, Chico M, Cooper P, Clausen M, El Sharif N, Martinez Gimeno A, Mathur RS, von Mutius E, Morales Suarez-Varela M, Pearce N, Svabe V, Wong GWK, Yu M, Zhong NS, Williams HC, ISAAC Phase Two Study Group. How well do	Latvia	2005-2007

© 2017 Karimkhani C et al. *JAMA Dermatology.*

	questionnaires perform compared with physical examination in detecting flexural eczema? Findings from the International Study of Asthma and Allergies in Childhood (ISAAC) Phase Two. Br J Dermatol. 2009; 161(4): 846-53.		
	Odhiambo JA, Williams HC, Clayton TO, Robertson CF, Asher MI, ISAAC Phase Three Study Group. Global variations in prevalence of eczema symptoms in children from ISAAC Phase Three. J Allergy Clin Immunol. 2009; 124(6): 1251-1258.	Latvia	2004
	Williams H, Stewart A, Von Mutius E, Cookson W, Anderson HR. Is eczema really on the increase worldwide. J Allergy Clin Immunol. 2008; 121(4): 947-954.	Latvia	1994, 2004
	Al-Sahab B, Atoui M, Musharrafieh U, Zaitoun F, Ramadan F, Tamim H. Epidemiology of eczema among Lebanese adolescents. Int J Public Health. 2008; 53(5): 260-7.	Lebanon	2005
	Musharrafieh U, Al-Sahab B, Zaitoun F, El-Hajj MA, Ramadan F, Tamim H. Prevalence of asthma, allergic rhinitis and eczema among Lebanese adolescents. J Asthma. 2009; 46(4): 382-7.	Lebanon	2005
	Waked M, Salameh P. Asthma, allergic rhinitis and eczema in 5-12-year-old school children across Lebanon. Public Health. 2008; 122(9): 965-73.	Lebanon	2005
	Odhiambo JA, Williams HC, Clayton TO, Robertson CF, Asher MI, ISAAC Phase Three Study Group. Global variations in prevalence of eczema symptoms in children from ISAAC Phase Three. J Allergy Clin Immunol. 2009; 124(6): 1251-1258.	Lithuania	1997, 2001-2002
	Williams H, Stewart A, Von Mutius E, Cookson W, Anderson HR. Is eczema really on the increase worldwide. J Allergy Clin Immunol. 2008; 121(4): 947-954.	Lithuania	1995, 2001-2002
	Odhiambo JA, Williams HC, Clayton TO, Robertson CF, Asher MI, ISAAC Phase Three Study Group. Global variations in prevalence of eczema symptoms in children from ISAAC Phase Three. J Allergy Clin Immunol. 2009; 124(6): 1251-1258.	Macedonia	2002
	Vlaski E, Stavric K, Seckova L, Kimovska M, Isjanovska R. Do household tobacco smoking habits influence asthma, rhinitis and eczema among 13-14 year-old adolescents?. Allergol Immunopathol	Macedonia	2001-2002

© 2017 Karimkhani C et al. *JAMA Dermatology.*

	(Madr). 2011; 39(1): 39-44.		
	Odhiambo JA, Williams HC, Clayton TO, Robertson CF, Asher MI, ISAAC Phase Three Study Group. Global variations in prevalence of eczema symptoms in children from ISAAC Phase Three. J Allergy Clin Immunol. 2009; 124(6): 1251-1258.	Malaysia	2001-2002
	Williams H, Stewart A, Von Mutius E, Cookson W, Anderson HR. Is eczema really on the increase worldwide. J Allergy Clin Immunol. 2008; 121(4): 947-954.	Malaysia	1995, 2001-2002
	Mahé A, Prual A, Konaté M, Bobin P. Skin diseases of children in Mali: a public health problem. Trans R Soc Trop Med Hyg. 1995; 89(5): 467-70.	Mali	1993-1994
	Montefort S, Ellul P, Montefort M, Caruana S, Agius Muscat H. A decrease in the prevalence and improved control of allergic conditions in 13- to 15-yr-old Maltese children (ISAAC). Pediatr Allergy Immunol. 2011; 22(1 Pt 2): e107-111.	Malta	1995, 2002
	Montefort S, Ellul P, Montefort M, Caruana S, Agius Muscat H. Increasing prevalence of asthma, allergic rhinitis but not eczema in 5- to 8-yr-old Maltese children (ISAAC). Pediatr Allergy Immunol. 2009; 20(1): 67-71.	Malta	1994, 2001
	Odhiambo JA, Williams HC, Clayton TO, Robertson CF, Asher MI, ISAAC Phase Three Study Group. Global variations in prevalence of eczema symptoms in children from ISAAC Phase Three. J Allergy Clin Immunol. 2009; 124(6): 1251-1258.	Malta	2001-2002
	Williams H, Stewart A, Von Mutius E, Cookson W, Anderson HR. Is eczema really on the increase worldwide. J Allergy Clin Immunol. 2008; 121(4): 947-954.	Malta	1994-1995, 2001-2002
	Becerril Angeles M, Vázquez Merino CL, Angeles Garay U, Alvarado Moctezuma LE, Vilchis Guízar E. Prevalence of allergic diseases in the elderly. Rev Alerg Mex. 2008; 55(3): 85-91.	Mexico	2006-2008
	Estrada Castañón R, Torres Bibiano B, Alarcón Hernández H, Villegas Arrizón A, Martínez Sandoval E, Chávez López G, Andersson N. Epidemiología cutánea en dos sectores de atención médica en Guerrero, México; Cutaneous epidemiology in two sectors of Guerrero, Mexico. Dermatol rev mex. 1992; 36(1): 29-34.	Mexico	1989-1991
	Williams H, Stewart A, Von Mutius E, Cookson W,	Mexico	1994,

© 2017 Karimkhani C et al. *JAMA Dermatology.*

	Anderson HR. Is eczema really on the increase worldwide. J Allergy Clin Immunol. 2008; 121(4): 947-954.		2002
	Odhiambo JA, Williams HC, Clayton TO, Robertson CF, Asher MI, ISAAC Phase Three Study Group. Global variations in prevalence of eczema symptoms in children from ISAAC Phase Three. J Allergy Clin Immunol. 2009; 124(6): 1251-1258.	Mexico	2002
	Solé D, Mallol J, Wandalsen GF, Aguirre V, Latin American ISAAC Phase 3 Study Group. Prevalence of symptoms of eczema in Latin America: results of the International Study of Asthma and Allergies in Childhood (ISAAC) Phase 3. J Investig Allergol Clin Immunol. 2010; 20(4): 311-23.	Mexico	2001-2003
	Odhiambo JA, Williams HC, Clayton TO, Robertson CF, Asher MI, ISAAC Phase Three Study Group. Global variations in prevalence of eczema symptoms in children from ISAAC Phase Three. J Allergy Clin Immunol. 2009; 124(6): 1251-1258.	Morelos	2002
	Solé D, Mallol J, Wandalsen GF, Aguirre V, Latin American ISAAC Phase 3 Study Group. Prevalence of symptoms of eczema in Latin America: results of the International Study of Asthma and Allergies in Childhood (ISAAC) Phase 3. J Investig Allergol Clin Immunol. 2010; 20(4): 311-23.	Morelos	2001-2003
	Williams H, Stewart A, Von Mutius E, Cookson W, Anderson HR. Is eczema really on the increase worldwide. J Allergy Clin Immunol. 2008; 121(4): 947-954.	Morelos	1994, 2002
	Odhiambo JA, Williams HC, Clayton TO, Robertson CF, Asher MI, ISAAC Phase Three Study Group. Global variations in prevalence of eczema symptoms in children from ISAAC Phase Three. J Allergy Clin Immunol. 2009; 124(6): 1251-1258.	Morocco	1991, 2001-2002
	Williams H, Stewart A, Von Mutius E, Cookson W, Anderson HR. Is eczema really on the increase worldwide. J Allergy Clin Immunol. 2008; 121(4): 947-954.	Morocco	1995, 2001-2002
	Mavale-Manuel S, Joaquim O, Macome C, Almeida L, Nunes E, Daniel A, Malichocho J, Pedro A, Bandeira S, Eduardo E, Maciel L, Constance E, Marques S, Tembe A, de Blic J, Annesi-Maesano I. Asthma and allergies in schoolchildren of Maputo.	Mozambique	2004

© 2017 Karimkhani C et al. *JAMA Dermatology.*

	Allergy. 2007; 62(3): 265-71.		
	Walker SL, Shah M, Hubbard VG, Pradhan HM, Ghimire M. Skin disease is common in rural Nepal: results of a point prevalence study. Br J Dermatol. 2008; 158(2): 334-8.	Nepal	2005-2006
	Flohr C, Weiland SK, Weinmayr G, Björkstén B, Bråbäck L, Brunekreef B, Büchele G, Clausen M, Cookson WOC, von Mutius E, Strachan DP, Williams HC, ISAAC Phase Two Study Group. The role of atopic sensitization in flexural eczema: findings from the International Study of Asthma and Allergies in Childhood Phase Two. J Allergy Clin Immunol. 2008; 121(1): 141-147.	Netherlands	2005-2007
	Flohr C, Weinmayr G, Weiland SK, Addo-Yobo E, Annesi-Maesano I, Björkstén B, Bråbäck L, Büchele G, Chico M, Cooper P, Clausen M, El Sharif N, Martinez Gimeno A, Mathur RS, von Mutius E, Morales Suarez-Varela M, Pearce N, Svabe V, Wong GWK, Yu M, Zhong NS, Williams HC, ISAAC Phase Two Study Group. How well do questionnaires perform compared with physical examination in detecting flexural eczema? Findings from the International Study of Asthma and Allergies in Childhood (ISAAC) Phase Two. Br J Dermatol. 2009; 161(4): 846-53.	Netherlands	2005-2007
	Gabriele C, Silva LM, Arends LR, Raat H, Moll HA, Hofman A, Jaddoe VW, de Jongste JC. Early respiratory morbidity in a multicultural birth cohort: the Generation R Study. Eur J Epidemiol. 2012; 27(6): 453-62.	Netherlands	2002-2006
	Lebon A, Labout JAM, Verbrugh HA, Jaddoe VWV, Hofman A, van Wamel WJB, van Belkum A, Moll HA. Role of Staphylococcus aureus nasal colonization in atopic dermatitis in infants: the Generation R Study. Arch Pediatr Adolesc Med. 2009; 163(8): 745-9.	Netherlands	2004-2007
	Odhiambo JA, Williams HC, Clayton TO, Robertson CF, Asher MI, ISAAC Phase Three Study Group. Global variations in prevalence of eczema symptoms in children from ISAAC Phase Three. J Allergy Clin Immunol. 2009; 124(6): 1251-1258.	Netherlands	2003
	Smit HA, Burdorf A, Coenraads PJ. Prevalence of hand dermatitis in different occupations. Int J Epidemiol. 1993; 22(2): 288-93.	Netherlands	1992
	Visser CAN, Garcia-Marcos L, Eggink J, Brand	Netherlands	2005-

© 2017 Karimkhani C et al. *JAMA Dermatology.*

	PLP. Prevalence and risk factors of wheeze in Dutch infants in their first year of life. Pediatr Pulmonol. 2010; 45(2): 149-56.		2007
	Flohr C, Weiland SK, Weinmayr G, Björkstén B, Bråbäck L, Brunekreef B, Büchele G, Clausen M, Cookson WOC, von Mutius E, Strachan DP, Williams HC, ISAAC Phase Two Study Group. The role of atopic sensitization in flexural eczema: findings from the International Study of Asthma and Allergies in Childhood Phase Two. J Allergy Clin Immunol. 2008; 121(1): 141-147.	New Zealand	2005-2007
	Flohr C, Weinmayr G, Weiland SK, Addo-Yobo E, Annesi-Maesano I, Björkstén B, Bråbäck L, Büchele G, Chico M, Cooper P, Clausen M, El Sharif N, Martinez Gimeno A, Mathur RS, von Mutius E, Morales Suarez-Varela M, Pearce N, Svabe V, Wong GWK, Yu M, Zhong NS, Williams HC, ISAAC Phase Two Study Group. How well do questionnaires perform compared with physical examination in detecting flexural eczema? Findings from the International Study of Asthma and Allergies in Childhood (ISAAC) Phase Two. Br J Dermatol. 2009; 161(4): 846-53.	New Zealand	2005-2007
	Odhiambo JA, Williams HC, Clayton TO, Robertson CF, Asher MI, ISAAC Phase Three Study Group. Global variations in prevalence of eczema symptoms in children from ISAAC Phase Three. J Allergy Clin Immunol. 2009; 124(6): 1251-1258.	New Zealand	2001-2003
	Williams H, Stewart A, Von Mutius E, Cookson W, Anderson HR. Is eczema really on the increase worldwide. J Allergy Clin Immunol. 2008; 121(4): 947-954.	New Zealand	1993, 2001-2003
	Odhiambo JA, Williams HC, Clayton TO, Robertson CF, Asher MI, ISAAC Phase Three Study Group. Global variations in prevalence of eczema symptoms in children from ISAAC Phase Three. J Allergy Clin Immunol. 2009; 124(6): 1251-1258.	Nicaragua	2002
	Solé D, Mallol J, Wandalsen GF, Aguirre V, Latin American ISAAC Phase 3 Study Group. Prevalence of symptoms of eczema in Latin America: results of the International Study of Asthma and Allergies in Childhood (ISAAC) Phase 3. J Investig Allergol Clin Immunol. 2010; 20(4): 311-23.	Nicaragua	2001-2003
	Falade AG, Ige OM, Yusuf BO, Onadeko MO,	Nigeria	1995,

© 2017 Karimkhani C et al. *JAMA Dermatology.*

	Onadeko BO. Trends in the prevalence and severity of symptoms of asthma, allergic rhinoconjunctivitis, and atopic eczema. J Natl Med Assoc. 2009; 101(5): 414-8.		2001-2002
	Odhiambo JA, Williams HC, Clayton TO, Robertson CF, Asher MI, ISAAC Phase Three Study Group. Global variations in prevalence of eczema symptoms in children from ISAAC Phase Three. J Allergy Clin Immunol. 2009; 124(6): 1251-1258.	Nigeria	2001-2002
	Ogunbiyi AO, Omigbodun Y, Owoaje E. Prevalence of skin disorders in school children in southwest Nigeria. Int J Adolesc Med Health. 2009; 21(2): 235-41.	Nigeria	2006-2008
	Okafor OO, Akinbami FO, Orimadegun AE, Okafor CM, Ogunbiyi AO. Prevalence of dermatological lesions in hospitalized children at the University College Hospital, Ibadan, Nigeria. Niger J Clin Pract. 2011; 14(3): 287-92.	Nigeria	2006-2007
	Onayemi O, Isezuo SA, Njoku CH. Prevalence of different skin conditions in an outpatients' setting in north-western Nigeria. Int J Dermatol. 2005; 44(1): 11-Jul.	Nigeria	1999-2001
	Williams H, Stewart A, Von Mutius E, Cookson W, Anderson HR. Is eczema really on the increase worldwide. J Allergy Clin Immunol. 2008; 121(4): 947-954.	Nigeria	1995, 2001-2002
	Yahya H. Change in pattern of skin disease in Kaduna, north-central Nigeria. Int J Dermatol. 2007; 46(9): 936-43.	Nigeria	2000-2005
	Odhiambo JA, Williams HC, Clayton TO, Robertson CF, Asher MI, ISAAC Phase Three Study Group. Global variations in prevalence of eczema symptoms in children from ISAAC Phase Three. J Allergy Clin Immunol. 2009; 124(6): 1251-1258.	North East England	2001
	Williams H, Stewart A, Von Mutius E, Cookson W, Anderson HR. Is eczema really on the increase worldwide. J Allergy Clin Immunol. 2008; 121(4): 947-954.	North East England	1995-1996, 2001
	Flohr C, Weiland SK, Weinmayr G, Björkstén B, Bråbäck L, Brunekreef B, Büchele G, Clausen M, Cookson WOC, von Mutius E, Strachan DP, Williams HC, ISAAC Phase Two Study Group. The role of atopic sensitization in flexural eczema: findings from the International Study of Asthma	Norway	2005-2007

© 2017 Karimkhani C et al. *JAMA Dermatology.*

	and Allergies in Childhood Phase Two. J Allergy Clin Immunol. 2008; 121(1): 141-147.		
	Flohr C, Weinmayr G, Weiland SK, Addo-Yobo E, Annesi-Maesano I, Björkstén B, Bråbäck L, Büchele G, Chico M, Cooper P, Clausen M, El Sharif N, Martinez Gimeno A, Mathur RS, von Mutius E, Morales Suarez-Varela M, Pearce N, Svabe V, Wong GWK, Yu M, Zhong NS, Williams HC, ISAAC Phase Two Study Group. How well do questionnaires perform compared with physical examination in detecting flexural eczema? Findings from the International Study of Asthma and Allergies in Childhood (ISAAC) Phase Two. Br J Dermatol. 2009; 161(4): 846-53.	Norway	2005-2007
	Halvorsen JA, Braae Olesen A, Thoresen M, Holm JØ, Bjertness E, Dalgard F. Comparison of self-reported skin complaints with objective skin signs among adolescents. Acta Derm Venereol. 2008; 88(6): 573-7.	Norway	2006
	Hansen TE, Evjenth B, Holt J. Increasing prevalence of asthma, allergic rhinoconjunctivitis and eczema among schoolchildren: three surveys during the period 1985-2008. Acta Paediatr. 2013; 102(1): 47-52.	Norway	1995, 2008
	Lien L. The association between mental health problems and inflammatory conditions across gender and immigrant status: a population-based cross-sectional study among 10th-grade students. Scand J Public Health. 2008; 36(4): 353-60.	Norway	1999-2001
	Smidesang I, Saunes M, Storrø O, Øien T, Holmen TL, Johnsen R, Henriksen AH. Atopic dermatitis among 2-year olds; high prevalence, but predominantly mild disease--the PACT study, Norway. Pediatr Dermatol. 2008; 25(1): 13-8.	Norway	2003-2005
	Odhiambo JA, Williams HC, Clayton TO, Robertson CF, Asher MI, ISAAC Phase Three Study Group. Global variations in prevalence of eczema symptoms in children from ISAAC Phase Three. J Allergy Clin Immunol. 2009; 124(6): 1251-1258.	Nuevo Leon	2001
	Foliaki S, Annesi-Measano I, Daniel R, Fakakovikaetau T, Magatongia M, Tuuau-Potoi N, Waqatakirewa L, Cheng S, Pearce N. Prevalence of symptoms of childhood asthma, allergic rhinoconjunctivitis and eczema in the Pacific: the International Study of Asthma and Allergies in	Oceania	1998-2003

© 2017 Karimkhani C et al. *JAMA Dermatology.*

	Childhood (ISAAC). Allergy. 2007; 62(3): 259-64.		
	Odhiambo JA, Williams HC, Clayton TO, Robertson CF, Asher MI, ISAAC Phase Three Study Group. Global variations in prevalence of eczema symptoms in children from ISAAC Phase Three. J Allergy Clin Immunol. 2009; 124(6): 1251-1258.	Oman	2001
	Williams H, Stewart A, Von Mutius E, Cookson W, Anderson HR. Is eczema really on the increase worldwide. J Allergy Clin Immunol. 2008; 121(4): 947-954.	Oman	1995, 2001
	Hasnain SM, Khan M, Saleem A, Waqar MA. Prevalence of asthma and allergic rhinitis among school children of Karachi, Pakistan, 2007. J Asthma. 2009; 46(1): 86-90.	Pakistan	2007
	Odhiambo JA, Williams HC, Clayton TO, Robertson CF, Asher MI, ISAAC Phase Three Study Group. Global variations in prevalence of eczema symptoms in children from ISAAC Phase Three. J Allergy Clin Immunol. 2009; 124(6): 1251-1258.	Pakistan	2001-2002
	Williams H, Stewart A, Von Mutius E, Cookson W, Anderson HR. Is eczema really on the increase worldwide. J Allergy Clin Immunol. 2008; 121(4): 947-954.	Pakistan	1995, 2001
	Flohr C, Weiland SK, Weinmayr G, Björkstén B, Bråbäck L, Brunekreef B, Büchele G, Clausen M, Cookson WOC, von Mutius E, Strachan DP, Williams HC, ISAAC Phase Two Study Group. The role of atopic sensitization in flexural eczema: findings from the International Study of Asthma and Allergies in Childhood Phase Two. J Allergy Clin Immunol. 2008; 121(1): 141-147.	Palestine	2005-2007
	Odhiambo JA, Williams HC, Clayton TO, Robertson CF, Asher MI, ISAAC Phase Three Study Group. Global variations in prevalence of eczema symptoms in children from ISAAC Phase Three. J Allergy Clin Immunol. 2009; 124(6): 1251-1258.	Palestine	2000
	Odhiambo JA, Williams HC, Clayton TO, Robertson CF, Asher MI, ISAAC Phase Three Study Group. Global variations in prevalence of eczema symptoms in children from ISAAC Phase Three. J Allergy Clin Immunol. 2009; 124(6): 1251-1258.	Panama	2001
	Solé D, Mallol J, Wandalsen GF, Aguirre V, Latin	Panama	2001-

	American ISAAC Phase 3 Study Group. Prevalence of symptoms of eczema in Latin America: results of the International Study of Asthma and Allergies in Childhood (ISAAC) Phase 3. J Investig Allergol Clin Immunol. 2010; 20(4): 311-23.		2003
	Williams H, Stewart A, Von Mutius E, Cookson W, Anderson HR. Is eczema really on the increase worldwide. J Allergy Clin Immunol. 2008; 121(4): 947-954.	Panama	1995, 2001
	Odhiambo JA, Williams HC, Clayton TO, Robertson CF, Asher MI, ISAAC Phase Three Study Group. Global variations in prevalence of eczema symptoms in children from ISAAC Phase Three. J Allergy Clin Immunol. 2009; 124(6): 1251-1258.	Paraguay	2002
	Solé D, Mallol J, Wandalsen GF, Aguirre V, Latin American ISAAC Phase 3 Study Group. Prevalence of symptoms of eczema in Latin America: results of the International Study of Asthma and Allergies in Childhood (ISAAC) Phase 3. J Investig Allergol Clin Immunol. 2010; 20(4): 311-23.	Paraguay	2001-2003
	Williams H, Stewart A, Von Mutius E, Cookson W, Anderson HR. Is eczema really on the increase worldwide. J Allergy Clin Immunol. 2008; 121(4): 947-954.	Paraguay	1997, 2002
	Gutierrez EL, Galarza C, Ramos W. Prevalencia de Enfermedades Dermatológicas en una comunidad rural de Ucayali, Perú. Dermatol Peru. 2009; 19(2): 104-13.	Peru	2005
	Odhiambo JA, Williams HC, Clayton TO, Robertson CF, Asher MI, ISAAC Phase Three Study Group. Global variations in prevalence of eczema symptoms in children from ISAAC Phase Three. J Allergy Clin Immunol. 2009; 124(6): 1251-1258.	Peru	2001
	Solé D, Mallol J, Wandalsen GF, Aguirre V, Latin American ISAAC Phase 3 Study Group. Prevalence of symptoms of eczema in Latin America: results of the International Study of Asthma and Allergies in Childhood (ISAAC) Phase 3. J Investig Allergol Clin Immunol. 2010; 20(4): 311-23.	Peru	2001-2003
	Williams H, Stewart A, Von Mutius E, Cookson W, Anderson HR. Is eczema really on the increase worldwide. J Allergy Clin Immunol. 2008; 121(4): 947-954.	Peru	1995, 2001
	Odhiambo JA, Williams HC, Clayton TO,	Philippines	2001

© 2017 Karimkhani C et al. *JAMA Dermatology.*

	Robertson CF, Asher MI, ISAAC Phase Three Study Group. Global variations in prevalence of eczema symptoms in children from ISAAC Phase Three. J Allergy Clin Immunol. 2009; 124(6): 1251-1258.		
	Williams H, Stewart A, Von Mutius E, Cookson W, Anderson HR. Is eczema really on the increase worldwide. J Allergy Clin Immunol. 2008; 121(4): 947-954.	Philippines	1994, 2001
	Odhiambo JA, Williams HC, Clayton TO, Robertson CF, Asher MI, ISAAC Phase Three Study Group. Global variations in prevalence of eczema symptoms in children from ISAAC Phase Three. J Allergy Clin Immunol. 2009; 124(6): 1251-1258.	Poland	2001-2002
	Williams H, Stewart A, Von Mutius E, Cookson W, Anderson HR. Is eczema really on the increase worldwide. J Allergy Clin Immunol. 2008; 121(4): 947-954.	Poland	1994-1995, 2002
	Massa A, Alves R, Amado J, Matos E, Sanches M, Selores M, Santos C, Costa V, Velho G, Oliveira M, Ferreira E, Taveira M, Silva NS, Granado E, Lemos A, Calheiros JM. Prevalence of cutaneous lesions in Freixo de Espada à Cinta. Acta Med Port. 2000; 13(5-6): 247-54.	Portugal	1994
	Odhiambo JA, Williams HC, Clayton TO, Robertson CF, Asher MI, ISAAC Phase Three Study Group. Global variations in prevalence of eczema symptoms in children from ISAAC Phase Three. J Allergy Clin Immunol. 2009; 124(6): 1251-1258.	Portugal	2001-2002
	Williams H, Stewart A, Von Mutius E, Cookson W, Anderson HR. Is eczema really on the increase worldwide. J Allergy Clin Immunol. 2008; 121(4): 947-954.	Portugal	1993-1995, 2001-2002
	Odhiambo JA, Williams HC, Clayton TO, Robertson CF, Asher MI, ISAAC Phase Three Study Group. Global variations in prevalence of eczema symptoms in children from ISAAC Phase Three. J Allergy Clin Immunol. 2009; 124(6): 1251-1258.	Romania	2001
	Popescu R, Popescu CM, Williams HC, Forsea D. The prevalence of skin conditions in Romanian school children. Br J Dermatol. 1999; 140(5): 891-6.	Romania	1995
	Williams H, Stewart A, Von Mutius E, Cookson W,	Romania	1994,

© 2017 Karimkhani C et al. *JAMA Dermatology.*

	Anderson HR. Is eczema really on the increase worldwide. J Allergy Clin Immunol. 2008; 121(4): 947-954.		2001
	Hugg T, Ruotsalainen R, Jaakkola MS, Pushkarev V, Jaakkola JJK. Comparison of allergic diseases, symptoms and respiratory infections between Finnish and Russian school children. Eur J Epidemiol. 2008; 23(2): 123-33.	Russia	1996, 2003, 2005
	Odhiambo JA, Williams HC, Clayton TO, Robertson CF, Asher MI, ISAAC Phase Three Study Group. Global variations in prevalence of eczema symptoms in children from ISAAC Phase Three. J Allergy Clin Immunol. 2009; 124(6): 1251-1258.	Russia	2002
	Williams H, Stewart A, Von Mutius E, Cookson W, Anderson HR. Is eczema really on the increase worldwide. J Allergy Clin Immunol. 2008; 121(4): 947-954.	Russia	1996, 2002, 2005
	Hogewoning A, Amoah A, Bavinck JNB, Boakye D, Yazdanbakhsh M, Adegnika A, De Smedt S, Fonteyne Y, Willemze R, Lavrijsen A. Skin diseases among schoolchildren in Ghana, Gabon, and Rwanda. Int J Dermatol. 2013; 52(5): 589-600/	Rwanda	2007
	Hogewoning AA, Bouwes Bavinck JN, Amoah AS, Boakye DA, Yazdanbakhsh M, Kremsner PG, Adegnika AA, De Smedt SKAD, Willemze R, Lavrijsen APM. Point and period prevalences of eczema in rural and urban schoolchildren in Ghana, Gabon and Rwanda. J Eur Acad Dermatol Venereol. 2012; 26(4): 488-94.	Rwanda	2007
	Foliaki S, Annesi-Measano I, Daniel R, Fakakovikaetau T, Magatongia M, Tuuau-Potoi N, Waqatakirewa L, Cheng S, Pearce N. Prevalence of symptoms of childhood asthma, allergic rhinoconjunctivitis and eczema in the Pacific: the International Study of Asthma and Allergies in Childhood (ISAAC). Allergy. 2007; 62(3): 259-64.	Samoa	1998-2003
	Odhiambo JA, Williams HC, Clayton TO, Robertson CF, Asher MI, ISAAC Phase Three Study Group. Global variations in prevalence of eczema symptoms in children from ISAAC Phase Three. J Allergy Clin Immunol. 2009; 124(6): 1251-1258.	Samoa	2003
	Al-Saeed WY, Al-Dawood KM, Bukhari IA, Bahnassy AA. Prevalence and pattern of skin disorders among female schoolchildren in Eastern	Saudi Arabia	2003

© 2017 Karimkhani C et al. *JAMA Dermatology.*

	Saudi Arabia. Saudi Med J. 2006; 27(2): 227-34.		
	Al-Shammari SA, Al-Sheikh O. Skin morbidity pattern among patients seen at a university primary care clinic in Riyadh, Saudi Arabia. Gulf J Dermatol Venereol. 1996; 3: 22-5.	Saudi Arabia	1993-1994
	Bahamdan K, Mahfouz AA, Tallab T, Badawi IA, al-Amari OM. Skin diseases among adolescent boys in Abha, Saudi Arabia. Int J Dermatol. 1996; 35(6): 405-7.	Saudi Arabia	1995-1996
	Nahhas M, Bhopal R, Anandan C, Elton R, Sheikh A. Prevalence of allergic disorders among primary school-aged children in Madinah, Saudi Arabia: two-stage cross-sectional survey. PLoS One. 2012; 7(5): e36848.	Saudi Arabia	2009
	Odhiambo JA, Williams HC, Clayton TO, Robertson CF, Asher MI, ISAAC Phase Three Study Group. Global variations in prevalence of eczema symptoms in children from ISAAC Phase Three. J Allergy Clin Immunol. 2009; 124(6): 1251-1258.	Scotland	2002
	Williams H, Stewart A, Von Mutius E, Cookson W, Anderson HR. Is eczema really on the increase worldwide. J Allergy Clin Immunol. 2008; 121(4): 947-954.	Scotland	1995, 2002
	Odhiambo JA, Williams HC, Clayton TO, Robertson CF, Asher MI, ISAAC Phase Three Study Group. Global variations in prevalence of eczema symptoms in children from ISAAC Phase Three. J Allergy Clin Immunol. 2009; 124(6): 1251-1258.	Serbia	2001-2003
	Li F, Zhou Y, Li S, Jiang F, Jin X, Yan C, Tian Y, Zhang Y, Tong S, Shen X. Prevalence and risk factors of childhood allergic diseases in eight metropolitan cities in China: a multicenter study. BMC Public Health. 2011; 437.	Shaanxi	2005
	Odhiambo JA, Williams HC, Clayton TO, Robertson CF, Asher MI, ISAAC Phase Three Study Group. Global variations in prevalence of eczema symptoms in children from ISAAC Phase Three. J Allergy Clin Immunol. 2009; 124(6): 1251-1258.	Singapore	2001
	Tay Y-K, Kong K-H, Khoo L, Goh C-L, Giam Y-C. The prevalence and descriptive epidemiology of atopic dermatitis in Singapore school children. Br J Dermatol. 2002; 146(1): 101-6.	Singapore	1999
	Wang XS, Shek LP, Ma S, Soh SE, Lee BW, Goh	Singapore	1994,

© 2017 Karimkhani C et al. *JAMA Dermatology.*

	DYT. Time trends of co-existing atopic conditions in Singapore school children: prevalence and related factors. Pediatr Allergy Immunol. 2010; 21(1 Pt 2): e137-141.		2001
	Williams H, Stewart A, Von Mutius E, Cookson W, Anderson HR. Is eczema really on the increase worldwide. J Allergy Clin Immunol. 2008; 121(4): 947-954.	Singapore	1994, 2001
	Chalmers DA, Todd G, Saxe N, Milne JT, Tolosana S, Ngcelwane PN, Hlaba BN, Mngomeni LN, Nonxuba TG, Williams HC. Validation of the U.K. Working Party diagnostic criteria for atopic eczema in a Xhosa-speaking African population. Br J Dermatol. 2007; 156(1): 111-6.	South Africa	2004-2006
	Odhiambo JA, Williams HC, Clayton TO, Robertson CF, Asher MI, ISAAC Phase Three Study Group. Global variations in prevalence of eczema symptoms in children from ISAAC Phase Three. J Allergy Clin Immunol. 2009; 124(6): 1251-1258.	South Africa	2002, 2004
	Wichmann J, Wolvaardt JE, Maritz C, Voyi KVV. Association between children's household living conditions and eczema in the Polokwane area, South Africa. Health Place. 2008; 14(2): 323-35.	South Africa	2004-2005
	Williams H, Stewart A, Von Mutius E, Cookson W, Anderson HR. Is eczema really on the increase worldwide. J Allergy Clin Immunol. 2008; 121(4): 947-954.	South Africa	1995, 2002
	Zar HJ, Ehrlich RI, Workman L, Weinberg EG. The changing prevalence of asthma, allergic rhinitis and atopic eczema in African adolescents from 1995 to 2002. Pediatr Allergy Immunol. 2007; 18(7): 560-5.	South Africa	1995, 2002
	Odhiambo JA, Williams HC, Clayton TO, Robertson CF, Asher MI, ISAAC Phase Three Study Group. Global variations in prevalence of eczema symptoms in children from ISAAC Phase Three. J Allergy Clin Immunol. 2009; 124(6): 1251-1258.	South East England	2002
	Williams H, Stewart A, Von Mutius E, Cookson W, Anderson HR. Is eczema really on the increase worldwide. J Allergy Clin Immunol. 2008; 121(4): 947-954.	South East England	1992, 2002
	Choi WJ, Ko JY, Kim JW, Lee KH, Park CW, Kim KH, Kim MN, Lee AY, Cho SH, Park YL, Choi JH, Seo SJ, Lee YW, Roh JY, Park YM, Kim DJ, Ro YS. Prevalence and risk factors for atopic	South Korea	2008

	dermatitis: a cross-sectional study of 6,453 Korean preschool children. Acta Derm Venereol. 2012; 92(5): 467-71.		
	Lee J-Y, Seo J-H, Kwon J-W, Yu J, Kim B-J, Lee S-Y, Kim H-B, Kim W-K, Kim K-W, Shin Y-J, Hong S-J. Exposure to gene-environment interactions before 1 year of age may favor the development of atopic dermatitis. Int Arch Allergy Immunol. 2012; 157(4): 363-71.	South Korea	2008
	Odhiambo JA, Williams HC, Clayton TO, Robertson CF, Asher MI, ISAAC Phase Three Study Group. Global variations in prevalence of eczema symptoms in children from ISAAC Phase Three. J Allergy Clin Immunol. 2009; 124(6): 1251-1258.	South Korea	2000
	Suh M, Kim H-H, Sohn MH, Kim K-E, Kim C, Shin DC. Prevalence of allergic diseases among Korean school-age children: a nationwide cross-sectional questionnaire study. J Korean Med Sci. 2011; 26(3): 332-8.	South Korea	2006
	Williams H, Stewart A, Von Mutius E, Cookson W, Anderson HR. Is eczema really on the increase worldwide. J Allergy Clin Immunol. 2008; 121(4): 947-954.	South Korea	1995, 2000
	Flohr C, Weiland SK, Weinmayr G, Björkstén B, Bråbäck L, Brunekreef B, Büchele G, Clausen M, Cookson WOC, von Mutius E, Strachan DP, Williams HC, ISAAC Phase Two Study Group. The role of atopic sensitization in flexural eczema: findings from the International Study of Asthma and Allergies in Childhood Phase Two. J Allergy Clin Immunol. 2008; 121(1): 141-147.	South West England	2005-2007
	Flohr C, Weinmayr G, Weiland SK, Addo-Yobo E, Annesi-Maesano I, Björkstén B, Bråbäck L, Büchele G, Chico M, Cooper P, Clausen M, El Sharif N, Martinez Gimeno A, Mathur RS, von Mutius E, Morales Suarez-Varela M, Pearce N, Svabe V, Wong GWK, Yu M, Zhong NS, Williams HC, ISAAC Phase Two Study Group. How well do questionnaires perform compared with physical examination in detecting flexural eczema? Findings from the International Study of Asthma and Allergies in Childhood (ISAAC) Phase Two. Br J Dermatol. 2009; 161(4): 846-53.	South West England	2005-2007
	Ziyab AH, Raza A, Karmaus W, Tongue N, Zhang H, Matthews S, Arshad SH, Roberts G. Trends in	South West England	1989-1990,

	eczema in the first 18 years of life: results from the Isle of Wight 1989 birth cohort study. Clin Exp Allergy. 2010; 40(12): 1776-84.		1993, 2007
	Arnedo-Pena A, Puig-Barberà J, Bellido-Blasco J-B, Pac-Sa MR, Campos-Cruañes J-B, Artero-Sivera A, Museros-Recatalá L. Risk factors and prevalence of asthma in schoolchildren in Castellon (Spain): a cross-sectional study. Allergol Immunopathol (Madr). 2009; 37(3): 135-42.	Spain	2002
	Batllés-Garrido J, Torres-Borrego J, Rubí-Ruiz T, Bonillo-Perales A, González-Jiménez Y, Momblán De Cabo J, Aguirre-Rodríguez J, Losillas-Maldonado A, Torres-Daza M. Prevalence and factors linked to atopy in 10-and 11-year-old children in Almería, Spain. Allergol Immunopathol (Madr). 2010; 38(1): 13-9.	Spain	2007-2009
	Flohr C, Weiland SK, Weinmayr G, Björkstén B, Bråbäck L, Brunekreef B, Büchele G, Clausen M, Cookson WOC, von Mutius E, Strachan DP, Williams HC, ISAAC Phase Two Study Group. The role of atopic sensitization in flexural eczema: findings from the International Study of Asthma and Allergies in Childhood Phase Two. J Allergy Clin Immunol. 2008; 121(1): 141-147.	Spain	2005-2007
	Flohr C, Weinmayr G, Weiland SK, Addo-Yobo E, Annesi-Maesano I, Björkstén B, Bråbäck L, Büchele G, Chico M, Cooper P, Clausen M, El Sharif N, Martinez Gimeno A, Mathur RS, von Mutius E, Morales Suarez-Varela M, Pearce N, Svabe V, Wong GWK, Yu M, Zhong NS, Williams HC, ISAAC Phase Two Study Group. How well do questionnaires perform compared with physical examination in detecting flexural eczema? Findings from the International Study of Asthma and Allergies in Childhood (ISAAC) Phase Two. Br J Dermatol. 2009; 161(4): 846-53.	Spain	2005-2007
	Font-Ribera L, Kogevinas M, Zock J-P, Nieuwenhuijsen MJ, Heederik D, Villanueva CM. Swimming pool attendance and risk of asthma and allergic symptoms in children. Eur Respir J. 2009; 34(6): 1304-10.	Spain	2006
	Garcia-Marcos L, González-Díaz C, Garvajal-Urueña I, Pac-Sa MR, Busquets-Monge RM, Suárez-Varela MM, Batlles-Garrido J, Blanco-Quirós A, Varela AL-S, García-Hernández G, Aguinaga-Ontoso I. Early exposure to paracetamol	Spain	2007-2009

© 2017 Karimkhani C et al. *JAMA Dermatology.*

	or to antibiotics and eczema at school age: modification by asthma and rhinoconjunctivitis. Pediatr Allergy Immunol. 2010; 21(7): 1036-42.		
	Morales Suárez-Varela M, García-Marcos L, Kogan MD, Llopis González A, Martínez Gimeno A, Aguinaga Ontoso I, González Díaz C, Arnedo Peña A, Domínguez Aurrecoechea B, Busquets Monge RM, Blanco Quirós A, Batlles Garrido J, Miner Canflanca I, López-Silvarrey Várela A, Gimeno Clemente N. Parents' smoking habit and prevalence of atopic eczema in 6-7 and 13-14 year-old schoolchildren in Spain. ISAAC phase III. Allergol Immunopathol (Madr). 2008; 36(6): 336-42.	Spain	2005-2007
	Odhiambo JA, Williams HC, Clayton TO, Robertson CF, Asher MI, ISAAC Phase Three Study Group. Global variations in prevalence of eczema symptoms in children from ISAAC Phase Three. J Allergy Clin Immunol. 2009; 124(6): 1251-1258.	Spain	1996, 2001-2003
	Suárez-Varela MM, Alvarez LG-M, Kogan MD, Ferreira JC, Martínez Gimeno A, Aguinaga Ontoso I, González Díaz C, Arnedo Pena A, Domínguez Aurrecoechea B, Busquets Monge RM, Blanco Quiros A, Batlles Garrido J, García de Andoain N, Varela AL-S, García Merino A, Gimeno Clemente N, Llopis González A. Diet and prevalence of atopic eczema in 6 to 7-year-old schoolchildren in Spain: ISAAC phase III. J Investig Allergol Clin Immunol. 2010; 20(6): 469-75.	Spain	2006-2008
	Suárez-Varela MM, García-Marcos Alvarez L, Kogan MD, González AL, Gimeno AM, Aguinaga Ontoso I, Díaz CG, Pena AA, Aurrecoechea BD, Monge RMB, Quiros AB, Garrido JB, Canflanca IM, Varela AL-S. Climate and prevalence of atopic eczema in 6- to 7-year-old school children in Spain. ISAAC phase III. Int J Biometeorol. 2008; 52(8): 833-40.	Spain	2007
	Williams H, Stewart A, Von Mutius E, Cookson W, Anderson HR. Is eczema really on the increase worldwide. J Allergy Clin Immunol. 2008; 121(4): 947-954.	Spain	1993-1994, 1997, 2001-2002
	Amarasekera NDDM, Gunawardena NK, de Silva NR, Weerasinghe A. Prevalence of childhood atopic diseases in the Western Province of Sri Lanka. Ceylon Med J. 2010; 55(1): 5-8.	Sri Lanka	2006

© 2017 Karimkhani C et al. *JAMA Dermatology.*

	Odhiambo JA, Williams HC, Clayton TO, Robertson CF, Asher MI, ISAAC Phase Three Study Group. Global variations in prevalence of eczema symptoms in children from ISAAC Phase Three. J Allergy Clin Immunol. 2009; 124(6): 1251-1258.	Sri Lanka	2001
	Perera A, Atukorale DN, Sivayogan S, Ariyaratne VS, Karunaratne LDA. Prevalence of skin diseases in suburban Sri Lanka. Ceylon Med J. 2000; 45(3): 123-8.	Sri Lanka	1997
	Odhiambo JA, Williams HC, Clayton TO, Robertson CF, Asher MI, ISAAC Phase Three Study Group. Global variations in prevalence of eczema symptoms in children from ISAAC Phase Three. J Allergy Clin Immunol. 2009; 124(6): 1251-1258.	Sudan	2003
	Ballardini N, Kull I, Söderhäll C, Lilja G, Wickman M, Wahlgren CF. Eczema severity in preadolescent children and its relation to sex, filaggrin mutations, asthma, rhinitis, aggravating factors and topical treatment: a report from the BAMSE birth cohort. Br J Dermatol. 2013; 168(3): 588-94.	Sweden	2008
	Bjerg A, Sandström T, Lundbäck B, Rönmark E. Time trends in asthma and wheeze in Swedish children 1996-2006: prevalence and risk factors by sex. Allergy. 2010; 65(1): 48-55.	Sweden	1996, 2006
	Flohr C, Weiland SK, Weinmayr G, Björkstén B, Bråbäck L, Brunekreef B, Büchele G, Clausen M, Cookson WOC, von Mutius E, Strachan DP, Williams HC, ISAAC Phase Two Study Group. The role of atopic sensitization in flexural eczema: findings from the International Study of Asthma and Allergies in Childhood Phase Two. J Allergy Clin Immunol. 2008; 121(1): 141-147.	Sweden	2005-2007
	Lindberg M, Isacson D, Bingefors K. Self-reported skin diseases, quality of life and medication use: a nationwide pharmaco-epidemiological survey in Sweden. Acta Derm Venereol. 2014; 94(2): 188-91.	Sweden	2004-2005
	Meding B, Järvholm B. Hand eczema in Swedish adults - changes in prevalence between 1983 and 1996. J Invest Dermatol. 2002; 118(4): 719-23.	Sweden	1996
	Odhiambo JA, Williams HC, Clayton TO, Robertson CF, Asher MI, ISAAC Phase Three Study Group. Global variations in prevalence of eczema symptoms in children from ISAAC Phase Three. J Allergy Clin Immunol. 2009; 124(6): 1251-	Sweden	2002

© 2017 Karimkhani C et al. *JAMA Dermatology.*

	1258.		
	Rönmark EP, Ekerljung L, Lo?tvall J, Wennergren G, Ro?nmark E, Tore?n K, Lundba?ck B. Eczema among adults: prevalence, risk factors and relation to airway diseases. Results from a large-scale population survey in Sweden. Br J Dermatol. 2012; 166(6): 1301-08.	Sweden	2008
	Rönmark E, Bjerg A, Perzanowski M, Platts-Mills T, Lundbäck B. Major increase in allergic sensitization in schoolchildren from 1996 to 2006 in northern Sweden. J Allergy Clin Immunol. 2009; 124(2): 357-363.	Sweden	1996, 2006
	Williams H, Stewart A, Von Mutius E, Cookson W, Anderson HR. Is eczema really on the increase worldwide. J Allergy Clin Immunol. 2008; 121(4): 947-954.	Sweden	1994, 2002
	Mohammad Y, Tabbah K, Mohammad S, Yassine F, Clayton T, Hassan M. International study of asthma and allergies in childhood: phase 3 in the Syrian Arab Republic. East Mediterr Health J. 2010; 16(7): 710-6.	Syria	2001-2003
	Odhiambo JA, Williams HC, Clayton TO, Robertson CF, Asher MI, ISAAC Phase Three Study Group. Global variations in prevalence of eczema symptoms in children from ISAAC Phase Three. J Allergy Clin Immunol. 2009; 124(6): 1251-1258.	Syria	2001-2003
	Solé D, Mallol J, Wandalsen GF, Aguirre V, Latin American ISAAC Phase 3 Study Group. Prevalence of symptoms of eczema in Latin America: results of the International Study of Asthma and Allergies in Childhood (ISAAC) Phase 3. J Investig Allergol Clin Immunol. 2010; 20(4): 311-23.	Tabasco	2001-2003
	Hsu N-Y, Wu P-C, Bornehag C-G, Sundell J, Su H-J. Feeding bottles usage and the prevalence of childhood allergy and asthma. Clin Dev Immunol. 2012; 1-8.	Taiwan	2005-2006
	Liao M-F, Liao M-N, Lin S-N, Chen J-Y, Huang J-L. Prevalence of allergic diseases of schoolchildren in central Taiwan. From ISAAC surveys 5 years apart. J Asthma. 2009; 46(6): 541-5.	Taiwan	2002, 2007
	Odhiambo JA, Williams HC, Clayton TO, Robertson CF, Asher MI, ISAAC Phase Three Study Group. Global variations in prevalence of eczema symptoms in children from ISAAC Phase Three. J Allergy Clin Immunol. 2009; 124(6): 1251-	Taiwan	2001-2002

© 2017 Karimkhani C et al. *JAMA Dermatology.*

	1258.		
	Williams H, Stewart A, Von Mutius E, Cookson W, Anderson HR. Is eczema really on the increase worldwide. J Allergy Clin Immunol. 2008; 121(4): 947-954.	Taiwan	1994, 2001-2002
	Wu WF, Wan KS, Wang SJ, Yang W, Liu WL. Prevalence, severity, and time trends of allergic conditions in 6-to-7-year-old schoolchildren in Taipei. J Investig Allergol Clin Immunol. 2011; 21(7): 556-62.	Taiwan	2007
	Yang Y-C, Cheng Y-W, Lai C-S, Chen W. Prevalence of childhood acne, ephelides, warts, atopic dermatitis, psoriasis, alopecia areata and keloid in Kaohsiung County, Taiwan: a community-based clinical survey. J Eur Acad Dermatol Venereol. 2007; 21(5): 643-9.	Taiwan	2004
	Yao T-C, Ou L-S, Yeh K-W, Lee W-I, Chen L-C, Huang J-L, PATCH Study Group. Associations of age, gender, and BMI with prevalence of allergic diseases in children: PATCH study. J Asthma. 2011; 48(5): 503-10.	Taiwan	2007
	Yeh K-W, Ou L-S, Yao T-C, Chen L-C, Lee W-I, Huang J-L, PATCH Study Group. Prevalence and risk factors for early presentation of asthma among preschool children in Taiwan. Asian Pac J Allergy Immunol. 2011; 29(2): 120-6.	Taiwan	2007-2008
	Odhiambo JA, Williams HC, Clayton TO, Robertson CF, Asher MI, ISAAC Phase Three Study Group. Global variations in prevalence of eczema symptoms in children from ISAAC Phase Three. J Allergy Clin Immunol. 2009; 124(6): 1251-1258.	Tamaulipas	2003
	Solé D, Mallol J, Wandalsen GF, Aguirre V, Latin American ISAAC Phase 3 Study Group. Prevalence of symptoms of eczema in Latin America: results of the International Study of Asthma and Allergies in Childhood (ISAAC) Phase 3. J Investig Allergol Clin Immunol. 2010; 20(4): 311-23.	Tamaulipas	2001-2003
	Henderson CA. Skin disease in rural Tanzania. Int J Dermatol. 1996; 35(9): 640-2.	Tanzania	1991
	Komba EV, Mgonda YM. The spectrum of dermatological disorders among primary school children in Dar es Salaam. BMC Public Health. 2010; 10(1): 765.	Tanzania	2007-2010
	Mgonda YM, Chale PNF. The burden of co-existing dermatological disorders and their tendency of	Tanzania	2009-2010

© 2017 Karimkhani C et al. *JAMA Dermatology.*

	being overlooked among patients admitted to Muhimbili National Hospital in Dar es Salaam, Tanzania. BMC Dermatol. 2011; 11: 8.		
	Satimia FT, McBride SR, Leppard B. Prevalence of skin disease in rural Tanzania and factors influencing the choice of health care, modern or traditional. Arch Dermatol. 1998; 134(11): 1363-6.	Tanzania	1995
	Odhiambo JA, Williams HC, Clayton TO, Robertson CF, Asher MI, ISAAC Phase Three Study Group. Global variations in prevalence of eczema symptoms in children from ISAAC Phase Three. J Allergy Clin Immunol. 2009; 124(6): 1251-1258.	Thailand	1995-1996, 1999, 2001
	Williams H, Stewart A, Von Mutius E, Cookson W, Anderson HR. Is eczema really on the increase worldwide. J Allergy Clin Immunol. 2008; 121(4): 947-954.	Thailand	1995, 2001
	Odhiambo JA, Williams HC, Clayton TO, Robertson CF, Asher MI, ISAAC Phase Three Study Group. Global variations in prevalence of eczema symptoms in children from ISAAC Phase Three. J Allergy Clin Immunol. 2009; 124(6): 1251-1258.	Tibet	2001
	Odhiambo JA, Williams HC, Clayton TO, Robertson CF, Asher MI, ISAAC Phase Three Study Group. Global variations in prevalence of eczema symptoms in children from ISAAC Phase Three. J Allergy Clin Immunol. 2009; 124(6): 1251-1258.	Togo	2001
	Foliaki S, Annesi-Measano I, Daniel R, Fakakovikaetau T, Magatongia M, Tuuau-Potoi N, Waqatakirewa L, Cheng S, Pearce N. Prevalence of symptoms of childhood asthma, allergic rhinoconjunctivitis and eczema in the Pacific: the International Study of Asthma and Allergies in Childhood (ISAAC). Allergy. 2007; 62(3): 259-64.	Tonga	1998-2003
	Odhiambo JA, Williams HC, Clayton TO, Robertson CF, Asher MI, ISAAC Phase Three Study Group. Global variations in prevalence of eczema symptoms in children from ISAAC Phase Three. J Allergy Clin Immunol. 2009; 124(6): 1251-1258.	Tonga	2002
	Odhiambo JA, Williams HC, Clayton TO, Robertson CF, Asher MI, ISAAC Phase Three Study Group. Global variations in prevalence of eczema symptoms in children from ISAAC Phase	Trinidad and Tobago	2002

	Three. J Allergy Clin Immunol. 2009; 124(6): 1251-1258.		
	Odhiambo JA, Williams HC, Clayton TO, Robertson CF, Asher MI, ISAAC Phase Three Study Group. Global variations in prevalence of eczema symptoms in children from ISAAC Phase Three. J Allergy Clin Immunol. 2009; 124(6): 1251-1258.	Tunisia	2001
	Williams H, Stewart A, Von Mutius E, Cookson W, Anderson HR. Is eczema really on the increase worldwide. J Allergy Clin Immunol. 2008; 121(4): 947-954.	Tunisia	1996, 2001-2002
	Civelek E, Cakir B, Boz AB, Yuksel H, Orhan F, Uner A, Sekerel BE. Extent and burden of allergic diseases in elementary schoolchildren: a national multicenter study. J Investig Allergol Clin Immunol. 2010; 20(4): 280-8.	Turkey	2005-2006
	Demir AU, Celikel S, Karakaya G, Kalyoncu AF. Asthma and allergic diseases in school children from 1992 to 2007 with incidence data. J Asthma. 2010; 47(10): 1128-35.	Turkey	1992, 1997, 2002, 2007
	Flohr C, Weiland SK, Weinmayr G, Björkstén B, Bråbäck L, Brunekreef B, Büchele G, Clausen M, Cookson WOC, von Mutius E, Strachan DP, Williams HC, ISAAC Phase Two Study Group. The role of atopic sensitization in flexural eczema: findings from the International Study of Asthma and Allergies in Childhood Phase Two. J Allergy Clin Immunol. 2008; 121(1): 141-147.	Turkey	2005-2007
	Flohr C, Weinmayr G, Weiland SK, Addo-Yobo E, Annesi-Maesano I, Björkstén B, Bråbäck L, Büchele G, Chico M, Cooper P, Clausen M, El Sharif N, Martinez Gimeno A, Mathur RS, von Mutius E, Morales Suarez-Varela M, Pearce N, Svabe V, Wong GWK, Yu M, Zhong NS, Williams HC, ISAAC Phase Two Study Group. How well do questionnaires perform compared with physical examination in detecting flexural eczema? Findings from the International Study of Asthma and Allergies in Childhood (ISAAC) Phase Two. Br J Dermatol. 2009; 161(4): 846-53.	Turkey	2005-2007
	Guner SN, Gokturk B, Kilic M, Ozkiraz S. The prevalences of allergic diseases in rural and urban areas are similar. Allergol Immunopathol (Madr). 2011; 39(3): 140-4.	Turkey	2007
	Kurt E, Metintas S, Basyigit I, Bulut I, Coskun E,	Turkey	2006-

© 2017 Karimkhani C et al. *JAMA Dermatology.*

	Dabak S, Deveci F, Fidan F, Kaynar H, Uzaslan EK, Onbasi K, Ozkurt S, Karakis GP, Sahan S, Sahin U, Oguzulgen K, Yildiz F, Mungan D, Yorgancioglu A, Gemicioglu B, Fuat Kalyoncu A. Prevalence and Risk Factors of Allergies in Turkey (PARFAIT): results of a multicentre cross-sectional study in adults. Eur Respir J. 2009; 33(4): 724-33.		2008
	Talay F, Kurt B, Tug T, Yilmaz F, Goksugur N. Prevalence and risk factors of asthma and allergic diseases among schoolchildren in Bolu, Turkey. Acta Paediatr. 2008; 97(4): 459-62.	Turkey	2005-2007
	Tamer E, Ilhan MN, Polat M, Lenk N, Alli N. Prevalence of skin diseases among pediatric patients in Turkey. J Dermatol. 2008; 35(7): 413-8.	Turkey	2004-2006
	Yuksel H, Dinc G, Sakar A, Yilmaz O, Yorgancioglu A, Celik P, Ozcan C. Prevalence and comorbidity of allergic eczema, rhinitis, and asthma in a city in western Turkey. J Investig Allergol Clin Immunol. 2008; 18(1): 31-5.	Turkey	2005-2007
	Odhiambo JA, Williams HC, Clayton TO, Robertson CF, Asher MI, ISAAC Phase Three Study Group. Global variations in prevalence of eczema symptoms in children from ISAAC Phase Three. J Allergy Clin Immunol. 2009; 124(6): 1251-1258.	Ukraine	1998, 2002
	Williams H, Stewart A, Von Mutius E, Cookson W, Anderson HR. Is eczema really on the increase worldwide. J Allergy Clin Immunol. 2008; 121(4): 947-954.	Ukraine	1998, 2002
	Punekar YS, Sheikh A. Establishing the incidence and prevalence of clinician-diagnosed allergic conditions in children and adolescents using routinely collected data from general practices. Clin Exp Allergy. 2009; 39(8): 1209-16.	United Kingdom	2008
	Rea JN, Newhouse ML, Halil T. Skin disease in Lambeth. A community study of prevalence and use of medical care. Br J Prev Soc Med. 1976; 30(2): 107-14.	United Kingdom	1976
	Schofield JK, Fleming D, Grindlay D, Williams H. Skin conditions are the commonest new reason people present to general practitioners in England and Wales. Br J Dermatol. 2011; 165(5): 1044-50.	United Kingdom	2006
	Simpson CR, Newton J, Hippisley-Cox J, Sheikh A. Trends in the epidemiology and prescribing of medication for eczema in England. J R Soc Med. 2009; 102(3): 108-17.	United Kingdom	2001-2004

© 2017 Karimkhani C et al. *JAMA Dermatology.*

	Steele K. Primary dermatological care in general practice. J R Coll Gen Pract. 1984; 34(258): 22-3.	United Kingdom	1983
	Williams H, Stewart A, Von Mutius E, Cookson W, Anderson HR. Is eczema really on the increase worldwide. J Allergy Clin Immunol. 2008; 121(4): 947-954.	United Kingdom	1992-1995, 2001-2002
	Agency for Healthcare Research and Quality. United States Medical Expenditure Panel Survey 1996-2011. Agency for Healthcare Research and Quality.	United States	1996-2011
	Agency for Healthcare Research and Quality. United States Medical Expenditure Panel Survey 2002-2009.	United States	2002-2009
	Laughter D, Istvan JA, Tofte SJ, Hanifin JM. The prevalence of atopic dermatitis in Oregon schoolchildren. J Am Acad Dermatol. 2000; 43(4): 649-55.	United States	1999
	National Center for Health Statistics (NCHS), Centers for Disease Control and Prevention (CDC). United States Skin Conditions and Related Need for Medical Care Among Persons 1-74 Years 1971-1974. Hyattsville, United States: US Department of Health, Education, and Welfare, 1978.	United States	1971-1974
	Odhiambo JA, Williams HC, Clayton TO, Robertson CF, Asher MI, ISAAC Phase Three Study Group. Global variations in prevalence of eczema symptoms in children from ISAAC Phase Three. J Allergy Clin Immunol. 2009; 124(6): 1251-1258.	Untied States	2003
	Shaw TE, Currie GP, Koudelka CW, Simpson EL. Eczema prevalence in the United States: data from the 2003 National Survey of Children's Health. J Invest Dermatol. 2011; 131(1): 67-73.	United States	2003
	Silverberg JI, Hanifin JM. Adult eczema prevalence and associations with asthma and other health and demographic factors: a US population-based study. J Allergy Clin Immunol. 2013; 132(5): 1132-8.	United States	2010
	Silverberg JI, Silverberg NB. Childhood atopic dermatitis and warts are associated with increased risk of infection: A US population-based study. J Allergy Clin Immunol. 2014; 133(4): 1041-7.	United States	2007
	Sun Y, Sundell J. Life style and home environment are associated with racial disparities of asthma and allergy in Northeast Texas children. Sci Total Environ. 2011; 409(20): 4229-34.	United States	2008-2009
	Williams H, Stewart A, Von Mutius E, Cookson W,	United	1995,

	Anderson HR. Is eczema really on the increase worldwide. J Allergy Clin Immunol. 2008; 121(4): 947-954.	States	2003
	Odhiambo JA, Williams HC, Clayton TO, Robertson CF, Asher MI, ISAAC Phase Three Study Group. Global variations in prevalence of eczema symptoms in children from ISAAC Phase Three. J Allergy Clin Immunol. 2009; 124(6): 1251-1258.	Uruguay	2002
	Solé D, Mallol J, Wandalsen GF, Aguirre V, Latin American ISAAC Phase 3 Study Group. Prevalence of symptoms of eczema in Latin America: results of the International Study of Asthma and Allergies in Childhood (ISAAC) Phase 3. J Investig Allergol Clin Immunol. 2010; 20(4): 311-23.	Uruguay	2001-2003
	Williams H, Stewart A, Von Mutius E, Cookson W, Anderson HR. Is eczema really on the increase worldwide. J Allergy Clin Immunol. 2008; 121(4): 947-954.	Uruguay	1994, 2002
	Odhiambo JA, Williams HC, Clayton TO, Robertson CF, Asher MI, ISAAC Phase Three Study Group. Global variations in prevalence of eczema symptoms in children from ISAAC Phase Three. J Allergy Clin Immunol. 2009; 124(6): 1251-1258.	Venezuela	2002
	Solé D, Mallol J, Wandalsen GF, Aguirre V, Latin American ISAAC Phase 3 Study Group. Prevalence of symptoms of eczema in Latin America: results of the International Study of Asthma and Allergies in Childhood (ISAAC) Phase 3. J Investig Allergol Clin Immunol. 2010; 20(4): 311-23.	Venezuela	2001-2003
	Odhiambo JA, Williams HC, Clayton TO, Robertson CF, Asher MI, ISAAC Phase Three Study Group. Global variations in prevalence of eczema symptoms in children from ISAAC Phase Three. J Allergy Clin Immunol. 2009; 124(6): 1251-1258.	Vietnam	2001
	Odhiambo JA, Williams HC, Clayton TO, Robertson CF, Asher MI, ISAAC Phase Three Study Group. Global variations in prevalence of eczema symptoms in children from ISAAC Phase Three. J Allergy Clin Immunol. 2009; 124(6): 1251-1258.	Wales	2002
	Williams H, Stewart A, Von Mutius E, Cookson W, Anderson HR. Is eczema really on the increase worldwide. J Allergy Clin Immunol. 2008; 121(4):	Wales	1995, 2002

© 2017 Karimkhani C et al. *JAMA Dermatology.*

	947-954.		
	Mallen CD, Mottram S, Wynne-Jones G, Thomas E. Birth-related exposures and asthma and allergy in adulthood: a population-based cross-sectional study of young adults in North Staffordshire. J Asthma. 2008; 45(4): 309-12.	West Midlands	2002
	Li F, Zhou Y, Li S, Jiang F, Jin X, Yan C, Tian Y, Zhang Y, Tong S, Shen X. Prevalence and risk factors of childhood allergic diseases in eight metropolitan cities in China: a multicenter study. BMC Public Health. 2011; 437.	Xinjiang	2005
	Odhiambo JA, Williams HC, Clayton TO, Robertson CF, Asher MI, ISAAC Phase Three Study Group. Global variations in prevalence of eczema symptoms in children from ISAAC Phase Three. J Allergy Clin Immunol. 2009; 124(6): 1251-1258.	Xinjian	2001
	Odhiambo JA, Williams HC, Clayton TO, Robertson CF, Asher MI, ISAAC Phase Three Study Group. Global variations in prevalence of eczema symptoms in children from ISAAC Phase Three. J Allergy Clin Immunol. 2009; 124(6): 1251-1258.	Yucatan	2002-2003
	Paek SY, Koriakos A, Saxton-Daniels S, Pandya AG. Skin diseases in rural Yucatan, Mexico. Int J Dermatol. 2012; 51(7): 823-8.	Yucatan	2009-2010
	Solé D, Mallol J, Wandalsen GF, Aguirre V, Latin American ISAAC Phase 3 Study Group. Prevalence of symptoms of eczema in Latin America: results of the International Study of Asthma and Allergies in Childhood (ISAAC) Phase 3. J Investig Allergol Clin Immunol. 2010; 20(4): 311-23.	Yucatan	2001-2003
Fungal skin diseases	Kilkenny M, Stathakis V, Jolley D, Marks R. Maryborough skin health survey: prevalence and sources of advice for skin conditions. Australas J Dermatol. 1998; 39(4): 233-7.	Australia	1996
	Plunkett A, Merlin K, Gill D, Zuo Y, Jolley D, Marks R. The frequency of common nonmalignant skin conditions in adults in central Victoria, Australia. Int J Dermatol. 1999; 38(12): 901-8.	Australia	1997-1998
	Bissek A-CZ-K, Tabah EN, Kouotou E, Sini V, Yepnjio FN, Nditanchou R, Nchufor RN, Defo D, Dema F, Fonsah JY, Njamnshi AK, Muna WFT. The spectrum of skin diseases in a rural setting in Cameroon (sub-Saharan Africa). BMC Dermatol. 2012; 7.	Cameroon	2010

© 2017 Karimkhani C et al. *JAMA Dermatology.*

	Fulgence KK, Abibatou K, Vincent D, Henriette V, Etienne AK, Kiki-Barro PC, Yavo W, Koné M, Hervé Menan EI. Tinea capitis in schoolchildren in southern Ivory Coast. Int J Dermatol. 2013; 52(4): 456-60.	Cote d'Ivoire	2008-2009
	Abdel-Hafez K, Abdel-Aty MA, Hofny ERM. Prevalence of skin diseases in rural areas of Assiut Governorate, Upper Egypt. Int J Dermatol. 2003; 42(11): 887-92.	Egypt	1994-1996
	Yamamah GA, Emam HM, Abdelhamid MF, Elsaie ML, Shehata H, Farid T, Kamel MI, Taalat AA. Epidemiologic study of dermatologic disorders among children in South Sinai, Egypt. Int J Dermatol. 2012; 51(10): 1180-5.	Egypt	2008-2009
	Ali J, Yifru S, Woldeamanuel Y. Prevalence of tinea capitis and the causative agent among school children in Gondar, North West Ethiopia. Ethiop Med J. 2011; 47(4): 261-9.	Ethiopia	2007-2008
	Figueroa JI, Fuller LC, Abraha A, Hay RJ. Dermatology in southwestern Ethiopia: rationale for a community approach. Int J Dermatol. 1998; 37(10): 752-8.	Ethiopia	1994-1995
	Murgia V, Bilcha KD, Shibeshi D. Community dermatology in Debre Markos: an attempt to define children's dermatological needs in a rural area of Ethiopia. Int J Dermatol. 2010; 49(6): 666-71.	Ethiopia	2009
	Hogewoning A, Amoah A, Bavinck JNB, Boakye D, Yazdanbakhsh M, Adegnika A, De Smedt S, Fonteyne Y, Willemze R, Lavrijsen A. Skin diseases among schoolchildren in Ghana, Gabon, and Rwanda. Int J Dermatol. 2013; 52(5): 589-600.	Gabon	2005
	Hogewoning AA, Adegnika AA, Bouwes Bavinck JN, Yazdanbakhsh M, Kremsner PG, van der Raaij-Helmer EMH, Staats CCG, Willemze R, Lavrijsen APM. Prevalence and causative fungal species of tinea capitis among schoolchildren in Gabon. Mycoses. 2011; 54(5): e354-359.	Gabon	2005
	Augustin M, Herberger K, Hintzen S, Heigel H, Franzke N, Schäfer I. Prevalence of skin lesions and need for treatment in a cohort of 90,880 workers. Br J Dermatol. 2011; 165(4): 865-73.	Germany	2004-2009
	Hogewoning A, Amoah A, Bavinck JNB, Boakye D, Yazdanbakhsh M, Adegnika A, De Smedt S, Fonteyne Y, Willemze R, Lavrijsen A. Skin diseases among schoolchildren in Ghana, Gabon, and Rwanda. Int J Dermatol. 2013; 52(5): 589-600.	Gabon	2004, 2007

© 2017 Karimkhani C et al. *JAMA Dermatology.*

	Fung WK, Lo KK. Prevalence of skin disease among school children and adolescents in a Student Health Service Center in Hong Kong. Pediatr Dermatol. 2000; 17(6): 440-6.	Hong Kong Special Administrati ve Region of China	1996-1997
	Grills N, Grills C, Spelman T, Stoove M, Hellard M, El-Hayek C, Singh R. Prevalence survey of dermatological conditions in mountainous north India. Int J Dermatol. 2012; 51(5): 579-87.	India	2010
	Grover S, Ranyal RK, Bedi MK. A cross section of skin diseases in rural Allahabad. Indian J Dermatol. 2008; 53(4): 179-81.	India	2005
	Patel JK, Vyas AP, Berman B, Vierra M. Incidence of childhood dermatosis in India. Skinmed. 2010; 8(3): 136-42.	India	2000-2002
	Saw SM, Koh D, Adjani MR, Wong ML, Hong CY, Lee J, Chia SE, Munoz CP, Ong CN. A population-based prevalence survey of skin diseases in adolescents and adults in rural Sumatra, Indonesia, 1999. Trans R Soc Trop Med Hyg. 2001; 95(4): 384-8.	Indonesia	1999
	Al-Rubiay KK, Al-Rubaiy LK. Dermatoepidemiology: A Household Survey Among Two Urban Areas In Basrah City, Iraq. The Internet Journal of Dermatology. 2006; 4(2): 10.	Iraq	2005
	Chepchirchir A, Bii C, Ndinya-Achola JO. Dermatophyte infections in primary school children in Kibera slums of Nairobi. East Afr Med J. 2009; 86(2): 59-68.	Kenya	2006-2007
	Schmeller W, Baumgartner S, Dzikus A. Dermatophytomycoses in children in rural Kenya: the impact of primary health care. Mycoses. 1997; 40(1-2): 55-63.	Kenya	1993, 1995
	Schmeller W, Dzikus A. Skin diseases in children in rural Kenya: long-term results of a dermatology project within the primary health care system. Br J Dermatol. 2001; 144(1): 118-24.	Kenya	1999
	Carod J-F, Ratsitorahina M, Raherimandimby H, Hincky Vitrat V, Ravaolimalala Andrianaja V, Contet-Audonneau N. Outbreak of Tinea capitis and corporis in a primary school in Antananarivo, Madagascar. J Infect Dev Ctries. 2011; 5(10): 732-6.	Madagascar	2005
	Contet-Audonneau N, Grosjean P, Razanakolona L-R, Andriantsinjovina T, Rapelanoro R. [Tinea capitis in Madagascar: a survey in a primary school	Madagascar	2002

© 2017 Karimkhani C et al. *JAMA Dermatology.*

	in Antsirabe]. Ann Dermatol Venereol. 2006; 133(1): 22-5.		
	Sidat MM, Correia D, Buene TP. Tinea capitis among children at one suburban primary school in the City of Maputo, Mozambique. Rev Soc Bras Med Trop. 2007; 40(4): 473-5.	Mozambiqu e	2001
	Walker SL, Shah M, Hubbard VG, Pradhan HM, Ghimire M. Skin disease is common in rural Nepal: results of a point prevalence study. Br J Dermatol. 2008; 158(2): 334-8.	Nepal	2004-2006
	Ogunbiyi AO, Owoaje E, Ndahi A. Prevalence of skin disorders in school children in Ibadan, Nigeria. Pediatr Dermatol. 2005; 22(1): 6-10.	Niger	2002-2004
	Adefemi SA, Odeigah LO, Alabi KM. Prevalence of dermatophytosis among primary school children in Oke-Oyi community of Kwara state. Niger J Clin Pract. 2011; 14(1): 23-8.	Nigeria	2005
	Ayanbimpe GM, Taghir H, Diya A, Wapwera S. Tinea capitis among primary school children in some parts of central Nigeria. Mycoses. 2008; 51(4): 336-40.	Nigeria	2004
	Eja ME, Arikpo GE, Enyi-Idoh KH, Etim SE, Etta HE. Efficacy of local herbal therapy in the management of dermatophytosis among primary school children in Cross River State, South-south Nigeria. Afr J Med Med Sci. 2009; 38(2): 135-41.	Nigeria	2006
	Emele FE, Oyeka CA. Tinea capitis among primary school children in Anambra state of Nigeria. Mycoses. 2008; 51(6): 536-41.	Nigeria	2002-2005
	Ngwogu AC, Otokunefor TV. Epidemiology of dermatophytoses in a rural community in Eastern Nigeria and review of literature from Africa. Mycopathologia. 2007; 164(4): 149-58.	Nigeria	2003-2004
	Ogunbiyi AO, Omigbodun Y, Owoaje E. Prevalence of skin disorders in school children in southwest Nigeria. Int J Adolesc Med Health. 2009; 21(2): 235-41.	Nigeria	2006-2008
	Oyedeji O, Okeniyi J, Ogunlesi T, Onayemi O, Oyedeji G, Oyelami O. Parental factors influencing the prevalence of skin infections and infestations among Nigerian primary school pupils. The Internet Journal of Dermatology. 2006; 3(2): 6.	Nigeria	2003
	Popoola TOS, Ojo DA, Alabi RO. Prevalence of dermatophytosis in junior secondary schoolchildren in Ogun State, Nigeria. Mycoses. 2006; 49(6): 499-503.	Nigeria	2001-2003

© 2017 Karimkhani C et al. *JAMA Dermatology.*

	Flores JM, Castillo VB, Franco FC, Huata AB. Superficial fungal infections: clinical and epidemiological study in adolescents from marginal districts of Lima and Callao, Peru. J Infect Dev Ctries. 2009; 3(4): 313-7.	Peru	2006
	Hogewoning A, Amoah A, Bavinck JNB, Boakye D, Yazdanbakhsh M, Adegnika A, De Smedt S, Fonteyne Y, Willemze R, Lavrijsen A. Skin diseases among schoolchildren in Ghana, Gabon, and Rwanda. Int J Dermatol. 2013; 52(5): 589-600.	Rwanda	2007
	Amin TT, Ali A, Kaliyadan F. Skin disorders among male primary school children in Al Hassa, Saudi Arabia: prevalence and socio-demographic correlates--a comparison of urban and rural populations. Rural Remote Health. 2011; 11(1): 1517.	Saudi Arabia	2009
	Pérez-González M, Torres-Rodríguez JM, Martínez-Roig A, Segura S, Griera G, Triviño L, Pasarín M. Prevalence of tinea pedis, tinea unguium of toenails and tinea capitis in school children from Barcelona. Rev Iberoam Micol. 2009; 26(4): 228-32.	Spain	2003-2004
	Perera A, Atukorale DN, Sivayogan S, Ariyaratne VS, Karunaratne LDA. Prevalence of skin diseases in suburban Sri Lanka. Ceylon Med J. 2000; 45(3): 123-8.	Sri Lanka	1997
	Chen G-Y, Cheng Y-W, Wang C-Y, Hsu T-J, Hsu MM-L, Yang P-T, Chen W-C. Prevalence of skin diseases among schoolchildren in Magong, Penghu, Taiwan: a community-based clinical survey. J Formos Med Assoc. 2008; 107(1): 21-9.	Taiwan	2005
	Ferié J, Dinkela A, Mbata M, Idindili B, Schmid-Grendelmeier P, Hatz C. Skin disorders among school children in rural Tanzania a nd an assessment of therapeutic needs. Trop Doct. 2006; 36(4): 219-21.	Tanzania	2003
	Komba EV, Mgonda YM. The spectrum of dermatological disorders among primary school children in Dar es Salaam. BMC Public Health. 2010; 10(1): 765.	Tanzania	2007-2010
	Kiraz N, Metintas S, Oz Y, Koc F, Koku Aksu EA, Kalyoncu C, Kasifoglu N, Cetin E, Arikan I. The prevalence of tinea pedis and tinea manuum in adults in rural areas in Turkey. Int J Environ Health Res. 2010; 20(5): 379-86.	Turkey	2007-2009
	Cantrell WC, Jacobs MK, Sobera JO, Parrish CA,	United	2008-

© 2017 Karimkhani C et al. *JAMA Dermatology.*

	Warner J, Elewski BE. Tinea capitis in Birmingham: survey of elementary school students. Pediatr Dermatol. 2011; 28(4): 476-7.	States	2010
	Paek SY, Koriakos A, Saxton-Daniels S, Pandya AG. Skin diseases in rural Yucatan, Mexico. Int J Dermatol. 2012; 51(7): 823-8.	Yucatan	2009-2010
Pruritus	Canadian Institute for Health Information (CIHI). Canada Discharge Abstract Database. Ottawa, Canada: Canadian Institute for Health Information (CIHI).	Canada	1994-2001
	Canadian Institute for Health Information (CIHI). Canada National Ambulatory Care Reporting System. Ottawa, Canada: Canadian Institute for Health Information (CIHI).	Canada	2002-2009
	Misery L, Rahhali N, Duhamel A, Taieb C. Epidemiology of pruritus in france. Acta Derm Venereol. 2012; 92(5): 421-2.	France	2010
	Frese T, Herrmann K, Sandholzer H. Pruritus as Reason for Encounter in General Practice. J Clin Med Res. 2011; 3(5): 223-9.	Germany	1999-2000
	Matterne U, Apfelbacher CJ, Loerbroks A, Schwarzer T, Büttner M, Ofenloch R, Diepgen TL, Weisshaar E. Prevalence, correlates and characteristics of chronic pruritus: a population-based cross-sectional study. Acta Derm Venereol. 2011; 91(6): 674-9.	Germany	2008-2009
	Ständer S, Schäfer I, Phan NQ, Blome C, Herberger K, Heigel H, Augustin M. Prevalence of chronic pruritus in Germany: results of a cross-sectional study in a sample working population of 11,730. Dermatology (Basel). 2010; 221(3): 229-35.	Germany	2008
	Al-Rubiay KK, Al-Rubaiy LK. Dermatoepidemiology: A Household Survey Among Two Urban Areas In Basrah City, Iraq. Internet J Dermatol. 2006; 4(2): 10.	Iraq	2005
	Becerril Angeles M, Vázquez Merino CL, Angeles Garay U, Alvarado Moctezuma LE, Vilchis Guízar E. Prevalence of allergic diseases in the elderly. Rev Alerg Mex. 2008; 55(3): 85-91.	Mexico	2006-2008
	Mohammedamin RS, van der Wouden JC, Koning S, van der Linden MW, Schellevis FG, van Suijlekom-Smit LW, Koes BW. Increasing incidence of skin disorders in children? A comparison between 1987 and 2001. BMC Dermatol. 2006; 6(4).	Netherlands	1987, 2001

© 2017 Karimkhani C et al. *JAMA Dermatology.*

	Ogunbiyi AO, Omigbodun Y, Owoaje E. Prevalence of skin disorders in school children in southwest Nigeria. Int J Adolesc Med Health. 2009; 21(2): 235-41.	Nigeria	2006-2008
	Dalgard F, Svensson A, Holm JØ, Sundby J. Self-reported skin morbidity in Oslo. Associations with sociodemographic factors among adults in a cross-sectional study. BMC Dermatol. 2004; 6(4): 452-7.	Norway	2000-2001
	Norwegian Directorate of Health. Norway Patient Register.	Norway	2008-2012
	Gutierrez E, Galarza C, Ramos W, Tello M, Rojas I, Chia H, Ronceros G, Ortega-Loayza A. Prevalence of skin diseases in a rural area of Peruvian Amazonia. Dermatol Peru. 2009; 19(2): 104-12.	Peru	2005
	Popescu R, Popescu CM, Williams HC, Forsea D. The prevalence of skin conditions in Romanian school children. Br J Dermatol. 1999; 140(5): 891-6.	Romania	1995
	Perera A, Atukorale DN, Sivayogan S, Ariyaratne VS, Karunaratne LDA. Prevalence of skin diseases in suburban Sri Lanka. Ceylon Med J. 2000; 45(3): 123-8.	Sri Lanka	1997
	Ferié J, Dinkela A, Mbata M, Idindili B, Schmid-Grendelmeier P, Hatz C. Skin disorders among school children in rural Tanzania a nd an assessment of therapeutic needs. Trop Doct. 2006; 36(4): 219-21.	Tanzania	2003
	Kiliç A, Gül U, Aslan E, Soylu S. Dermatological findings in the senior population of nursing homes in Turkey. Arch Gerontol Geriatr. 2008; 47(1): 93-8.	Turkey	2006
	Agency for Healthcare Research and Quality. United States Medical Expenditure Panel Survey 1996-2011. Agency for Healthcare Research and Quality.	United States	1996-2011
	Analytical Sciences, Inc., National Center for Health Statistics (NCHS), Centers for Disease Control and Prevention (CDC), US Census Bureau. United States National Ambulatory Medical Care Survey. Hyattsville, United States: National Center for Health Statistics (NCHS), Centers for Disease Control and Prevention (CDC).	United States	1995-2001
	Analytical Sciences, Inc., National Center for Health Statistics (NCHS), Centers for Disease Control and Prevention (CDC), US Census Bureau.	United States	1995-2001

© 2017 Karimkhani C et al. *JAMA Dermatology.*

	United States National Hospital Ambulatory Medical Care Survey. Hyattsville, United States: National Center for Health Statistics (NCHS), Centers for Disease Control and Prevention (CDC).		
	Constella Group, National Center for Health Statistics (NCHS), Centers for Disease Control and Prevention (CDC), US Census Bureau. United States National Ambulatory Medical Care Survey. Hyattsville, United States: National Center for Health Statistics (NCHS), Centers for Disease Control and Prevention (CDC).	United States	2002-2010
	National Center for Health Statistics (NCHS), Centers for Disease Control and Prevention (CDC), SRA International, Inc., US Census Bureau. United States National Hospital Ambulatory Medical Care Survey 2007. Hyattsville, United States: National Center for Health Statistics (NCHS), Centers for Disease Control and Prevention (CDC).	United States	2007-2010
	National Center for Health Statistics (NCHS), Centers for Disease Control and Prevention (CDC), US Census Bureau. United States National Ambulatory Medical Care Survey 1995 and United States National Hospital Ambulatory Medical Care Survey.	United States	1995-2009
Psoriasis	Soriano ER, Rosa J, Velozo E, Schpilberg M, Imamura PM, Diaz J, Catoggio LJ. Incidence and prevalence of psoriatic arthritis in Buenos Aires, Argentina: a 6-year health management organization-based study. Rheumatology. 2011; 50(4): 729-34.	Argentina	2000-2006
	Australian Bureau of Statistics. Australia National Health Survey. Canberra, Australia: Australian Bureau of Statistics.	Australia	1995-1996, 2001
	Australian Bureau of Statistics. Australia National Health Survey 2004-2005.	Australia	2004-2005
	Australian Bureau of Statistics. Australia National Health Survey 2007-2008. Canberra, Australia: Australian Bureau of Statistics.	Australia	2007-2008
	Li R, Sun J, Ren L-M, Wang H-Y, Liu W-H, Zhang X-W, Chen S, Mu R, He J, Zhao Y, Long L, Liu Y-Y, Liu X, Lu X-L, Li Y-H, Wang S-Y, Pan S-S, Li C, Wang H-Y, Li Z-G. Epidemiology of eight common rheumatic diseases in China: a large-scale cross-sectional survey in Beijing. Rheumatology. 2012; 51(4): 721-9.	Beijing	2009-2011
	Carneiro JN, Paula AP de, Martins GA. Psoriatic	Brazil	2009-

	arthritis in patients with psoriasis: evaluation of clinical and epidemiological features in 133 patients followed at the University Hospital of Brasília. An Bras Dermatol. 2012; 87(4): 539-44.		2010
	Estrada Castañón R, Torres Bibiano B, Alarcón Hernández H, Villegas Arrizón A, Martínez Sandoval E, Chávez López G, Andersson N. Epidemiología cutánea en dos sectores de atención médica en Guerrero, México; Cutaneous epidemiology in two sectors of Guerrero, Mexico. Dermatol rev mex. 1992; 36(1): 29-34.	Central Latin America	1989-1991
	Ding X, Wang T, Shen Y, Wang X, Zhou C, Tian S, Liu Y, Peng G, Zhou J, Xue S, Wang R, Tang Y, Meng X, Pei G, Bai Y, Liu Q, Li H, Zhang J. Prevalence of psoriasis in China: a population-based study in six cities. Eur J Dermatol. 2012; 22(5): 663-7.	China	2009-2011
	Li R, Sun J, Ren L-M, Wang H-Y, Liu W-H, Zhang X-W, Chen S, Mu R, He J, Zhao Y, Long L, Liu Y-Y, Liu X, Lu X-L, Li Y-H, Wang S-Y, Pan S-S, Li C, Wang H-Y, Li Z-G. Epidemiology of eight common rheumatic diseases in China: a large-scale cross-sectional survey in Beijing. Rheumatology. 2012; 51(4): 721-9.	Chongqing	2009-2011
	Fung WK, Lo KK. Prevalence of skin disease among school children and adolescents in a Student Health Service Center in Hong Kong. Pediatr Dermatol. 2000; 17(6): 440-6.	East Asia	1996-1997
	Gibbs, S. Skin disease and socioeconomic conditions in rural Africa: Tanzania. Int J Dermatol. 1996; 35(9): 633-9.	Eastern Sub-Saharan Africa	1994
	Abdel-Hafez K, Abdel-Aty MA, Hofny ERM. Prevalence of skin diseases in rural areas of Assiut Governorate, Upper Egypt. Int J Dermatol. 2003; 42(11): 887-92.	Egypt	2001
	Yamamah GA, Emam HM, Abdelhamid MF, Elsaie ML, Shehata H, Farid T, Kamel MI, Taalat AA. Epidemiologic study of dermatologic disorders among children in South Sinai, Egypt. Int J Dermatol. 2012; 51(10): 1180-5.	Egypt	2008-2009
	Figueroa JI, Fuller LC, Abraha A, Hay RJ. Dermatology in southwestern Ethiopia: rationale for a community approach. Int J Dermatol. 1998; 37(10): 752-8.	Ethiopia	1994
	Leekassa R, Bizuneh E, Alem A, Fekadu A, Shibre T. Community diagnosis of common skin diseases	Ethiopia	1998

© 2017 Karimkhani C et al. *JAMA Dermatology.*

	in the Zay community of the Zeway Islands, Ethiopia. Ethiop Med J. 2005; 43(3): 189-95.		
	Wolkenstein P, Revuz J, Roujeau JC, Bonnelye G, Grob JJ, Bastuji-Garin S, French Society of Dermatology. Psoriasis in France and associated risk factors: results of a case-control study based on a large community survey. Dermatology (Basel). 2009; 218(2): 103-9.	France	2005
	Hogewoning A, Amoah A, Bavinck JNB, Boakye D, Yazdanbakhsh M, Adegnika A, De Smedt S, Fonteyne Y, Willemze R, Lavrijsen A. Skin diseases among schoolchildren in Ghana, Gabon, and Rwanda. Int J Dermatol. 2013; 52(5): 589-600.	Gabon	2005
	Augustin M, Glaeske G, Radtke MA, Christophers E, Reich K, Schäfer I. Epidemiology and comorbidity of psoriasis in children. Br J Dermatol. 2010; 162(3): 633-6.	Germany	2005
	Augustin M, Herberger K, Hintzen S, Heigel H, Franzke N, Schäfer I. Prevalence of skin lesions and need for treatment in a cohort of 90,880 workers. Br J Dermatol. 2011; 165(4): 865-73.	Germany	2002-2008
	Frese T, Herrmann K, Sandholzer H. Pruritus as Reason for Encounter in General Practice. J Clin Med Res. 2011; 3(5): 223-9.	Germany	1999-2000
	Radtke MA, Reich K, Blome C, Rustenbach S, Augustin M. Prevalence and clinical features of psoriatic arthritis and joint complaints in 2009 patients with psoriasis: results of a German national survey. J Eur Acad Dermatol Venereol. 2009; 23(6): 683-91.	Germany	2009
	Reich K, Krüger K, Mössner R, Augustin M. Epidemiology and clinical pattern of psoriatic arthritis in Germany: a prospective interdisciplinary epidemiological study of 1511 patients with plaque-type psoriasis. Br J Dermatol. 2009; 160(5): 1040-7.	Germany	2004-2005
	Schaefer I, Rustenbach SJ, Zimmer L, Augustin M. Prevalence of skin diseases in a cohort of 48,665 employees in Germany. Dermatology (Basel). 2008; 217(2): 169-72.	Germany	2001-2005
	Troitzsch P, Paulista Markus MR, Dörr M, Felix SB, Jünger M, Schminke U, Schmidt C-O, Völzke H, Baumeister SE, Arnold A. Psoriasis is associated with increased intima-media thickness--the Study of Health in Pomerania (SHIP). Atherosclerosis. 2012; 225(2): 486-90.	Germany	2002-2006
	Hogewoning A, Amoah A, Bavinck JNB, Boakye	Ghana	2004,

© 2017 Karimkhani C et al. *JAMA Dermatology.*

	D, Yazdanbakhsh M, Adegnika A, De Smedt S, Fonteyne Y, Willemze R, Lavrijsen A. Skin diseases among schoolchildren in Ghana, Gabon, and Rwanda. Int J Dermatol. 2013; 52(5): 589-600.		2007
	Ding X, Wang T, Shen Y, Wang X, Zhou C, Tian S, Liu Y, Peng G, Zhou J, Xue S, Wang R, Tang Y, Meng X, Pei G, Bai Y, Liu Q, Li H, Zhang J. Prevalence of psoriasis in China: a population-based study in six cities. Eur J Dermatol. 2012; 22(5): 663-7.	Hebei	2009-2011
	Ding X, Wang T, Shen Y, Wang X, Zhou C, Tian S, Liu Y, Peng G, Zhou J, Xue S, Wang R, Tang Y, Meng X, Pei G, Bai Y, Liu Q, Li H, Zhang J. Prevalence of psoriasis in China: a population-based study in six cities. Eur J Dermatol. 2012; 22(5): 663-7.	Henan	2009-2011
	Love TJ, Gudjonsson JE, Valdimarsson H, Gudbjornsson B. Psoriatic arthritis and onycholysis -- results from the cross-sectional Reykjavik psoriatic arthritis study. J. Rheumatol. 2012; 39(7): 1441-4.	Iceland	2009-2011
	Grills N, Grills C, Spelman T, Stoove M, Hellard M, El-Hayek C, Singh R. Prevalence survey of dermatological conditions in mountainous north India. Int J Dermatol. 2012; 51(5): 579-87.	India	2010
	Kuruvila M, Dubey S, Gahalaut P. Pattern of skin diseases among migrant construction workers in Mangalore. Indian J Dermatol Venereol Leprol. 2006; 72(2): 129-32.	India	2005
	Prasad PVS, Bikku B, Kaviarasan PK, Senthilnathan A. A clinical study of psoriatic arthropathy. Indian J Dermatol Venereol Leprol. 2007; 73(3): 166-70.	India, rural	2005
	Lee J, Koh D, Andijani M, Saw SM, Munoz C, Chia SE, Wong ML, Hong CY, Ong CN. Effluents from a pulp and paper mill: a skin and health survey of children living in upstream and downstream villages. Occup Environ Med. 2002; 59(6): 373-9.	Indonesia	1999
	Saw SM, Koh D, Adjani MR, Wong ML, Hong CY, Lee J, Chia SE, Munoz CP, Ong CN. A population-based prevalence survey of skin diseases in adolescents and adults in rural Sumatra, Indonesia, 1999. Trans R Soc Trop Med Hyg. 2001; 95(4): 384-8.	Indonesia	1999
	Ding X, Wang T, Shen Y, Wang X, Zhou C, Tian S, Liu Y, Peng G, Zhou J, Xue S, Wang R, Tang Y,	Inner Mongolia	2009-2011

© 2017 Karimkhani C et al. *JAMA Dermatology.*

	Meng X, Pei G, Bai Y, Liu Q, Li H, Zhang J. Prevalence of psoriasis in China: a population-based study in six cities. Eur J Dermatol. 2012; 22(5): 663-7.		
	Jamshidi F, Bouzari N, Seirafi H, Farnaghi F, Firooz A. The prevalence of psoriatic arthritis in psoriatic patients in Tehran, Iran. Arch Iran Med. 2008; 11(2): 162-5.	Iran	2003-2004
	Li R, Sun J, Ren L-M, Wang H-Y, Liu W-H, Zhang X-W, Chen S, Mu R, He J, Zhao Y, Long L, Liu Y-Y, Liu X, Lu X-L, Li Y-H, Wang S-Y, Pan S-S, Li C, Wang H-Y, Li Z-G. Epidemiology of eight common rheumatic diseases in China: a large-scale cross-sectional survey in Beijing. Rheumatology. 2012; 51(4): 721-9.	Iran	2003-2004
	Al-Rubiay KK, Al-Rubaiy LK. Dermatoepidemiology: A Household Survey Among Two Urban Areas In Basrah City, Iraq. The Internet Journal of Dermatology. 2006; 4(2): 10.	Iraq	2005
	Haroon M, Kirby B, FitzGerald O. High prevalence of psoriatic arthritis in patients with severe psoriasis with suboptimal performance of screening questionnaires. Ann Rheum Dis. 2013; 72(5): 736-40.	Iceland	2010-2012
	De Angelis R, Salaffi F, Grassi W. Prevalence of spondyloarthropathies in an Italian population sample: a regional community-based study. Scand J Rheumatol. 2007; 36(1): 14-21.	Italy	2006
	Naldi L, Parazzini F, Gallus S, GISED Study Centres. Prevalence of atopic dermatitis in Italian schoolchildren: factors affecting its variation. Acta Derm. Venereol. 2009; 89(2): 122-5.	Italy	1997
	Saraceno R, Mannheimer R, Chimenti S. Regional distribution of psoriasis in Italy. J Eur Acad Dermatol Venereol. 2008; 22(3): 324-9.	Italy	2006
	Mahé A, Prual A, Konaté M, Bobin P. Skin diseases of children in Mali: a public health problem. Trans R Soc Trop Med Hyg. 1995; 89(5): 467-70.	Mali	1993-1994
	Walker SL, Shah M, Hubbard VG, Pradhan HM, Ghimire M. Skin disease is common in rural Nepal: results of a point prevalence study. Br J Dermatol. 2008; 158(2): 334-8.	Nepal	2006
	Onayemi O, Isezuo SA, Njoku CH. Prevalence of different skin conditions in an outpatients' setting in north-western Nigeria. Int J Dermatol. 2005; 44(1): 11-Jul.	Nigeria	1999-2001

© 2017 Karimkhani C et al. *JAMA Dermatology.*

	Al-Shammari SA, Al-Sheikh O. Skin morbidity pattern among patients seen at a university primary care clinic in Riyadh, Saudi Arabia. The Gulf Journal of Dermatology and Venereology. 1996; 3: 22-5.	North Africa and Middle East	1993-1994
	Halvorsen JA, Braae Olesen A, Thoresen M, Holm JØ, Bjertness E, Dalgard F. Comparison of self-reported skin complaints with objective skin signs among adolescents. Acta Derm Venereol. 2008; 88(6): 573-7.	Norway	2006
	Olsen AO, Grjibovski A, Magnus P, Tambs K, Harris JR. Psoriasis in Norway as observed in a population-based Norwegian twin panel. Br J Dermatol. 2005; 153(2): 346-51.	Norway	1998
	Gutierrez EL, Galarza C, Ramos W. Prevalencia de Enfermedades Dermatológicas en una comunidad rural de Ucayali, Perú. Dermatol peru. 2009; 19(2): 104-13.	Peru	2005
	Popescu R, Popescu CM, Williams HC, Forsea D. The prevalence of skin conditions in Romanian school children. Br J Dermatol. 1999; 140(5): 891-6.	Romania	1995
	Hogewoning A, Amoah A, Bavinck JNB, Boakye D, Yazdanbakhsh M, Adegnika A, De Smedt S, Fonteyne Y, Willemze R, Lavrijsen A. Skin diseases among schoolchildren in Ghana, Gabon, and Rwanda. Int J Dermatol. 2013; 52(5): 589-600.	Rwanda	2007
	Al-Saeed WY, Al-Dawood KM, Bukhari IA, Bahnassy AA. Prevalence and pattern of skin disorders among female schoolchildren in Eastern Saudi Arabia. Saudi Med J. 2006; 27(2): 227-34.	Saudi Arabia	2003
	Amin TT, Ali A, Kaliyadan F. Skin disorders among male primary school children in Al Hassa, Saudi Arabia: prevalence and socio-demographic correlates--a comparison of urban and rural populations. Rural Remote Health. 2011; 11(1): 1517.	Saudi Arabia	2009
	Ding X, Wang T, Shen Y, Wang X, Zhou C, Tian S, Liu Y, Peng G, Zhou J, Xue S, Wang R, Tang Y, Meng X, Pei G, Bai Y, Liu Q, Li H, Zhang J. Prevalence of psoriasis in China: a population-based study in six cities. Eur J Dermatol. 2012; 22(5): 663-7.	Shandong	2009-2011
	Yang Q, Qu L, Tian H, Hu Y, Peng J, Yu X, Yu C, Pei Z, Wang G, Shi B, Zhang F, Zhang Y, Zhang F. Prevalence and characteristics of psoriatic arthritis	Shandong	2009

© 2017 Karimkhani C et al. *JAMA Dermatology.*

	in Chinese patients with psoriasis. J Eur Acad Dermatol Venereol. 2011; 25(12): 1409-14.		
	Ding X, Wang T, Shen Y, Wang X, Zhou C, Tian S, Liu Y, Peng G, Zhou J, Xue S, Wang R, Tang Y, Meng X, Pei G, Bai Y, Liu Q, Li H, Zhang J. Prevalence of psoriasis in China: a population-based study in six cities. Eur J Dermatol. 2012; 22(5): 663-7.	Shanxi	2009-2011
	Ding X, Wang T, Shen Y, Wang X, Zhou C, Tian S, Liu Y, Peng G, Zhou J, Xue S, Wang R, Tang Y, Meng X, Pei G, Bai Y, Liu Q, Li H, Zhang J. Prevalence of psoriasis in China: a population-based study in six cities. Eur J Dermatol. 2012; 22(5): 663-7.	Sichuan	2009-2011
	Perera A, Atukorale DN, Sivayogan S, Ariyaratne VS, Karunaratne LDA. Prevalence of skin diseases in suburban Sri Lanka. Ceylon Med J. 2000; 45(3): 123-8.	Sri Lanka	1997
	Lindberg M, Isacson D, Bingefors K. Self-reported skin diseases, quality of life and medication use: a nationwide pharmaco-epidemiological survey in Sweden. Acta Derm Venereol. 2014; 94(2): 188-91.	Sweden	2004-2005
	Chang Y-T, Chen T-J, Liu P-C, Chen Y-C, Chen Y-J, Huang Y-L, Jih J-S, Chen C-C, Lee D-D, Wang W-J, Lin M-W, Liu H-N. Epidemiological study of psoriasis in the national health insurance database in Taiwan. Acta Derm. Venereol. 2009; 89(3): 262-6.	Taiwan	2000-2006
	Chen G-Y, Cheng Y-W, Wang C-Y, Hsu T-J, Hsu MM-L, Yang P-T, Chen W-C. Prevalence of skin diseases among schoolchildren in Magong, Penghu, Taiwan: a community-based clinical survey. J Formos Med Assoc. 2008; 107(1): 21-9.	Taiwan	2005
	Yang Y-C, Cheng Y-W, Lai C-S, Chen W. Prevalence of childhood acne, ephelides, warts, atopic dermatitis, psoriasis, alopecia areata and keloid in Kaohsiung County, Taiwan: a community-based clinical survey. J Eur Acad Dermatol Venereol. 2007; 21(5): 643-9.	Taiwan	2004
	Henderson CA. Skin disease in rural Tanzania. Int J Dermatol. 1996; 35(9): 640-2.	Tanzania	1991
	Satimia FT, McBride SR, Leppard B. Prevalence of skin disease in rural Tanzania and factors influencing the choice of health care, modern or traditional. Arch Dermatol. 1998; 134(11): 1363-6.	Tanzania	1996
	Tuncel AA, Erbagci Z. Prevalence of skin diseases among male adolescent and post-	Turkey	2004

© 2017 Karimkhani C et al. *JAMA Dermatology.*

	adolescent boarding school students inTurkey. J Dermatol. 2005; 32(7): 557-64.		
	Gelfand JM, Weinstein R, Porter SB, Neimann AL, Berlin JA, Margolis DJ. Prevalence and treatment of psoriasis in the United Kingdom: a population-based study. Arch Dermatol. 2005; 141(12): 1537-41.	United Kingdom	1987-2002
	Huerta C, Rivero E, Rodríguez LAG. Incidence and risk factors for psoriasis in the general population. Arch Dermatol. 2007; 143(12): 1559-65.	United Kingdom	1996-1997
	Nevitt GJ, Hutchinson PE. Psoriasis in the community: prevalence, severity and patients' beliefs and attitudes towards the disease. Br J Dermatol. 1996; 135(4): 533-7.	United Kingdom	1995
	Rea JN, Newhouse ML, Halil T. Skin disease in Lambeth. A community study of prevalence and use of medical care. Br J Prev Soc Med. 1976; 30(2): 107-14.	United Kingdom	1976
	Steele K. Primary dermatological care in general practice. J R Coll Gen Pract. 1984; 34(258): 22-3.	United Kingdom	1983
	Beauregard S, Gilchrest BA. A survey of skin problems and skin care regimens in the elderly. Arch Dermatol. 1987; 123(12): 1638-43.	United States	1985-1987
	Bell LM, Sedlack R, Beard CM, Perry HO, Michet CJ, Kurland LT. Incidence of psoriasis in Rochester, Minn, 1980-1983. Arch Dermatol. 1991; 127(8): 1184-7.	United States	1980-1983
	Gelfand JM, Feldman SR, Stern RS, Thomas J, Rolstad T, Margolis DJ. Determinants of quality of life in patients with psoriasis: a study from the US population. J Am Acad Dermatol. 2004; 51(5): 704-8.	United States	2001
	Gelfand JM, Gladman DD, Mease PJ, Smith N, Margolis DJ, Nijsten T, Stern RS, Feldman SR, Rolstad T. Epidemiology of psoriatic arthritis in the population of the United States. J. Am. Acad. Dermatol. 2005; 53(4): 573.	United States	2001
	Icen M, Crowson CS, McEvoy MT, Dann FJ, Gabriel SE, Maradit Kremers H. Trends in incidence of adult-onset psoriasis over three decades: a population-based study. J Am Acad Dermatol. 2009; 60(3): 394-401.	United States	1979-1999
	Kurd SK, Gelfand JM. The prevalence of previously diagnosed and undiagnosed psoriasis in US adults: results from NHANES 2003-2004. J Am Acad Dermatol. 2009; 60(2): 218-24.	United States	2003-2004

© 2017 Karimkhani C et al. *JAMA Dermatology.*

	National Center for Health Statistics (NCHS), Centers for Disease Control and Prevention (CDC). United States National Health and Nutrition Examination Survey 2005-2006. Hyattsville, United States: National Center for Health Statistics (NCHS), Centers for Disease Control and Prevention (CDC), 2007.	United States	2005-2006
	Shbeeb M, Uramoto KM, Gibson LE, O'Fallon WM, Gabriel SE. The epidemiology of psoriatic arthritis in Olmsted County, Minnesota, USA, 1982-1991. J Rheumatol. 2000; 27(5): 1247-50.	United States	1982-1992
	Tollefson MM, Crowson CS, McEvoy MT, Maradit Kremers H. Incidence of psoriasis in children: a population-based study. J Am Acad Dermatol. 2010; 62(6): 979-87.	United States	1970-1999
	Wilson FC, Icen M, Crowson CS, McEvoy MT, Gabriel SE, Kremers HM. Incidence and clinical predictors of psoriatic arthritis in patients with psoriasis: a population-based study. Arthritis Rheum. 2009; 61(2): 233-9.	United States	1970-1999
	Wilson FC, Icen M, Crowson CS, McEvoy MT, Gabriel SE, Kremers HM. Time trends in epidemiology and characteristics of psoriatic arthritis over 3 decades: a population-based study. J. Rheumatol. 2009; 36(2): 361-7.	United States	1970-1999
Scabies	Clucas DB, Carville KS, Connors C, Currie BJ, Carapetis JR, Andrews RM. Disease burden and health-care clinic attendances for young children in remote aboriginal communities of northern Australia. Bull World Health Organ. 2008; 86(4): 275-81.	Australia	2002-2005
	Lapeere H, Naeyaert J-M, De Weert J, De Maeseneer J, Brochez L. Incidence of scabies in Belgium. Epidemiol Infect. 2008; 136(3): 395-8.	Belgium	2004
	Bechelli LM, Haddad N, Pimenta WP, Pagnano PM, Melchior E Jr, Fregnan RC, Zanin LC, Arenas A. Epidemiological survey of skin diseases in schoolchildren living in the Purus Valley (Acre State, Amazonia, Brazil). Dermatologica. 1981; 163(1): 78-93.	Brazil	1974-1975
	Feldmeier H, Jackson A, Ariza L, Calheiros CML, Soares V de L, Oliveira FA, Hengge UR, Heukelbach J. The epidemiology of scabies in an impoverished community in rural Brazil: presence and severity of disease are associated with poor living conditions and illiteracy. J Am Acad	Brazil	2003

© 2017 Karimkhani C et al. *JAMA Dermatology.*

	Dermatol. 2009; 60(3): 436-43.		
	Heukelbach J, Wilcke T, Winter B, Feldmeier H. Epidemiology and morbidity of scabies and pediculosis capitis in resource-poor communities in Brazil. Br J Dermatol. 2005; 153(1): 150-6.	Brazil	2001
	Abdel-Hafez K, Abdel-Aty MA, Hofny ERM. Prevalence of skin diseases in rural areas of Assiut Governorate, Upper Egypt. Int J Dermatol. 2003; 42(11): 887-92.	Egypt	1994-1996
	Ali J, Yifru S, Woldeamanuel Y. Prevalence of tinea capitis and the causative agent among school children in Gondar, North West Ethiopia. Ethiop Med J. 2011; 47(4): 261-9.	Ethiopia	2007-2008
	Dagnew MB, Günther E. Epidemiology of communicable skin diseases in school children of a rural area in North Ethiopia. Dermatol Monatsschr. 1990; 176(176): 219-23.	Ethiopia	1989
	Leekassa R, Bizuneh E, Alem A, Fekadu A, Shibre T. Community diagnosis of common skin diseases in the Zay community of the Zeway Islands, Ethiopia. Ethiop Med J. 2005; 43(3): 189-95.	Ethiopia	1998
	Woldeamanuel Y, Leekassa R, Chryssanthou E, Menghistu Y, Petrini B. Prevalence of tinea capitis in Ethiopian schoolchildren. Mycoses. 2005; 48(2): 137-41.	Ethiopia	2003-2005
	Steer AC, Jenney AWJ, Kado J, Batzloff MR, La Vincente S, Waqatakirewa L, Mulholland EK, Carapetis JR. High burden of impetigo and scabies in a tropical country. PLoS Negl Trop Dis. 2009; 3(6): e467.	Fiji	2006-2007
	Steer AC, Tikoduadua LV, Manalac EM, Colquhoun S, Carapetis JR, Maclennan C. Validation of an Integrated Management of Childhood Illness algorithm for managing common skin conditions in Fiji. Bull World Health Organ. 2009; 87(3): 173-9.	Fiji	2007
	Kramkimel N, Soussan V, Beauchet A, Duhamel A, Saiag P, Chevallier B, Mahé E. High frequency, diversity and severity of skin diseases in a paediatric emergency department. J Eur Acad Dermatol Venereol. 2010; 24(12): 1468-75.	France	2006
	Hogewoning A, Amoah A, Bavinck JNB, Boakye D, Yazdanbakhsh M, Adegnika A, De Smedt S, Fonteyne Y, Willemze R, Lavrijsen A. Skin diseases among schoolchildren in Ghana, Gabon, and Rwanda. Int J Dermatol. 2013; 52(5): 589-600.	Gabon	2005

© 2017 Karimkhani C et al. *JAMA Dermatology.*

	Frese T, Herrmann K, Sandholzer H. Pruritus as Reason for Encounter in General Practice. J Clin Med Res. 2011; 3(5): 223-9.	Germany	1999-2000
	Hogewoning A, Amoah A, Bavinck JNB, Boakye D, Yazdanbakhsh M, Adegnika A, De Smedt S, Fonteyne Y, Willemze R, Lavrijsen A. Skin diseases among schoolchildren in Ghana, Gabon, and Rwanda. Int J Dermatol. 2013; 52(5): 589-600.	Ghana	2004
	Hogewoning A, Amoah A, Bavinck JNB, Boakye D, Yazdanbakhsh M, Adegnika A, De Smedt S, Fonteyne Y, Willemze R, Lavrijsen A. Skin diseases among schoolchildren in Ghana, Gabon, and Rwanda. Int J Dermatol. 2013; 52(5): 589-600.	Ghana	2007
	Fung WK, Lo KK. Prevalence of skin disease among school children and adolescents in a Student Health Service Center in Hong Kong. Pediatr Dermatol. 2000; 17(6): 440-6.	Hong Kong Special Administration of China	1996-1997
	Grills N, Grills C, Spelman T, Stoove M, Hellard M, El-Hayek C, Singh R. Prevalence survey of dermatological conditions in mountainous north India. Int J Dermatol. 2012; 51(5): 579-87.	India	2010
	Grover S, Ranyal RK, Bedi MK. A cross section of skin diseases in rural Allahabad. Indian J Dermatol. 2008; 53(4): 179-81.	India	2005
	Libu GK, Bina T, Raphael L, Balakrishnan SE, Biju G, Samson JF, Bindu V. Prevalence and socio-demographic determinants of skin disease among lower primary school children in Calicut, Kerala. IMAKMJ. 2010; 185-90.	India	2006
	Patel JK, Vyas AP, Berman B, Vierra M. Incidence of childhood dermatosis in India. Skinmed. 2010; 8(3): 136-42.	India	2000-2002
	Lee J, Koh D, Andijani M, Saw SM, Munoz C, Chia SE, Wong ML, Hong CY, Ong CN. Effluents from a pulp and paper mill: a skin and health survey of children living in upstream and downstream villages. Occup Environ Med. 2002; 59(6): 373-9.	Indonesia	1999
	Al-Rubiay KK, Al-Rubaiy LK. Dermatoepidemiology: A Household Survey Among Two Urban Areas In Basrah City, Iraq. The Internet Journal of Dermatology. 2006; 4(2): 10.	Iraq	2005
	Schmeller W, Dzikus A. Skin diseases in children in rural Kenya: long-term results of a dermatology project within the primary health care system. Br J Dermatol. 2001; 144(1): 118-24.	Kenya	1993, 1999
	Mahé A, Prual A, Konaté M, Bobin P. Skin diseases	Mali	1993-

© 2017 Karimkhani C et al. *JAMA Dermatology.*

	of children in Mali: a public health problem. Trans R Soc Trop Med Hyg. 1995; 89(5): 467-70.		1994
	Hay RJ, Castanon RE, Hernandez HA, Lopez GC, Fuentes LF, Solis SP, Andersson N. Wastage of family income on skin disease in Mexico. BMJ. 1994; 309(6958): 848.	Mexico	1993
	Walker SL, Shah M, Hubbard VG, Pradhan HM, Ghimire M. Skin disease is common in rural Nepal: results of a point prevalence study. Br J Dermatol. 2008; 158(2): 334-8.	Nepal	2006
	Emodi IJ, Ikefuna AN, Uchendu U, Duru A. Skin diseases among children attending the out patient clinic of the University of Nigeria teaching hospital, Enug. Afr Health Sci. 2010; 10(4).	Nigeria	1996-2005
	Ogunbiyi AO, Omigbodun Y, Owoaje E. Prevalence of skin disorders in school children in southwest Nigeria. Int J Adolesc Med Health. 2009; 21(2): 235-41.	Nigeria	2002-2004, 2006-2008
	Oyedeji O, Okeniyi J, Ogunlesi T, Onayemi O, Oyedeji G, Oyelami O. Parental factors influencing the prevalence of skin infections and infestations among Nigerian primary school pupils. The Internet Journal of Dermatology. 2006; 3(2): 6.	Nigeria	2003
	Amro A, Hamarsheh O. Epidemiology of scabies in the West Bank, Palestinian Territories (Occupied). Int J Infect Dis. 2012; 16(2): e117-120.	Palestine	2005-2010
	Popescu R, Popescu CM, Williams HC, Forsea D. The prevalence of skin conditions in Romanian school children. Br J Dermatol. 1999; 140(5): 891-6.	Romania	1995
	Ibragimov SI. The structure of skin pathology in middle-aged and elderly subjects. Vestn Dermatol Venerol. 1990; 2: 37-40.	Romania	1989-1990
	Hogewoning A, Amoah A, Bavinck JNB, Boakye D, Yazdanbakhsh M, Adegnika A, De Smedt S, Fonteyne Y, Willemze R, Lavrijsen A. Skin diseases among schoolchildren in Ghana, Gabon, and Rwanda. Int J Dermatol. 2013; 52(5): 589-600.	Rwanda	2007
	Amin TT, Ali A, Kaliyadan F. Skin disorders among male primary school children in Al Hassa, Saudi Arabia: prevalence and socio-demographic correlates--a comparison of urban and rural populations. Rural Remote Health. 2011; 11(1): 1517.	Saudi Arabia	2009-2010
	Eason RJ, Tasman-Jones T. Resurgent yaws and other skin diseases in the Western Province of the	Solomon Islands	1984

© 2017 Karimkhani C et al. *JAMA Dermatology.*

	Solomon Islands. P N G Med J. 1985; 28(4): 247-50.		
	Perera A, Atukorale DN, Sivayogan S, Ariyaratne VS, Karunaratne LDA. Prevalence of skin diseases in suburban Sri Lanka. Ceylon Med J. 2000; 45(3): 123-8.	Sri Lanka	1997
	Chen G-Y, Cheng Y-W, Wang C-Y, Hsu T-J, Hsu MM-L, Yang P-T, Chen W-C. Prevalence of skin diseases among schoolchildren in Magong, Penghu, Taiwan: a community-based clinical survey. J Formos Med Assoc. 2008; 107(1): 21-9.	Taiwan	2005
	Wu YH, Su HY, Hsieh YJ. Survey of infectious skin diseases and skin infestations among primary school students of Taitung County, eastern Taiwan. J Formos Med Assoc. 2000; 99(2): 128-34.	Taiwan	1998
	Ferié J, Dinkela A, Mbata M, Idindili B, Schmid-Grendelmeier P, Hatz C. Skin disorders among school children in rural Tanzania a nd an assessment of therapeutic needs. Trop Doct. 2006; 36(4): 219-21.	Tanzania	2003
	Gibbs, S. Skin disease and socioeconomic conditions in rural Africa: Tanzania. Int J Dermatol. 1996; 35(9): 633-9.	Tanzania	1993-10995
	Henderson CA. Skin disease in rural Tanzania. Int J Dermatol. 1996; 35(9): 640-2.	Tanzania	1991
	Komba EV, Mgonda YM. The spectrum of dermatological disorders among primary school children in Dar es Salaam. BMC Public Health. 2010; 10(1): 765.	Tanzania	2007-2009
	Dos Santos MM, Amaral S, Harmen SP, Joseph HM, Fernandes JL, Counahan ML. The prevalence of common skin infections in four districts in Timor-Leste: a cross sectional survey. BMC Infect Dis. 2010; 61.	Timor-Leste	2007
	Pannell RS, Fleming DM, Cross KW. The incidence of molluscum contagiosum, scabies and lichen planus. Epidemiol Infect. 2005; 133(6): 985-91.	United Kingdom	1994-2003
	Analytical Sciences, Inc., National Center for Health Statistics (NCHS), Centers for Disease Control and Prevention (CDC), US Census Bureau. United States National Ambulatory Medical Care Survey 1995. Hyattsville, United States: National Center for Health Statistics (NCHS), Centers for Disease Control and Prevention (CDC).	United States	1995-2001
	Constella Group, National Center for Health	United	2002-

© 2017 Karimkhani C et al. *JAMA Dermatology.*

	Statistics (NCHS), Centers for Disease Control and Prevention (CDC), US Census Bureau. United States National Ambulatory Medical Care Survey 2002. Hyattsville, United States: National Center for Health Statistics (NCHS), Centers for Disease Control and Prevention (CDC).	States	2006
	Constella Group, National Center for Health Statistics (NCHS), Centers for Disease Control and Prevention (CDC), US Census Bureau. United States National Hospital Ambulatory Medical Care Survey 2002. Hyattsville, United States: National Center for Health Statistics (NCHS), Centers for Disease Control and Prevention (CDC).	United States	2002-2010
	National Center for Health Statistics (NCHS), Centers for Disease Control and Prevention (CDC), SRA International, Inc., US Census Bureau. United States National Hospital Ambulatory Medical Care Survey 2007. Hyattsville, United States: National Center for Health Statistics (NCHS), Centers for Disease Control and Prevention (CDC).	United States	2007-2010
	National Center for Health Statistics (NCHS), Centers for Disease Control and Prevention (CDC), US Census Bureau. United States National Ambulatory Medical Care Survey 1993. Hyattsville, United States: National Center for Health Statistics (NCHS), Centers for Disease Control and Prevention (CDC).	United States	1991-1994
	Harris M, Nako D, Hopkins T, Powell DM, Kenny C, Carroll C, Carroll K. Skin infections in Tanna, Vanuatu in 1989. P N G Med J. 1992; 35(2): 137-43.	Vanuatu	1989
	Paek SY, Koriakos A, Saxton-Daniels S, Pandya AG. Skin diseases in rural Yucatan, Mexico. Int J Dermatol. 2012; 51(7): 823-8.	Yucatan	2009-2010
Urticaria	Kilkenny M, Stathakis V, Jolley D, Marks R. Maryborough skin health survey: prevalence and sources of advice for skin conditions. Australas J Dermatol. 1998; 39(4): 233-7.	Australia	1996
	Canadian Institute for Health Information (CIHI). Canada National Ambulatory Care Reporting System. Ottawa, Canada: Canadian Institute for Health Information (CIHI).	Canada	2002-2009
	Kjaer HF, Eller E, Høst A, Andersen KE, Bindslev-Jensen C. The prevalence of allergic diseases in an unselected group of 6-year-old children. The DARC birth cohort study. Pediatr Allergy Immunol. 2008;	Denmark	1998-1999

	19(8): 737-45.		
	Frese T, Herrmann K, Sandholzer H. Pruritus as Reason for Encounter in General Practice. J Clin Med Res. 2011; 3(5): 223-9.	Germany	1999-2000
	Zuberbier T, Balke M, Worm M, Edenharter G, Maurer M. Epidemiology of urticaria: a representative cross-sectional population survey. Clinical and experimental dermatology. 2010; 35(8): 869-73.	Germany	2009-2010
	Konstantinou GN, Papadopoulos NG, Tavladaki T, Tsekoura T, Tsilimigaki A, Grattan CEH. Childhood acute urticaria in northern and southern Europe shows a similar epidemiological pattern and significant meteorological influences. Pediatr Allergy Immunol. 2011; 22(1 Pt 1): 36-42.	Greece	2005-2007
	Fung WK, Lo KK. Prevalence of skin disease among school children and adolescents in a Student Health Service Center in Hong Kong. Pediatr Dermatol. 2000; 17(6): 440-6.	Hong Kong Special Administrative Region of China	1996-1997
	Naldi L, Colombo P, Placchesi EB, Piccitto R, Chatenoud L, La Vecchia C. Study design and preliminary results from the pilot phase of the PraKtis study: self-reported diagnoses of selected skin diseases in a representative sample of the Italian population. Dermatology (Basel). 2004; 208(1): 38-42.	Italy	2003
	Becerril Angeles M, Vázquez Merino CL, Angeles Garay U, Alvarado Moctezuma LE, Vilchis Guízar E. Prevalence of allergic diseases in the elderly. Rev Alerg Mex. 2008; 55(3): 85-91.	Mexico	2006-2008
	Walker SL, Shah M, Hubbard VG, Pradhan HM, Ghimire M. Skin disease is common in rural Nepal: results of a point prevalence study. Br J Dermatol. 2008; 158(2): 334-8.	Nepal	2006
	Massa A, Alves R, Amado J, Matos E, Sanches M, Selores M, Santos C, Costa V, Velho G, Oliveira M, Ferreira E, Taveira M, Silva NS, Granado E, Lemos A, Calheiros JM. Prevalence of cutaneous lesions in Freixo de Espada à Cinta. Acta médica portuguesa. 2000; 13(5-6): 247-54.	Portugal	1998
	Popescu R, Popescu CM, Williams HC, Forsea D. The prevalence of skin conditions in Romanian school children. Br J Dermatol. 1999; 140(5): 891-6.	Romania	1995
	Al-Saeed WY, Al-Dawood KM, Bukhari IA,	Saudi	2003

© 2017 Karimkhani C et al. *JAMA Dermatology.*

	Bahnassy AA. Prevalence and pattern of skin disorders among female schoolchildren in Eastern Saudi Arabia. Saudi Med J. 2006; 27(2): 227-34.	Arabia	
	Amin TT, Ali A, Kaliyadan F. Skin disorders among male primary school children in Al Hassa, Saudi Arabia: prevalence and socio-demographic correlates-a comparison of urban and rural populations. Rural Remote Health. 2011; 11(1517).	Saudi Arabia	2009
	Konstantinou GN, Papadopoulos NG, Tavladaki T, Tsekoura T, Tsilimigaki A, Grattan CEH. Childhood acute urticaria in northern and southern Europe shows a similar epidemiological pattern and significant meteorological influences. Pediatr Allergy Immunol. 2011; 22(1 Pt 1): 36-42.	South East England	2005-2007
	Gaig P, Olona M, Munoz Lejarazu D, Caballero MT, Domínguez FJ, Echechipia S, Garcia Abujeta JL, Gonzalo MA, Lleonart R, Martinez Cocera C, Rodriguez A, Ferrer M. Epidemiology of urticaria in Spain. J Investig Allergol Clin Immunol. 2004; 14(3): 214-20.	Spain	2002-2004
	Perera A, Atukorale DN, Sivayogan S, Ariyaratne VS, Karunaratne LDA. Prevalence of skin diseases in suburban Sri Lanka. Ceylon Med J. 2000; 45(3): 123-8.	Sri Lanka	1997
	Gibbs, S. Skin disease and socioeconomic conditions in rural Africa: Tanzania. Int J Dermatol. 1996; 35(9): 633-9.	Tanzania	1995
	Analytical Sciences, Inc., National Center for Health Statistics (NCHS), Centers for Disease Control and Prevention (CDC), US Census Bureau. United States National Ambulatory Medical Care Survey 1995. Hyattsville, United States: National Center for Health Statistics (NCHS), Centers for Disease Control and Prevention (CDC).	United States	1995-2001
	Analytical Sciences, Inc., National Center for Health Statistics (NCHS), Centers for Disease Control and Prevention (CDC), US Census Bureau. United States National Hospital Ambulatory Medical Care Survey 1994. Hyattsville, United States: National Center for Health Statistics (NCHS), Centers for Disease Control and Prevention (CDC).	United States	1994-2001
	Constella Group, National Center for Health Statistics (NCHS), Centers for Disease Control and Prevention (CDC), US Census Bureau. United States National Ambulatory Medical Care Survey	United States	2002-2006

	2002. Hyattsville, United States: National Center for Health Statistics (NCHS), Centers for Disease Control and Prevention (CDC).		
	Constella Group, National Center for Health Statistics (NCHS), Centers for Disease Control and Prevention (CDC), US Census Bureau. United States National Hospital Ambulatory Medical Care Survey 2002. Hyattsville, United States: National Center for Health Statistics (NCHS), Centers for Disease Control and Prevention (CDC).	United States	2002-2010
	National Center for Health Statistics (NCHS), Centers for Disease Control and Prevention (CDC), SRA International, Inc., US Census Bureau. United States National Hospital Ambulatory Medical Care Survey 2007. Hyattsville, United States: National Center for Health Statistics (NCHS), Centers for Disease Control and Prevention (CDC).	United States	2007-2010
	Paek SY, Koriakos A, Saxton-Daniels S, Pandya AG. Skin diseases in rural Yucatan, Mexico. Int J Dermatol. 2012; 51(7): 823-8.	Yucatan	2009-2010
Viral skin diseases	Canadian Institute for Health Information (CIHI). Canada National Ambulatory Care Reporting System 2003-2004. Ottawa, Canada: Canadian Institute for Health Information (CIHI).	Canada	2003-2009
	Abdel-Hafez K, Abdel-Aty MA, Hofny ERM. Prevalence of skin diseases in rural areas of Assiut Governorate, Upper Egypt. Int J Dermatol. 2003; 42(11): 887-92.	Egypt	1994-1996
	Yamamah GA, Emam HM, Abdelhamid MF, Elsaie ML, Shehata H, Farid T, Kamel MI, Taalat AA. Epidemiologic study of dermatologic disorders among children in South Sinai, Egypt. Int J Dermatol. 2012; 51(10): 1180-5.	Egypt	2008-2009
	Dagnew MB, Günther E. Epidemiology of communicable skin diseases in school children of a rural area in North Ethiopia. Dermatol Monatsschr. 1990; 176(176): 219-23.	Ethiopia	1989
	Murgia V, Bilcha KD, Shibeshi D. Community dermatology in Debre Markos: an attempt to define children's dermatological needs in a rural area of Ethiopia. Int J Dermatol. 2010; 49(6): 666-71.	Ethiopia	2009
	Woldeamanuel Y, Leekassa R, Chryssanthou E, Menghistu Y, Petrini B. Prevalence of tinea capitis in Ethiopian schoolchildren. Mycoses. 2005; 48(2): 137-41.	Ethiopia	2003-2005
	Woldeamanuel Y, Mengistu Y, Chryssanthou	Ethiopia	2001

© 2017 Karimkhani C et al. *JAMA Dermatology.*

	E, Petrini B.<br>Dermatophytosis in Tulugudu Island, Ethiopia. Med Mycol. 2005; 43(1): 79-82.		
	Hogewoning A, Amoah A, Bavinck JNB, Boakye D, Yazdanbakhsh M, Adegnika A, De Smedt S, Fonteyne Y, Willemze R, Lavrijsen A. Skin diseases among schoolchildren in Ghana, Gabon, and Rwanda. Int J Dermatol. 2013; 52(5): 589-600.	Gabon	2004, 2005, 2007
	Grills N, Grills C, Spelman T, Stoove M, Hellard M, El-Hayek C, Singh R. Prevalence survey of dermatological conditions in mountainous north India. Int J Dermatol. 2012; 51(5): 579-87.	India	2010
	Grover S, Ranyal RK, Bedi MK. A cross section of skin diseases in rural Allahabad. Indian J Dermatol. 2008; 53(4): 179-81.	India	2005
	Libu GK, Bina T, Raphael L, Balakrishnan SE, Biju G, Samson JF, Bindu V. Prevalence and socio-demographic determinants of skin disease among lower primary school children in Calicut, Kerala. IMAKMJ. 2010; 185-90.	India	2006
	Patel JK, Vyas AP, Berman B, Vierra M. Incidence of childhood dermatosis in India. Skinmed. 2010; 8(3): 136-42.	India	2000-2002
	Lee J, Koh D, Andijani M, Saw SM, Munoz C, Chia SE, Wong ML, Hong CY, Ong CN. Effluents from a pulp and paper mill: a skin and health survey of children living in upstream and downstream villages. Occup Environ Med. 2002; 59(6): 373-9.	Indonesia	1999
	Niizeki K, Kano O, Kondo Y.<br>An epidemic study of molluscum contagiosum. Relationship to swimming. Dermatologica. 1984; 169(4): 197-8.	Japan	1982-1983
	Mahé A, Prual A, Konaté M, Bobin P. Skin diseases of children in Mali: a public health problem. Trans R Soc Trop Med Hyg. 1995; 89(5): 467-70.	Mali	1993-1994
	Hay RJ, Castanon RE, Hernandez HA, Lopez GC, Fuentes LF, Solis SP, Andersson N. Wastage of family income on skin disease in Mexico. BMJ. 1994; 309(6958): 848.	Mexico	1993
	Walker SL, Shah M, Hubbard VG, Pradhan HM, Ghimire M. Skin disease is common in rural Nepal: results of a point prevalence study. Br J Dermatol. 2008; 158(2): 334-8.	Nepal	2006
	Koning S, Bruijnzeels MA, van Suijlekom-Smit LW, van der Wouden JC. Molluscum contagiosum in Dutch general practice. Br J Gen	Netherlands	1987-1988

© 2017 Karimkhani C et al. *JAMA Dermatology.*

	Pract. 1994; 44(386): 417-9.		
	Mohammedamin RS, van der Wouden JC, Koning S, van der Linden MW, Schellevis FG, van Suijlekom-Smit LW, Koes BW. Increasing incidence of skin disorders in children? A comparison between 1987 and 2001. BMC Dermatol. 2006; 6(4).	Netherlands	1987, 2001
	Mohammedamin RS, van der Wouden JC, Koning S, van der Linden MW, Schellevis FG, van Suijlekom-Smit LW, Koes BW. Self-reported prevalence of warts in children and GP consultation. Eur J Gen Pract. 2008; 14(1): 34-6.	Netherlands	2001
	Van Haalen FM, Bruggink SC, Gussekloo J, Assendelft WJJ, Eekhof JAH. Warts in primary schoolchildren: prevalence and relation with environmental factors. Br J Dermatol. 2009; 161(1): 148-52.	Netherlands	2007
	Oyedeji O, Okeniyi J, Ogunlesi T, Onayemi O, Oyedeji G, Oyelami O. Parental factors influencing the prevalence of skin infections and infestations among Nigerian primary school pupils. Internet J Dermatol. 2006; 3(2): 6.	Nigeria	2003
	Dalgard F, Svensson A, Holm JØ, Sundby J. Self-reported skin morbidity in Oslo. Associations with sociodemographic factors among adults in a cross-sectional study. BMC Dermatol. 2004; 6(4): 452-7.	Norway	2000-2001
	Gutierrez E, Galarza C, Ramos W, Tello M, Rojas I, Chia H, Ronceros G, Ortega-Loayza A. Prevalence of skin diseases in a rural area of Peruvian Amazonia. Dermatol Peru. 2009; 19(2): 104-12.	Peru	2005
	Massa A, Alves R, Amado J, Matos E, Sanches M, Selores M, Santos C, Costa V, Velho G, Oliveira M, Ferreira E, Taveira M, Silva NS, Granado E,Lemos A, Calheiros JM. Prevale?ncia das leso?es cuta?neas em Freixo de Espada a? Cinta. Acta Med Port. 2000; 13((5-6)): 247-54.	Portugal	1998
	Popescu R, Popescu CM, Williams HC, Forsea D. The prevalence of skin conditions in Romanian school children. Br J Dermatol. 1999; 140(5): 891-6.	Romania	1995
	Hogewoning A, Amoah A, Bavinck JNB, Boakye D, Yazdanbakhsh M, Adegnika A, De Smedt S, Fonteyne Y, Willemze R, Lavrijsen A. Skin diseases among schoolchildren in Ghana, Gabon,	Rwanda	2007

© 2017 Karimkhani C et al. *JAMA Dermatology.*

	and Rwanda. Int J Dermatol. 2013; 52(5): 589-600.		
	Amin TT, Ali A, Kaliyadan F. Skin disorders among male primary school children in Al Hassa, Saudi Arabia: prevalence and socio-demographic correlates--a comparison of urban and rural populations. Rural Remote Health. 2011; 11(1): 1517.	Saudi Arabia	2009
	Bahamdan K, Mahfouz AA, Tallab T, Badawi IA, al-Amari OM. Skin diseases among adolescent boys in Abha, Saudi Arabia. Int J Dermatol. 1996; 35(6): 405-7.	Saudi Arabia	1995-1996
	Perera A, Atukorale DN, Sivayogan S, Ariyaratne VS, Karunaratne LDA. Prevalence of skin diseases in suburban Sri Lanka. Ceylon Med J. 2000; 45(3): 123-8.	Sri Lanka	1997
	Chen G-Y, Cheng Y-W, Wang C-Y, Hsu T-J, Hsu MM-L, Yang P-T, Chen W-C. Prevalence of skin diseases among schoolchildren in Magong, Penghu, Taiwan: a community-based clinical survey. J Formos Med Assoc. 2008; 107(1): 21-9.	Taiwan	2005
	Yang Y-C, Cheng Y-W, Lai C-S, Chen W. Prevalence of childhood acne, ephelides, warts, atopic dermatitis, psoriasis, alopecia areata and keloid in Kaohsiung County, Taiwan: a community-based clinical survey. J Eur Acad Dermatol Venereol. 2007; 21(5): 643-9.	Taiwan	2004
	Gibbs, S. Skin disease and socioeconomic conditions in rural Africa: Tanzania. Int J Dermatol. 1996; 35(9): 633-9.	Tanzania	2005
	Henderson CA. Skin disease in rural Tanzania. Int J Dermatol. 1996; 35(9): 640-2.	Tanzania	1991
	Komba EV, Mgonda YM. The spectrum of dermatological disorders among primary school children in Dar es Salaam. BMC Public Health. 2010; 10(1): 765.	Tanzania	2007-2010
	Pannell RS, Fleming DM, Cross KW. The incidence of molluscum contagiosum, scabies and lichen planus. Epidemiol Infect. 2005; 133(6): 985-91.	United Kingdom	1994-2003
	Royal College of General Practitioners. United Kingdom - England and Wales Weekly Returns Service Annual Report 2005. Royal College of General Practitioners.	United Kingdom	2005
	Agency for Healthcare Research and Quality. United States Medical Expenditure Panel Survey 1996-2011. Agency for Healthcare Research and	United States	2010

© 2017 Karimkhani C et al. *JAMA Dermatology.*

	Quality.		
	Analytical Sciences, Inc., National Center for Health Statistics (NCHS), Centers for Disease Control and Prevention (CDC), US Census Bureau. United States National Ambulatory Medical Care Survey. Hyattsville, United States: National Center for Health Statistics (NCHS), Centers for Disease Control and Prevention (CDC).	United States	1995-2001
	Analytical Sciences, Inc., National Center for Health Statistics (NCHS), Centers for Disease Control and Prevention (CDC), US Census Bureau. United States National Hospital Ambulatory Medical Care Survey. Hyattsville, United States: National Center for Health Statistics (NCHS), Centers for Disease Control and Prevention (CDC).	United States	1995-2001
	Constella Group, National Center for Health Statistics (NCHS), Centers for Disease Control and Prevention (CDC), US Census Bureau. United States National Ambulatory Medical Care Survey. Hyattsville, United States: National Center for Health Statistics (NCHS), Centers for Disease Control and Prevention (CDC).	United States	2002-2006
	Constella Group, National Center for Health Statistics (NCHS), Centers for Disease Control and Prevention (CDC), US Census Bureau. United States National Hospital Ambulatory Medical Care Survey. Hyattsville, United States: National Center for Health Statistics (NCHS), Centers for Disease Control and Prevention (CDC).	United States	2002-2006
	National Center for Health Statistics (NCHS), Centers for Disease Control and Prevention (CDC), SRA International, Inc., US Census Bureau. United States National Ambulatory Medical Care Survey. Hyattsville, United States: National Center for Health Statistics (NCHS), Centers for Disease Control and Prevention (CDC).	United States	2007-2010
	National Center for Health Statistics (NCHS), Centers for Disease Control and Prevention (CDC), SRA International, Inc., US Census Bureau. United States National Hospital Ambulatory Medical Care Survey. Hyattsville, United States: National Center for Health Statistics (NCHS), Centers for Disease Control and Prevention (CDC).	United States	2007-2009
	National Center for Health Statistics (NCHS), Centers for Disease Control and Prevention (CDC), US Census Bureau. United States National	United States	1995-2009

© 2017 Karimkhani C et al. *JAMA Dermatology.*

	Ambulatory Medical Care Survey 1995 and United States National Hospital Ambulatory Medical Care Survey.		
	Paek SY, Koriakos A, Saxton-Daniels S, Pandya AG. Skin diseases in rural Yucatan, Mexico. Int J Dermatol. 2012; 51(7): 823-8.	Yucatan	2009-2010
Melanoma AND NMSC	Aichi Cancer Center Research Institute. Japan Aichi Cancer Registry Report 2006	Japan	2006
	Ardabil University of Medical Sciences, Digestive Diseases Research Center (Iran), International Agency for Research on Cancer (IARC). Iran - Ardabil Cancer Registry Extracts 1985-2008.	Iran	1985-2008
	Association of Nordic Cancer Registries (ANCR). Denmark NORDCAN Cancer Incidence Data Tables, Age-Specific by Countries. Copenhagen, Denmark: Association of Nordic Cancer Registries (ANCR).	Denmark	
	Association of Nordic Cancer Registries (ANCR). Finland NORDCAN Cancer Incidence Data Tables, Age-Specific by Countries. Copenhagen, Denmark: Association of Nordic Cancer Registries (ANCR.	Finland	
	Association of Nordic Cancer Registries (ANCR). Iceland NORDCAN Cancer Incidence Data Tables, Age-Specific by Countries. Copenhagen, Denmark: Association of Nordic Cancer Registries (ANCR).	Iceland	
	Association of Nordic Cancer Registries (ANCR). Norway NORDCAN Cancer Incidence Data Tables, Age-Specific by Countries. Copenhagen, Denmark: Association of Nordic Cancer Registries (ANCR).	Norway	
	Association of Nordic Cancer Registries (ANCR). Sweden NORDCAN Cancer Incidence Data Tables, Age-Specific by Countries. Copenhagen, Denmark: Association of Nordic Cancer Registries (ANCR).	Sweden	
	Australasian Association of Cancer Registries, Australian Institute of Health and Welfare. Australia Cancer Incidence and Mortality Books 2012. Canberra, Australia: Australian Institute of Health and Welfare, 2012.	Australia	2012
	Bah E, Parkin DM, Hall AJ, Jack AD, Whittle H. Cancer in the Gambia: 1988-97. Br J Cancer. 2001; 84(9): 1207-14.	Gambia	1988-1997
	Banda LT, Parkin DM, Dzamalala CP, Liomba NG. Cancer incidence in Blantyre, Malawi 1994-1998. Trop Med Int Health. 2001; 6(4): 296-304.	Malawai	1994-1998
	Bavaria Population-Based Cancer Registry.	Germany	2004

© 2017 Karimkhani C et al. *JAMA Dermatology.*

	Germany - Bayern Population-Based Cancer Registry Report 2004. Erlangen, Germany: Bavaria Population-Based Cancer Registry, 2007.		
	Belgian Cancer Registry. Belgium Cancer Registry - Incidence. Brussels, Belgium: Belgian Cancer Registry.	Belgium	
	Brooks SE, Hanchard B, Wolff C, Samuels E, Allen J. Age-specific incidence of cancer in Kingston and St. Andrew, Jamaica, 1988-1992. West Indian Med J. 1995; 44(3): 102-5.	Jamaica	1988-1992
	Brooks SE, Wolff C. Age- specific incidence of cancer in Kingston and St. Andrew, Jamaica. Part I: 1978-1982. West Indian Med J. 1991; 40(3): 127-8.	Jamaica	1978-1982
	Bulgarian National Cancer Registry. Bulgaria Cancer Incidence Report 2006. Sofia, Bulgaria: "Avis 24" Ltd, 2008.	Bulgaria	2006
	Canary Islands Population-Based Cancer Registry. Spain - Canary Islands Population-Based Cancer Registry Report 2003-2004. Canarias: Canary Islands Population-Based Cancer Registry.	Canary Islands	2003-2004
	Cancer Association of Namibia, Namibian Cancer Registry. Namibia Cancer Registry Report 2000-2005. Windhoek, Namibia: Cancer Association of Namibia, 2009.	Namibia	2000-2005
	Cancer Control Department, Ministry of Health (Turkey). Turkey Active Cancer Registration System 8 Provinces Incidence 2007.	Turkey	2007
	Cancer Control Department, Ministry of Health (Turkey). Turkey Active Cancer Registration System 9 Provinces Incidence 2008.	Turkey	2008
	Cancer Control Department, Ministry of Health (Turkey). Turkey Cancer Statistics. Ankara, Turkey: Cancer Control Department, Ministry of Health (Turkey).	Turkey	2002-2005
	Cancer Registry of Republic of Slovenia, Institute of Oncology Ljubljana (Slovenia). Slovenia Cancer 2009. Ljubljana, Slovenia: Cancer Registry of Republic of Slovenia, 2013.	Slovenia	2009
	Cancer Registry of Republic of Slovenia. Slovenia Cancer. Ljubljana, Slovenia: Cancer Registry of Republic of Slovenia.	Slovenia	2007-2008
	Cancer Registry of Republic of Slovenia. Slovenia Cancer Incidence 2003. Ljubljana, Slovenia: Cancer Registry of Republic of Slovenia.	Slovenia	2003-2004, 2006
	Casablanca Ministry of Health (Morocco), Faculty of Medicine and Pharmacy of Casablanca	Morocco	2004

© 2017 Karimkhani C et al. *JAMA Dermatology.*

	(Morocco), Ibn Rochd University Hospital (Morocco), Lalla Salma Association to Fight Against Cancer (Morocco), National Institute of Oncology Sidi Mohamed Ben Abdellah Rabat (Morocco). Morocco - Cancer Registry of Greater Casablanca 2004. Rabat, Morocco: Lalla Salma Association to Fight Against Cancer (Morocco), 2007.		
	Center for Disease Control and Prevention, Ministry of Health and Medical Education (Iran). Iran National Cancer Registry Report.	Iran	2005-2007
	Chiang Mai University (Thailand), Chonburi Cancer Center (Thailand), Khon Kaen University (Thailand), Lampang Cancer Center (Thailand), Lopburi Cancer Center (Thailand), Ministry of Education (Thailand), National Cancer Institute (Thailand), Prince of Songkla University (Thailand), Surat Thani Cancer Center (Thailand), Ubon Ratchathani Cancer Center (Thailand), Udon Thani Cancer Center (Thailand). Thailand - Cancer in Thailand Volume V, 2001-2003. Bangkok, Thailand: National Cancer Institute (Thailand), 2010.	Thailand	2001-203
	Common Cancer Registry of the states of Berlin, Brandenburg, Mecklenburg-Western Pomerania, Saxony-Anhalt and the Free States of Saxony and Thuringia. Germany Common Cancer Registry Incidence and Mortality 2007-2008. Berlin, Germany: Common Cancer Registry of the states of Berlin, Brandenburg, Mecklenburg-Western Pomerania, Saxony-Anhalt and the Free States of Saxony and Thuringia, 2009, 2012.	Germany	2005-2008
	Czech National Cancer Registry. Czech Republic Cancer Incidence Report 2003. Prague, Czech Republic: Institute of Health Information and Statistics of the Czech Republic.	Czech Republic	2003-2004, 2006-2009
	Dr. Elizabeth Quamina Cancer Registry, Ministry of Health (Trinidad and Tobago). Trinidad and Tobago Cancer Incidence 1995-2006.	Trinidad and Tobago	1995-2006
	Echimane AK, Ahnoux AA, Adoubi I, Hien S, M'Bra K, D'Horpock A, Diomande M, Anongba D, Mensah-Adoh I, Parkin DM. Cancer Incidence in Abidjan, Ivory Coast 1995-1997. Cancer. 2000; 89(3): 653-63.	Ivory Coast	1995-1997
	El-Minia Cancer Center, Ministry of Communications and Information Technology	Egypt	2009

	(Egypt), Ministry of Health and Population (Egypt), National Cancer Registry Program of Egypt. Egypt - El-Minia National Cancer Registry Report 2009. Cairo, Egypt: National Cancer Registry Program of Egypt, 2011.		
	Ferlay J, Parkin DM, Curado MP, Bray F, Edwards B, Shin HR and Forman D. Cancer Incidence in Five Continents, Volumes I to IX: IARC CancerBase No. 9 [Internet]. Lyon, France: International Agency for Research on Cancer; 2010. Available from: http://ci5.iarc.fr.		
	Forman D, Bray F, Brewster DH, Gombe Mbalawa C, Kohler B, Piñeros M, Steliarova-Foucher E, Swaminathan R and Ferlay J, eds (2013). Cancer Incidence in Five Continents, Vol. X Summary Database (electronic version). Lyon, IARC. http://ci5.iarc.fr.		
	Greek Cancer Registry, Ministry of Health and Welfare (Greece). Greece Cancer Morbidity Report 1990-1991. Ministry of Health and Welfare (Greece), 1997.	Greece	199-1991
	Hamburg Cancer Registry (Germany). Germany - Hamburg Cancer Registry Incidence Tables. Hamburg, Germany: Hamburg Cancer Registry (Germany.	Germany	
	Hanchard B, Blake G, Wolff C, Samuels E, Waugh N, Simpson D, Ramjit C, Mitchell K. Age-specific incidence of cancer in Kingston and St. Andrew, Jamaica, 1993-1997. West Indian Med J. 2001; 50(2): 123-9.	Jamaica	1993-1997
	Honorary Commission for the Fight Against Cancer (Uruguay), National Cancer Registry (Uruguay). Uruguay Cancer Incidence Atlas 2002-2006. Montevideo, Uruguay: Honorary Commission for the Fight Against Cancer (Uruguay), 2010.	Uruguay	2002-2006
	Israel National Cancer Registry. Israel Cancer Incidence Tables 2008.	Israel	2008
	Israel National Cancer Registry. Israel Cancer Incidence Tables. Jerusalem, Israel: Ministry of Health (Israel).	Israel	2009-2010
	Italian Association of Cancer Registries (AIRTUM). Italy ITACAN Cancer Incidence Tables, AIRTUM Age-Specific Rates. Italy: Italian Association of Cancer Registries (AIRTUM).	Italy	
	Izmir Cancer Registry (KIDEM). Turkey - Izmir Cancer Registry Incidence 2006.	Turkey	2006

© 2017 Karimkhani C et al. *JAMA Dermatology.*

	Jordan Cancer Registry, Middle East Cancer Consortium, National Cancer Institute (United States). Jordan Cancer Incidence Report. Amman, Jordan: Ministry of Health (Jordan).	Jordan	2001-2008
	Kenya Medical Research Institute (KEMRI). Kenya - Nairobi Cancer Incidence Report 2000-2002. Kenya Medical Research Institute (KEMRI), 2006.	Kenya	2000-2002
	Korea Central Cancer Registry, Ministry of Health and Welfare (South Korea), National Cancer Center (South Korea). Annual Report of Cancer Statistics in Korea in 2010, 2012.	South Korea	2010, 2012
	Korea Central Cancer Registry, Ministry of Health and Welfare (South Korea). Annual Report of Cancer Statistics in Korea in 2008. Goyang-si, South Korea: National Cancer Center (South Korea), 2010.	South Korea	2008
	Korea Central Cancer Registry, Ministry of Health and Welfare (South Korea). KOSIS Database - Incidence of 61 Cancers by Sex and 5-Year Age Groups - Korean Statistical Information Service. Seoul, South Korea: Statistics Korea (South Korea).	South Korea	
	Koulibaly M, Kabba IS, Cissé A, Diallo SB, Diallo MB, Keita N, Camara ND, Diallo MS, Sylla BS, Parkin DM. Cancer incidence in Conakry, Guinea: first results from the Cancer Registry 1992-1995. Int J Cancer. 1997; 70(1): 39-45.	Guinea	1992-1995
	Lithuanian Cancer Registry. Lithuania Cancer Incidence - Females 2003.	Lithuania	2003
	Lithuanian Cancer Registry. Lithuania Cancer Incidence - Males 2003.	Lithuania	2003
	Lithuanian Cancer Registry. Lithuania Cancer Incidence. Vilnius, Lithuania: Institute of Oncology, Vilnius University (Lithuania).	Lithuania	2010-2011
	Malta National Cancer Registry. Malta Cancer Incidence and Mortality 1999-2010. Valletta, Malta: Department of Health Information and Information Research, Ministry for Health (Malta), 2012.	Malta	1999-2010
	Ministry of Health (Chile). Chile First Report of Population Cancer Registries 2003-2007. Santiago, Chile: Ministry of Health (Chile), 2012.	Chile	2003-2007
	Ministry of Health (New Zealand), New Zealand Cancer Registry. New Zealand Cancer: New Registrations and Deaths 2003. Wellington, New Zealand: Ministry of Health (New Zealand), 2007, 2010.	New Zealand	2003-2004, 2006-2007
	Ministry of Health (Oman), Oman National Cancer	Oman	2007

	Registry. Cancer Incidence in Oman 2007. Muscat, Oman: Ministry of Health (Oman).		
	Ministry of Health (Oman), Oman National Cancer Registry. Oman - Cancer Incidence in Oman 2002. Muscat, Oman: Ministry of Health (Oman).	Oman	2002-2006, 2008
	Ministry of Health (Palestine). Palestine Health Status Annual Report. Nablus, Palestine: Ministry of Health (Palestine), 2011, 2012.	Palestine	2010-2011
	Ministry of Health and Medical Education (Iran). Iran National Cancer Registry Report 2004-2005.	Iran	2004-2005
	Ministry of Health and Population (Egypt), National Cancer Registry Program of Egypt. Egypt - Aswan National Cancer Registry Report. Cairo, Egypt: National Cancer Registry Program of Egypt, 2010, 2011.	Egypt	2008-2009
	Ministry of Health, Population and Hospital Reform (Algeria), University Hospital of Batna. Algeria - Batna Cancer Registy Report 2000-2006. Batna, Algeria: University Hospital of Batna.	Algeria	2000-2006
	Ministry of Public Health (Lebanon). Lebanon National Cancer Registry Tables. Beirut, Lebanon: Ministry of Public Health (Lebanon).	Lebanon	2005-2007
	Modena Cancer Registry (Italy). Italy - Modena Cancer Registry Report 2003.	Italy	2003
	National Cancer Control Programme, Ministry of Health (Sri Lanka). Sri Lanka - Cancer Incidence Data: Sri Lanka Year 2001-2005. Colombo, Sri Lanka: National Cancer Control Programme, Ministry of Health (Sri Lanka), 2009.	Sri Lanka	2001-205
	National Cancer Registry (Hungary). Hungary National Cancer Registry - Incidence.	Hungary	2001-2011
	National Cancer Registry (Poland). Poland National Cancer Registry Incidence Tables by Site and Age Groups. Warsaw, Poland: Ministry of Health (Poland).	Poland	
	National Cancer Registry (Saudi Arabia). Saudi Arabia Cancer Incidence Report. Riyadh, Saudi Arabia: National Cancer Registry (Saudi Arabia).	Saudi Arabia	1994-2003
	National Cancer Registry (South Africa). Cancer in South Africa Full Report. Johannesburg, South Africa: National Institute for Occupational Health (South Africa).	South Africa	2003-2005
	National Cancer Registry Ireland. Ireland National Cancer Registry - Incidence. Cork, Ireland: National Cancer Registry Ireland.	Ireland	
	National Cancer Registry Programme (India). India	India	2005-

© 2017 Karimkhani C et al. *JAMA Dermatology.*

	- North East Population Based Cancer Registries Report 2005-2006. New Delhi, India: Indian Council of Medical Research (ICMR), 2008.		2006
	National Cancer Registry Programme (India). India Consolidated Report of Population Based Cancer Registries 2004-2005. New Delhi, India: Indian Council of Medical Research (ICMR), 2008.	India	200420 05
	National Cancer Registry Programme (India). India Population Based Cancer Registries 2009-2011. New Delhi, India: Indian Council of Medical Research (ICMR), 2013.	India	2009-2011
	National Cancer Registry Programme (India). India Three-Year Report of Population Based Cancer Registries 2006-2008. New Delhi, India: Indian Council of Medical Research (ICMR), 2010.	India	2006-2008
	National Cancer Registry, Ministry of Health (Malaysia). Malaysia - Second Report of the National Cancer Registry: Cancer Incidence in Malaysia 2003. Kuala Lumpur, Malaysia: National Cancer Registry, Ministry of Health (Malaysia), 2004.	Malaysia	2003
	National Central Cancer Registry (China). China Cancer Registry Incidence and Mortality 1990-2009.	China	1990-2009
	National Institute for Health Development (Estonia). Estonia New Cases of Malignant Neoplasms by Specified Site, Gender, and Age Group - Health Statistics and Health Research Database.	Estonia	
	National Registry of Diseases Office (NRDO), Ministry of Health (Singapore). Singapore Cancer Registry Incidence 2004-2008. Singapore, Singapore: National Registry of Diseases Office (NRDO), Ministry of Health (Singapore).	Singapore	2004-2008
	Netherlands Cancer Registry. Netherlands Cancer Registry Statistics - Incidence 1989-2012. Amsterdam, Netherlands: Comprehensive Cancer Center of the Netherlands (IKNL).	Netherlands	1989-2012
	New Brunswick Cancer Network, Department of Health. Canada - New Brunswick Cancer Registry Report 2002-2006. Fredericton, Canada: New Brunswick Cancer Network, Department of Health, 2010.	Canada	2002-2006
	Oran Cancer Registry (Algeria). Algeria - Oran Cancer Registry Report 2005. Oran, Algeria: Oran Cancer Registry (Algeria), 2006, 2007.	Algeria	2005-2006

© 2017 Karimkhani C et al. *JAMA Dermatology.*

	Paksoy N, Bouchardy C, Parkin DM. Cancer incidence in Western Samoa. Int J Epidemiol. 1991; 20(3): 634-41.	Samoa	1980-1988
	Parkin DM, International Agency for Research on Cancer, International Association of Cancer Registries. Cancer Incidence in Five Continents. Vol. I to VIII. Lyon, France, IARC Press, 2005.		
	PROMEC Unit, South African Medical Research Council. South Africa PROMEC Cancer Registry Incidence 1998-2002.	South Africa	1998-2002
	Rheinland-Pfalz Cancer Registry (Germany). Germany - Rheinland-Pfalz Cancer Incidence and Mortality 2003. Mainz, Germany: Rheinland-Pfalz Cancer Registry (Germany), 2006.	Germany	2003
	Saarland Cancer Registry (Germany). Germany - Saarland 40 Years of the Epidemiological Cancer Registry 2007. Saarbrücken, Germany: Saarland Ministry of Justice, 2007.	Germany	2007
	Sadjadi A, Malekzadeh R, Derakhshan MH, Sepehr A, Nouraie M, Sotoudeh M, Yazdanbod A, Shokoohi B, Mashayekhi A, Arshi S, Majidpour A, Babaei M, Mosavi A, Mohagheghi MMA, Alimohammadian M. Cancer Occurrence in Ardabil: Results of a Population-Based Cancer Registry from Iran. Int J Cancer. 2003; 107: 113–118.	Iran	1996-1999
	Sandagdorj T, Sanjaajamts E, Tudev U, Oyunchimeg D, Ochir C, Roder D. Cancer Incidence and Mortality in Mongolia - National Registry Data. Asian Pac J Cancer Prev. 2010; 11(6): 1509-14.	Monoglia	2003-2007
	Saskatchewan Cancer Agency. Canada - Saskatchewan Cancer Control Report, Profiling Cancer Prevalence 1984-2003. Regina, Canada: Saskatchewan Cancer Agency, 2008.	Canada	1984-2003
	Saudi Cancer Registry. Saudi Arabia Cancer Incidence Report 2004. Riyadh, Saudi Arabia: Saudi Cancer Registry.	Saudi Arabia	2004-2005
	Semnani S, Sadjadi A, Fahimi S, Nouraie M, Naeimi M, Kabir J, Fakheri H, Saadatnia H, Ghavamnasiri MR, Malekzadeh R. Declining incidence of esophageal cancer in the Turkmen Plain, eastern part of the Caspian Littoral of Iran: A retrospective cancer surveillance. Cancer Detect Prev. 2006; 30: 14-19.	Iran	1996-2000
	Shamseddine A, Sibai A-M, Gehchan N, Rahal B,	Lebanon	1998

© 2017 Karimkhani C et al. *JAMA Dermatology.*

	El-Saghir N, Ghosn M, Aftimos G, Chamsuddine N, Seoud M, Lebanese Cancer Epidemiology Group. Cancer Incidence in Postwar Lebanon: Findings from the First National Population-based Registry, 1998. Ann Epidemiol. 2004; 14(9): 663-8.		
	Sondrio Cancer Registry. Italy - Sondrio Cancer Registry Incidence, Mortality, and Survival 1998-2007.	Italy	1998-2007
	Surveillance, Epidemiology, and End Results (SEER) Program (www.seer.cancer.gov) Research Data (1973-2008), National Cancer Institute, DCCPS, Surveillance Research Program, Cancer Statistics Branch, released April 2011, based on the November 2010 submission.	United States	1973-2008
	Taiwan Cancer Registry. Taiwan Cancer Registry Incidence and Mortality 1980-2007.	Taiwan	1980-2007
	Ukrainian National Cancer Registry. Cancer in Ukraine. Kiev, Ukraine: Ukrainian National Cancer Registry.	Ukraine	2000-2011
	United Kingdom Cancer Information Service (UKCIS). United Kingdom - England Cancer Incidence Data 1990-2010.	United Kingdom	1990-2010
	Welsh Cancer Intelligence and Surveillance Unit. United Kingdom - Wales Cancer Incidence Report 2002-2006. Cardiff, Wales: Welsh Cancer Intelligence and Surveillance Unit, 2008.	United Kingdom	2002-2011
	Zimbabwe National Cancer Registry. Zimbabwe National Cancer Registry Annual Report 2005. Harare, Zimbabwe: Zimbabwe National Cancer Registry.	Zimbabwe	2005-2006

© 2017 Karimkhani C et al. *JAMA Dermatology.*
